# Design and synthesis of novel quinazolinones conjugated ibuprofen, indole acetamide, or thioacetohydrazide as selective COX-2 inhibitors: anti-inflammatory, analgesic and anticancer activities

**DOI:** 10.1080/14756366.2021.1956912

**Published:** 2021-08-01

**Authors:** Asmaa Sakr, Samar Rezq, Samy M. Ibrahim, Eman Soliman, Mohamed M. Baraka, Damian G. Romero, Hend Kothayer

**Affiliations:** a Department of Medicinal Chemistry, Faculty of Pharmacy, Zagazig University, Zagazig, Egypt; b Department of Pharmacology and Toxicology, Faculty of Pharmacy, Zagazig University, Zagazig, Egypt; c Department of Cell and Molecular Biology, University of Mississippi Medical Center, Jackson, MS, USA; d Mississippi Center of Excellence in Perinatal Research, University of Mississippi Medical Center, Jackson, MS, USA; e Women’s Health Research Center, University of Mississippi Medical Center, Jackson, MS, USA; f Cardiovascular-Renal Research Center, University of Mississippi Medical Center, Jackson, MS, USA

**Keywords:** COX-2 inhibition, quinazolinone, anti-inflammatory, molecular modelling, Anticancer

## Abstract

Novel quinazolinones conjugated with indole acetamide **(4a–c)**, ibuprofen (**7a–e),** or thioacetohydrazide (**13a,b,** and **14a-d**) were designed to increase COX-2 selectivity. The three synthesised series exhibited superior COX-2 selectivity compared with the previously reported quinazolinones and their NSAID analogue and had equipotent COX-2 selectivity as celecoxib. Compared with celecoxib, **4 b**, **7c**, and **13 b** showed similar anti-inflammatory activity *in vivo*, while **13 b** and **14a** showed superior inhibition of the inflammatory mediator nitric oxide, and **7** showed greater antioxidant potential in macrophages cells. Moreover, all selected compounds showed improved analgesic activity and **13 b** completely abolished the pain response. Additionally, compound **4a** showed anticancer activity in tested cell lines HCT116, HT29, and HCA7. Docking results were consistent with COX-1/2 enzyme assay results. *In silico* studies suggest their high oral bioavailability. The overall findings for compounds (**4a,b, 7c, 13 b,** and **14c**) support their potential role as anti-inflammatory agents.

## Graphical Abstract

**Figure:**
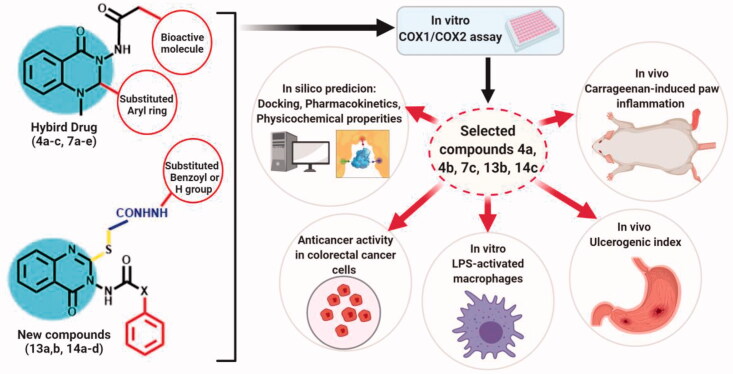

## 1. Introduction

Inflammation is a defensive mechanism as a response by the body to combat infections, chemicals or physical tissue injury^1^. The pathophysiology of pain is characterised by the release inflammatory mediators to initiate pain sensation, oedema, and other hallmarks of inflammation. Steroids are efficient in reducing inflammation and its associated pain however their use is complicated both by the wide range of adverse effects and by the necessity of their gradual withdrawal following the end of the treatment course^2^. While nonsteroidal anti-inflammatory drugs, (NSAIDs) such as indomethacin, ibuprofen, and diclofenac, have a relatively safe response profile, their long-term consumption is associated with severe gastrointestinal and renal side effects^3^^,^^4^.

Recent studies associated with the discovery of cyclooxygenase isozymes (COX-1/2) have helped advance the current understanding of inflammatory mechanisms^5^. The inhibition of COX-1 is the main cause of detrimental NSAID-associated gastrointestinal and renal side effects, thus “coxibs” were synthesised as selective inhibitors for COX-2, which are themselves associated with cardiovascular toxicity^6^^,^^7^. Recently, however, these adverse effects are expected to be drug-dependent rather than class-dependent^8^. Moreover, the COX-2 isozyme is overexpressed in human colon, gastric, hepatocellular, breast, ovarian, lung, and prostate cancers, and its inhibition is associated with a lower risk of cancer development^9^^,^^10^. In this way, COX-2 can be considered to be a potential anticancer target, specifically in cancer cells in which it is overexpressed. Consequently, there is a continuous need for the development of new selective COX-2 inhibitors with an improved gastric, and renal profiles, and fewer consequential side effects.

Recently, several compounds have been synthesised and evaluated as selective COX-2 inhibitors. Their common structural features involve the presence of two adjoining aryl rings attached to a central heterocyclic moiety (V-shape) with the possibility of introduction of a linker, either an ester^11^ or an amide^12^^,^^13^, between one of the aryl rings and the central heterocycle.

**Figure 1.**
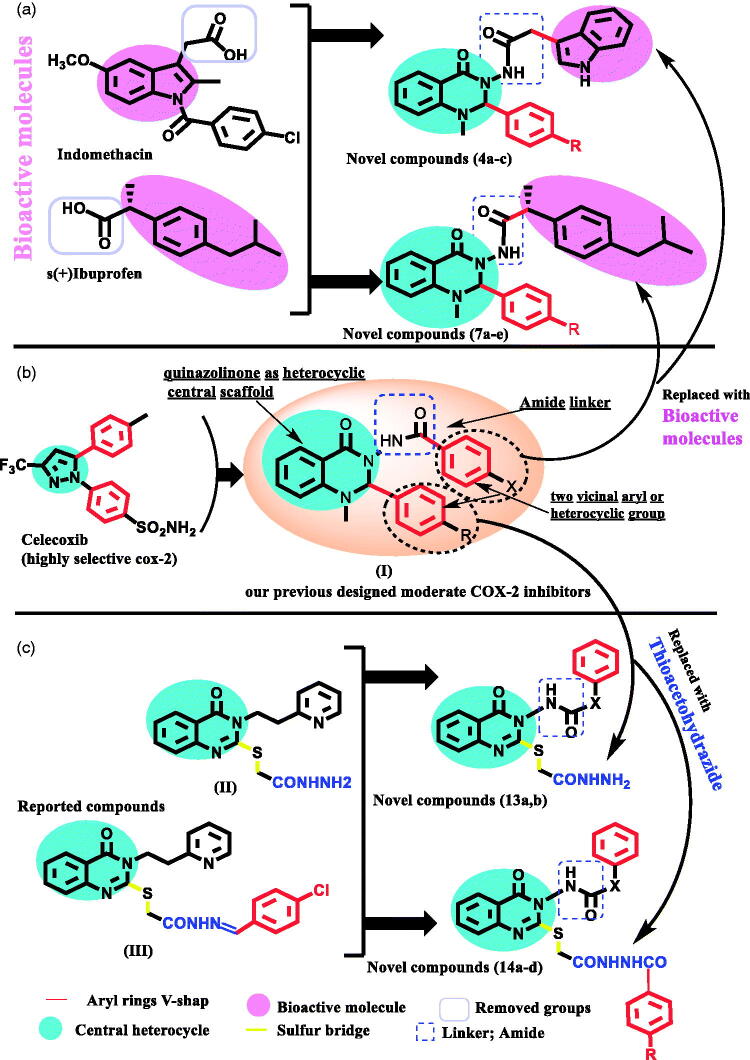
(a) Molecular design for hybrid bioactive novel compounds; (b) our previous designed moderate COX-2 from general structure selective COX-2; (c) Molecular design incorporates thioacetohydrazide novel compounds.

In continuation of our previous study, herein, we made further modifications to our previous successfully designed anti-inflammatory quinazolinones (**I**) (Figure 1), in order to increase their selectivity towards COX-2 inhibition^13^. In our current design, we kept the following: (a) the 2,3 diaryl-heterocyclic moiety (V-shape) to maintain the common structural integrity of selective COX-2 inhibitors^10^^,^^11^^,^^13^, (b) quinazolinone as the central heterocyclic ring due to its remarkable anti-inflammatory and analgesic activities^13^^,^^14^, and (c) the aryl ring at position 3 connected via an amide linker which may potentiate target interactions. Additionally, the introduction of the amide linker to the compounds allows for a bulkier structure, and thus, more favourable for COX-2 active site entry, which is approximately 20% larger than the COX-1 active site^12^^,^^13^.

**Scheme 1.**
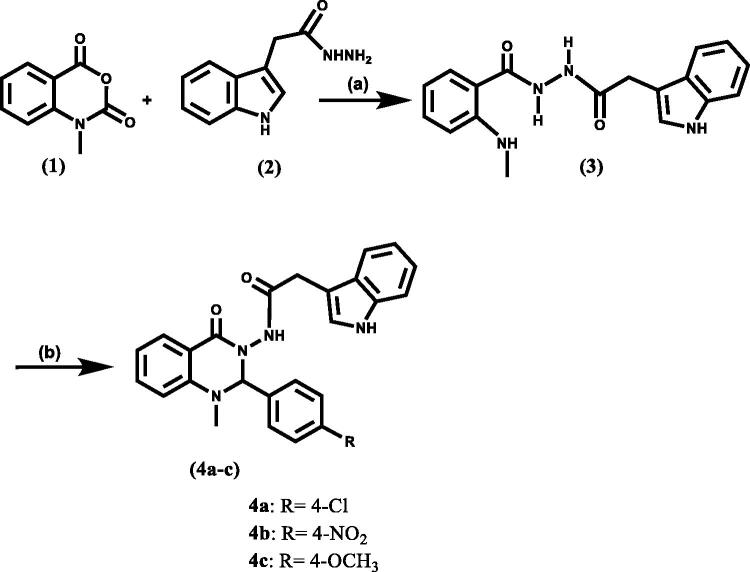
Synthetic route of target compounds, reagent, and conditions: (a) C_2_H_5_OH/2 ml glacial acetic acid, reflux, 12 h; (b) Appropriate aromatic aldehyde, glacial acetic acid, or C_2_H_5_OH/2 ml glacial acetic acid, reflux, 8–24 h.

**Scheme 2.**
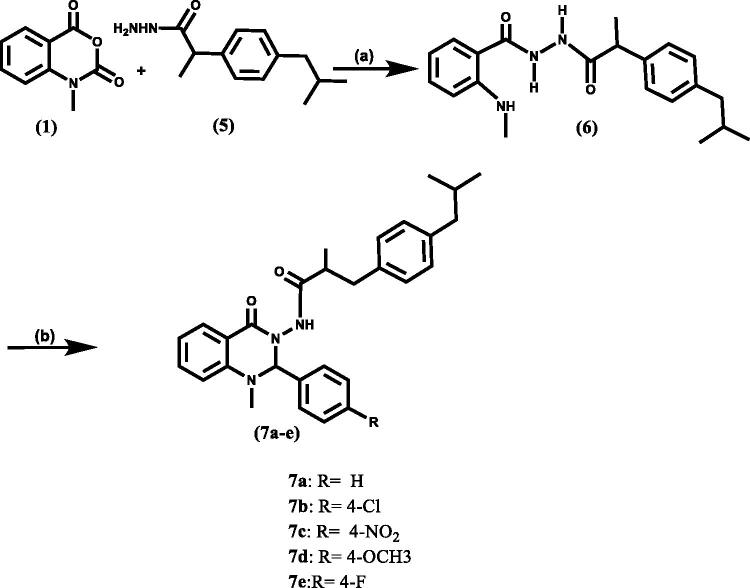
Synthetic route of target compounds, reagent, and conditions: (a) C_2_H_5_OH/2 ml glacial acetic acid, reflux, 3 h; (b) Appropriate aromatic aldehyde, glacial acetic acid, reflux, 8 h.

The approach of Schemes 1 and 2 is to explore the effect of incorporating a bioactive anti-inflammatory moiety (either indole acetamide (as indomethacin-alternative) or ibuprofen, respectively) (Figure 1), as the aryl ring attached to position 3 of the quinazolinone scaffold. The latter modification not only could increase COX-2 selectivity due to stoichiometric changes but also could help to explore further possible target interactions.

Both the classic non-selective COX inhibitors indomethacin and ibuprofen bind tightly to the COX active site. However, we faced some difficulty in synthesising the required indomethacin hydrazide, so our design was amended by incorporating indole-3-acetic acid instead of indomethacin. Aside from indomethacin, indole derivatives also possess significant anti-inflammatory activity^15–18^. Moreover, the benzoyl oxygen of indomethacin has been considered to be responsible for increased COX-1 affinity as its 4-bromobenzyl analogue exhibited high COX-2 selectivity, albeit without a benzoyl oxygen^19^. Therefore, in our design, we chose indole acetamide as an indomethacin alternative to overcome this problem. Additionally, to minimise the possible detrimental gastric effects, we masked the carboxylic acid group of both the indomethacin-alternative moiety and ibuprofen, which is responsible for salt bridge formation with Arg120 residue of the COX-1 active site causing their gastric mucosal side effects^13^^,^^19^.

**Scheme 3.**
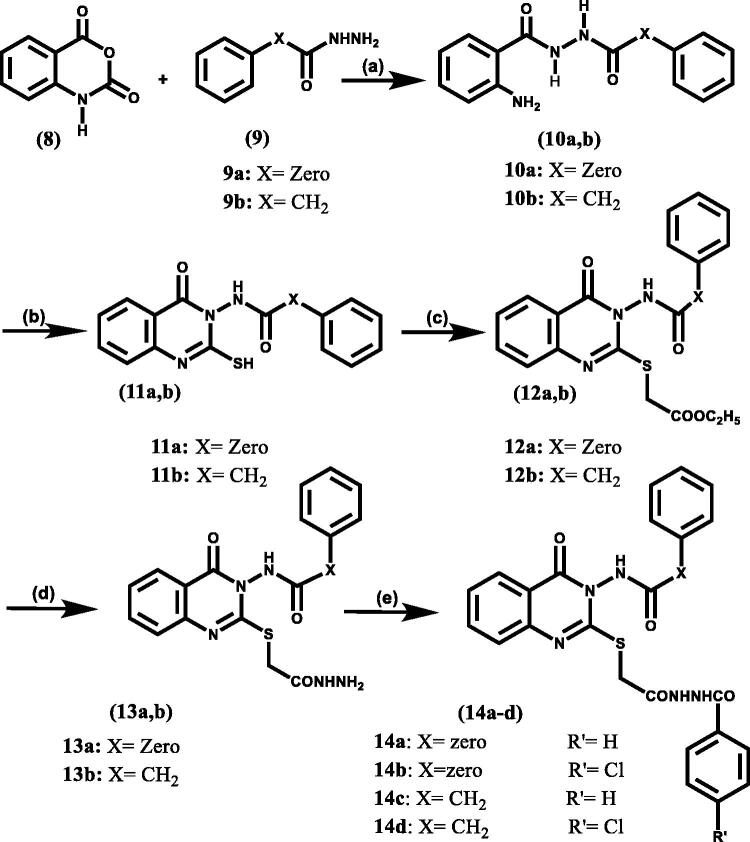
Synthetic route of target compounds, reagent, and conditions: (a) C_2_H_5_OH, 2 ml acetic acid, reflux, 8 h; (b) 2 equivalent KOH, excess CS_2_, 95% C_2_H_5_OH, reflux, 48 h; (c) Ethyl chloroacetate, 2 equivalent k_2_CO_3_, Anhydrous Acetone, rt, stirr, 3–8 h; (d) N_2_H_4_·H_2_O, C_2_H_5_OH, rt, stirr, 12 h; (e) 3.5 equivalent liquid benzoyl chloride derivatives, stirr, 1 equivalent (I_2_) pellets, wash Na thiosulphate sat.sol, NaHCO_3_ sat.sol, Brine sat.sol.

In Scheme 3, the pivotal feature of our approach was to study the shifting effect of phenyl ring located at position 2 of the quinazolinone moiety, via incorporation of a thioacetohydrazide linker, on both COX-2 selectivity and potency. Recent studies have shown advantages in the addition of a sulphur bridge at position 2 of the quinazolinone moiety in improving its anti-inflammatory activity^20^ (**II, III**) (Figure 1). Additionally, compounds containing an amide group showed superior *in*-*vivo* activity because they can easily cross the biological membrane^21^. Moreover, the hydrazide moiety at this position is able to make extra binding interactions with nearby amino acids within the COX active site.

Another focus of our investigation was to add flexibility between the quinazolinone scaffold and the aryl moiety at position 3 by the introduction of a rotatable bond next to the amide. This conformational freedom from the added flexibility may influence the potency and the selectivity of the newly synthesised compounds.

The newly synthesised compounds (**4a-c, 7a-e, 13a,b,** and **14a-d**) were evaluated for their COX-1/COX-2 selectivity using *in vitro* and *in vivo* assays to test their anti-inflammatory and antioxidant potential, and to investigate their ulcerogenic activity (UI) profile. To evaluate their possible anticancer activity through COX-2, we utilised cancer cell lines that have low, medium, or high COX-2 expression levels. Docking and *in silico* studies were used to predict their binding modes with COX-1/COX2, physicochemical properties, and pharmacokinetic profiles.

## 2. Materials and methods

### 2.1. Chemistry

Melting points (°C) were detected on a Gallenkamp melting point apparatus (London, UK), and are uncorrected. Elemental analyses, and mass spectra were performed in the Microanalytical Centre, Faculty of Science, Cairo University, Giza, Egypt. NMR spectra were performed in NMR Unit, Faculty of Pharmacy, Mansoura University, Egypt, or Microanalytical Centre, Faculty of Pharmacy, Cairo University, Egypt or Microanalytical Centre, Faculty of Science, Zagazig University, Egypt. NMR Spectra were recorded on Bruker high-performance Digital FT-NMR spectrometer avance III 400 MHz using dimethyl sulphoxide (DMSO)-d_6_ or pyridine (PYr)-d_6_ as solvent and tetramethylsilane (TMS) as an internal standard (chemical shift in *δ*, ppm). Mass spectra were determined using a GC/MS Shimadzu Qp-2010 plus (Shimadzu Corporation, Tokyo, Japan). Elemental analyses were established using the Vario EL-III (Elementar) CHNS analyser (Hanau, Germany). All reactions were continuously monitored by thin-layer chromatography (TLC) using silica gel 60 GF245 (E-Merck, Germany) and were detected by UV-lamp at wavelength (λ) 254 nm. Reagents and solvents were purchased from commercial suppliers and used as received. The isatoic anhydride was purchased from Sigma Aldrich (MO, USA) and ibuprofen was gifted from El-Qahera for pharmaceutical and chemical industries (Cairo, Egypt). The compound (**1**) was prepared as reported before ^22^. The hydrazides (**2, 5, 9a,** and **9b**) were synthesised starting from their corresponding acids using previously reported procedures^23–26^. Compounds (**10a, b**–**11a, b**) were synthesised according to procedures previously described in the literatures^27^^,^^28^.

#### 2.1.1. N'-(2-(1H-indol-3-yl)acetyl)-2(methylamino) benzohydrazide (3)

A mixture of indole-3-acetic acid hydrazide (**2**, 6 g, 0.031 mole), and N- methyl isatoic anhydride (**1**, 0.031 mole) in absolute ethanol (50 ml) acidified with glacial acetic acid (2 ml) was refluxed for 12 h; the formed precipitate upon reflux was filtered while hot to provide compound (**3**) as fluffy white powder.

Yield 70%, m.p. 187–191 °C, **^1^H NMR** (DMSO-d_6_) *δ*: 2.78 (3H, d, *J* = 4.92, NCH_3_), 3.63 (2H, s, CH_2_CO), 6.56 (1H, *t*, *J* = 7.44, benzo hydrazide-C_5_–H), 6.66 (1H, d, *J* = 8.32, benzo hydrazide-C_3_–H), 7.00 (1H, *t*, *J* = 7.4, indole-C_5_–H), 7.09 (1H, *t*, *J* = 7.16, indole-C_6_–H), 7.28 − 7.36 (3H, *m*, indole-C_2_–H, benzo hydrazide-C_7_–H, indole-C_7_ –H), 7.48 (1H, bs, NHCH_3_, exch.), 7.58 (1H, d, *J* = 7.64, indole-C_4_– H), 7.64 (1H, d, *J* = 7.8, benzo hydrazide-C_6_–H), 9.97 (1H, *s*, CONH, exch), 10.06 (1H, *s*, CONH, exch.), 10.89 (1H, *s*, indole NH, exch.).

#### 2.1.2. General method for synthesis of N-(2-(4-substitutedphenyl)-1-methyl-4-oxo-1,4-dihydroquinazolin-3(2H)-yl)-2-(1H-indol-3-yl)acetamide (4a-c)

A mixture of N'-(2-(1H-indol-3-yl)acetyl)-2-(methylamino) benzohydrazide. (methylamino) benzohydrazide (**3**, 4 g, 0.025 mole), and appropriate aromatic aldehyde in absolute ethanol acidified with glacial acetic acid (2 ml) was refluxed for 8–24 h. The reaction mixture concentrated to its half volume then:

For the chloride derivative (**4a**); after 8 h, the reaction mixture cooled, H_2_O drops were added then left in refrigerator for 6 h. The formed crystals were filtered and recrystallized from ethanol/H_2_O (5:2) under 20 °C, to obtain compound **4a.**

Or, for nitro derivatives (**4 b**); after 12 h, the yellow ppt. formed after concentration reaction mixture on hot was filtered, dried and washed several times with petroleum ether to yield pure compound **4 b.**

Or, for methoxy derivative (**4c**); after 24 h, the reaction mixture was cooled, H_2_O drops were added then left in refrigerator for 6 h. The formed semisolid was scratching with glass rod several times with diethyl ether, kept in closed container with diethyl ether and returned to refrigerator under 4 °C for 48 h, to yield **4c.**

#### 2.1.3. N-(2-(4-chlorophenyl)-1-methyl-4-oxo-1,4-dihydroquinazolin-3(2H)-yl)-2-(1H-indol-3-yl)acetamide (4a)

White crystals, yield 36%, m.p. 211–214 °C. **^1^H NMR** (DMSO-d_6_) *δ*: 2.77 (3H, *s*, NCH_3_), 3.58 (2H, *q*, *J* = 4.92, CH_2_CO), 5.78 (1H, *s*, NCHN), 6.70 (1H, d, *J* = 8.32, quinazolinone-C_8_–H), 6.86 (1H, *t*, *J* = 7.48, quinazolinone-C_6_–H), 6.98 (1H, *t*, *J* = 7.6, indole-C_5_–H), 7.09 (1H, *t*, *J* = 7.36, indole -C_6_–H), 7.23 (3H, d, *J* = 8.44, indole-C_2_–H, phenyl-C_2,6_–H), 7.35−7.47 (4H, *m*, indole-C_7_–H, phenyl-C_3,5_ –H, quinazolinone-C_7_–H), 7.52 (1H, d, *J* = 7.84, indole-C_4_–H), 7.80 (1H, d, *J* = 6.48, quinazolinone-C_5_–H), 10.43 (1H, *s*, CONH, exch.), 10.90 (1H, *s*, indole NH, exch.). ^13^**^ ^C NMR** (DMSO-d_6_) *δ*: 30.80 (CH_2_), 35.47 (NCH_3_), 79.37 (NCHN), 108.28 (ArCH), 111.76 (ArCH), 112.90 (ArCH), 115.03 (ArCH), 118.46 (ArCH), 118.83 (ArCH), 119.17 (ArCH), 121.50 (ArCH), 124.41 (ArCH), 127.59 (ArCH), 128.50 (ArCH), 128.99 (ArC), 129.05 (ArC), 134.19 (ArC), 135.13 (ArC), 136.45 (ArC), 136.51 (ArC), 147.03 (ArC), 160.85 (ArC), 170.13 (ArC). **MS**, *m/z*: 445 (M^+^), 447 (M^+^+2). Analysis calcd. for C_25_H_21_ClN_4_O_2_: C, 67.49; H, 4.76; N, 12.59. Found: C, 67.46; H, 4.80; N, 12.33.

#### 2.1.4. 2-(1 h-indol-3-yl)-N-(1-methyl-2-(4-nitrophenyl)-4-oxo-1,4dihydroquinazol in-3(2H)-yl)acetamide (4 b)

Yellow powder, yield 71%, m.p. 239–243 °C.**^1^H NMR** (DMSO-d_6_) *δ* 2.8 (3H, s, NCH_3_), 3.57 (2H, *s*, CH_2_CO), 5.96 (1H, *s*, NCHN), 6.73 (1H, d, *J* = 8.28, quinazolinone-C_8_–H), 6.89 (1H, *t*, *J* = 7.44, quinazolinone-C_6_–H), 6.96 (1H, *t*, *J* = 7.56, indole-C_5_–H), 7.08 (1H, *t*, *J* = 7.2, indole-C_6_–H), 7.22 (1H, bs, indole-C_2_–H), 7.35 (1H, d, *J* = 8.08, indole-C_7_–H), 7.46−7.50 (4H, m, indole-C_4_–H, quinazolinone-C_7_–H, phenyl-C_2,6_–H), 7.81 (1H, d, *J* = 7.64, quinazolinone-C_5_–H), 8.15 (2H, d, *J* = 8.68, phenyl-C_3,5_–H), 10.48 (1H, *s*, CONH, exch.), 10.89 (1H, *s*, Indole NH, exch.). ^13^**^ ^C NMR** (DMSO-d_6_) *δ* 30.85 (SCH_2_), 35.61 (NCH_3_), 79.06 (NCHN), 108.20 (ArCH), 111.75 (ArCH), 113.16 (ArCH), 115.14 (ArCH), 118.78 (ArCH), 119.13 (ArCH), 121.47 (ArCH), 124.18 (ArCH), 124.40 (ArCH), 127.58 (ArCH), 128.56 (ArCH), 128.61 (ArC), 129.05 (ArC), 135.20 (ArC), 136.51 (ArC), 144.59 (ArC), 146.91 (ArC), 148.34 (ArC), 160.74 (ArC), 170.15 (ArC). **MS**, *m*/*z*: 455 (M^+^). Analysis calcd. for C_25_H_21_N_5_O_4_: C, 65.93; H, 4.65; N, 15.38. Found: C, 65.91; H, 4.85; N, 15.23.

#### 2.1.5. 2-(1 h-indol-3-yl)-N-(2-(4-methoxyphenyl)-1-methyl-4-oxo-1,4-dihydroquin azolin-3(2H)-yl)acetamide (4c)

Yellow powder, yield 36%, m.p. 83–85 °C. **^1^H NMR** (DMSO-d_6_) *δ*: 2.74 (3H, *s*, NCH_3_), 3.58 (2H, d, *J* = 7.28, COCH_2_), 3.74 (3H, *s*, OCH_3_), 5.68 (1H, *s*, NCHN), 6.68 (1H, d, *J* = 8.28, quinazolinone-C_8_–H), 6.83 − 6.89 (3H, m, quinazolinone-C_6_–H, phenyl-C_3,5_ –H), 6.99 (1H, *t*, *J* = 7.36, indole- C_5_ –H), 7.08 − 7.14 (3H, m, indole-C_6_–H, phenyl-C_2,6_–H), 7.23 (1H, bs, indole-C_2_–H), 7.37 (1H, d, *J* = 8.32, indole-C_7_–H), 7.43 (1H, *t*, *J* = 7, quinazolinone-C_7_–H), 7.54 (1H, d, *J* = 7.84, indole-C_4_–H) , 7.80 (1H, d, *J* = 6.36, quinazolinone-C_5_–H), 10.39 (1H, *s*, CONH, exch.), 10.89 (1H, *s*, Indole NH, exch.). ^13^**^ ^C NMR** (DMSO-d_6_) *δ*: 30.76 (CH_2_), 35.38 (NCH_3_) 55.58 (OCH_3_), 79.74 (NCHN), 108.39 (ArCH), 111.75 (ArCH), 112.71 (ArCH), 114.34 (ArCH), 115.07 (ArCH), 118.15 (ArCH), 118.82 (ArCH), 119.21 (ArCH), 121.49 (ArCH), 124.39 (ArCH), 127.61 (ArCH), 128.44 (ArC), 128.79 (ArC), 129.53 (ArC), 135.00 (ArC), 136.51 (ArC), 147.24 (ArC), 160.23 (ArC), 161.00 (ArC), 170.07 (ArC). **MS**, *m*/*z*: 441 (M^+^). Analysis calcd. for C_26_H_24_N_4_O_3_: C, 70.89; H, 5.49; N, 12.72. Found: C, 70.58; H, 5.66; N, 12.50.

#### 2.1.6. N'-(2-(4-isobutylphenyl)propanoyl)-2-(methylamino) benzohydrazide (6)

A mixture of N-methyl isatoic anhydride (**1**, 20 gm, 0.0112 mole) and R/S ibuprofen hydrazide (**5**, 0.0112 mole) in absolute ethanol (250 ml) acidified by glacial acetic acid (4 ml) was refluxed for 3 h. The reaction mixture was cooled, and a white solid was precipitated immediately, filtered with suction system and dried to give the target compound **6**.

White powder, yield 85%, m.p. 148–150 °C. **^1^H NMR** (DMSO-d_6_) *δ*: 0.87 (6H, d, *J* = 6.6, CH_3_CHCH_3_), 1.39 (3H, d, *J* = 7, CH_3_CHCO), 1.79–1.84 (1H, *m*, CH_3_CHCH_3_), 2.42 (2H, d, *J* = 7.12, CHCH_2_), 2.78 (3H, d, *J* = 4.88, NCH_3_), 3.71 (1H, q, *J* = 7.04, CH_3_CHCO), 6.55 (1H, *t*, *J* = 7.4, benzo hydrazide-C_5_–H), 6.65 (1H, d, *J* = 8.32, benzo hydrazide-C_3_–H), 7.08 (2H, d, *J* = 7.64, ibuprofen phenyl-C_2,6_–H), 7.29 − 7.34 (3H, *m*, benzo hydrazide-C_4_–H, ibuprofen phenyl-C_3,5_ –H), 7.45 (1H, bs, NHCH_3_, exch.), 7.58 (1H, d, *J* = 7.48, benzo hydrazide-C_6_–H), 9.97 (1H, *s*, CONH, exch.), 10.05 (1H, s, CONH, exch.).

#### 2.1.7. General method for synthesis of N-(2-(4-substitutedphenyl)-1-methyl-4-oxo-1,4-dihydroquinazolin-3(2H)-yl)-2–(4-isobutylphenyl) propenamide (7a-e):

A mixture of N'-(2–(4-isobutylphenyl) propanoyl)-2-(methylamino) benzohydrazide (**6**, 8 g, 0.023 mole), and appropriate aromatic aldehyde (0.023 mole) in glacial acetic acid (10 ml), was refluxed at 120 °C for 8 h. The reaction mixture was concentrated to its half, cooled, drops of H_2_O were added, put in refrigerator for 6 h then:

For the chloride derivative compound (**7 b**); the white semi solid precipitated obtained was dried after removal of all supernatant, then dissolved in ethanol/H_2_O (5:2) and kept in refrigerator under 20 °C for 6 h. The resulted crystals were filtered and dried to give the desired compound **7 b**.

For the other derivatives compounds (**7a, 7c-e**), the yellow to brown semisolid precipitates formed after drying and removal of supernatant were dissolved in methanol (least amount) to form a concentrated solution and keep scratching with glass rod to give pure solid precipitated.

#### 2.1.8. 2-(4-Isobutylphenyl)-N-(1-methyl-4-oxo-2-phenyl-1,4-dihydroquinazolin-3(2H)-yl) propenamide (7a)

White powder, yield 40%, m.p. 85–89 °C. **^1^H NMR** (DMSO-d_6_) *δ*: 0.86 − 0.89 (6H, *m*, CH_3_CHCH_3_), 1.31 − 1.35 (3H, *m*, CH_3_CHCO), 1.81 − 1.86 (1H, *m*, CH_3_CHCH_3_), 2.43 (2H, *t*, *J* = 2.72, CHCH_2_), 2.71, 2.78 (3H, *s*, NCH_3_), 3.63 − 3.69 (1H, *m*, CH_3_CHCO), 5.57, 5.81 (1H, d, *s*, NCHN), 6.72 (1H, d, *J* = 8.28, quinazolinone-C_8_–H), 6.85 (1H, *q*, *J* = 4, quinazolinone -C_6_–H), 6.99 (1H, d, *J* = 7.4, phenyl-C_2_–H), 7.09 (2H, *t*, *J* = 8.6, ibuprofen phenyl-C_3,5_–H), 7.21 − 7.27 (4H, m, ibuprofen phenyl-C_2,6_–H, phenyl-C_4_–H, phenyl-C_6_–H), 7.29 − 7.35 (2H, *m*, phenyl-C_3,5_–H), 7.45 (1H, *q*, *J* = 5.2, quinazolinone-C_7_–H), 7.77 (1H, *q*, *J* = 7.56, quinazolinone-C_5_–H), 10.32, 10.39 (1H, *s*, CONH, exch.). ^13 ^**C NMR** (DMSO-d_6_) *δ*: 18.38 (COCH_3_), 22.61 (CH_3_CHCH_3_), 22.64 (CH_3_CHCH_3_), 30.08 (CH_3_CHCH_3_), 35.46 (NCH_3_), 42.66 (COCHCH_3_), 42.76 (CH_2_), 79.89 (NCHN), 112.58 (ArCH), 114.78 (ArCH), 118.23 (ArCH), 126.99 (ArCH), 127.51(ArCH), 128.43(ArCH), 128.98 (ArCH), 129.16 (ArCH), 129.55(ArCH), 135.01 (ArC), 137.36 (ArC), 138.56 (ArC), 139.83 (ArC), 147.38 (ArC), 160.89 (ArC), 172.77 (ArC).**MS**, *m*/*z*: 442 (M^+^). Analysis calcd. for C_28_H_31_N_3_O_2_: C, 76.16; H, 7.08; N, 9.52. Found: C, 75.77; H, 7.01; N, 9.30.

#### 2.1.9. N-(2-(4-chlorophenyl)-1-methyl-4-oxo-1,4-dihydroquinazolin-3(2H)-yl)-2–(4-isobutylphenyl) propenamide (7 b)

White crystals, yield 77%, m.p. 120–125 °C. **^1^H NMR** (DMSO-d_6_) *δ*: 0.88 (6H, d, *J* = 5.6, CH_3_CHCH_3_), 1.34 (3H, d, *J* = 7, CH_3_CHCO), 1.83–1.86 (1H, *m*, CH_3_CHCH_3_), 2.45 (2H, d, *J* = 7.12, CHCH_2_), 2.71 (3H, *s*, NCH_3_), 3.65 (1H, *q*, *J* = 7.04, CH_3_CHCO), 5.65 (1H, *s*, NCHN), 6.73 (1H, d, *J* = 8.32, quinazolinone-C_8_ –H), 6.87 (1H, *t*, *J* = 7.4, quinazolinone-C_6_–H), 7.05 (2H, d, *J* = 8.4, phenyl-C_2,6_–H), 7.11 (2H, d, *J* = 8, ibuprofen phenyl -C_3,5_–H), 7.17 (2H, d, *J* = 8.04, ibuprofen phenyl-C_2,6_–H), 7.31 (2H, d, *J* = 8.4, phenyl-C_3,5_–H), 7.45 (1H, *t*, *J* = 7.12, quinazolinone-C_7_–H), 7.79 (1H, d, *J* = 6.32, quinazolinone-C_5_–H), 10.31 (1H, *s*, CONH, exch.).**^13^C NMR** (DMSO-d_6_) *δ*: 18.44 (COCH_3_), 22.64 (CH_3_CHCH_3_), 22.67 (CH_3_CHCH_3_), 30.12 (CH_3_CHCH_3_), 35.36 (NCH_3_), 42.81 (COCHCH_3_), 44.72 (CH_2_), 79.18 (NCHN), 112.77 (ArCH), 114.78 (ArCH), 118.42 (ArCH), 127.50 (ArCH), 128.51 (ArCH), 128.96 (ArCH), 128.99 (ArCH), 129.29 (ArCH), 134.13 (ArC), 135.13(ArC), 136.40 (ArC), 138.83 (ArC), 139.93 (ArC), 147.27 (ArC), 160.84 (ArC), 172.73 (ArC). **MS**, *m*/*z*: 476 (M^+^), 478 (M^+2^). Analysis calcd. for C_28_H_30_ClN_3_O_2_: C, 70.65; H, 6.35; N, 8.83. Found: C, 70.38; H, 6.26; N, 8.46.

#### 2.1.10. 2-(4-Isobutylphenyl)-N-(1-methyl-2-(4-nitrophenyl)-4-oxo-1,4-dihydroquinazolin-3(2H)-yl) propenamide (7c)

Yellow powder, yield 72%, m.p. 111–116 °C. **^1^H NMR** (DMSO-d_6_) *δ*: 0.88 (6Η, d, *J* = 1.84, CH_3_CHCH_3_), 1.33 (3H, d, *J* = 7, CH_3_CHCO), 1.81–1.85 (1H, *m*, CH_3_CHCH_3_), 2.43 (2H, d, *J* = 7, CHCH_2_), 2.75 (3H, *s*, NCH_3_), 3.63 (1H, *q*, *J* = 7.04, CH_3_CHCO), 5.86 (1H, *s*, NCHN), 6.67 (1H, d, *J* = 8.32, quinazolinone-C_8_–H), 6.89 (1H, *t*, *J* = 7.44, quinazolinone-C_6_–H), 7.68 (2H, d, *J* = ibuprofen phenyl-C_3,5_–H), 7.16 (2H, d, *J* = 8.12, ibuprofen phenyl-C_2,6_–H), 7.34 (2H, d, *J* = 8.68, phenyl-C_2,6_–H), 7.48 (1H, *t*, *J* = 7.04, quinazolinone-C_7_–H), 7.81 (1H, d, *J* = 6.32, quinazolinone-C_5_–H), 8.09 (2H, d, *J* = 8.68, phenyl-C_3,5_–H), 10.36 (1H, *s*, CONH, exch.). ^13^**^ ^C NMR** (DMSO-d_6_) *δ*: 18.47 (COCH_3_), 22.58 (CH_3_CHCH_3_), 22.63 (CH_3_CHCH_3_), 30.09 (CH_3_CHCH_3_), 35.49 (NCH_3_), 42.91 (COCHCH_3_), 44.67 (CH_2_), 78.96 (NCHN), 113.08 (ArCH), 114.80 (ArCH), 118.84 (ArCH), 124.07 (ArCH), 127.53 (ArCH), 128.61 (ArCH), 129.17 (ArCH), 129.28 (ArCH), 135.30(ArC), 138.71 (ArC), 139.99 (ArC), 144.39 (ArC), 147.19 (ArC), 148.25 (ArC), 160.94 (ArC), 172.91 (ArC). **MS**, *m/z*: 486 (M^+^). Analysis calcd. for C_28_H_30_N_4_O_4_: C, 69.12; H, 6.21; N, 11.51. Found: C, 69.33; H, 6.03; N, 11.30.

#### 2.1.11. 2-(4-Isobutylphenyl)-N-(2-(4-methoxyphenyl)-1-methyl-4-oxo-1,4-dihydroquinazolin-3(2H)-yl) propenamide (7d)

Brown powder, yield 30%, m.p. 92–95 °C. **^1^H NMR** (DMSO-d_6_) *δ*: 0.88 (6H, d, *J* = 6.52, CH_3_CHCH_3_), 1.34 (3H, d, *J* = 6.84, CH_3_CHCO), 1.83 − 1.86 (1H, *m*, CH_3_CHCH_3_), 2.44 (2H, d, *J* = 7.04, CHCH_2_), 2.68 (3H, s, NCH_3_), 3.65 − 3.74 (4H, *m*, OCH_3_, CH_3_CHCO), 5.53 (1H, *s*, NCHN), 6.71 (1H, d, *J* = 8.32, quinazolinone-C_8_–H), 6.77 (2H, d, *J* = 7.64, phenyl-C_3,5_ –H), 6.84 (1H, *t*, *J* = 7.48, quinazolinone-C_6_– H), 6.92 (2H, d, *J* = 7.6, phenyl-C_2,6_–H), 7.11 (2H, d, *J* = 7.48, ibuprofen phenyl-C_3,5_–H) , 7.18 (2H, d, *J* = 7.24, ibuprofen phenyl-C_2,6_– H), 7.43 (1H, *t*, *J* = 7.84, quinazolinone-C_7_–H), 7.78 (1H, d, *J* = 7.64, quinazolinone-C_5_–H), 10.27 (1H, *s*, CONH, exch.). ^13^**^ ^C NMR** (DMSO-d_6_) *δ*: 18.44 (COCH_3_), 22.63 (CH_3_CHCH_3_), 22.68 (CH_3_CHCH_3_), 30.14 (CH_3_CHCH_3_), 35.32 (NCH_3_), 42.72 (COCHCH_3_), 44.72 (CH_2_), 55.54 (OCH_3_), 79.51 (NCHN), 112.55 (ArCH), 114.25 (ArCH), 114.84 (ArCH), 118.08 (ArCH), 124.07 (ArCH), 127.52 (ArCH), 128.39 (ArCH), 129.29 (ArCH), 129.51 (ArC), 134.99 (ArC), 138.95(ArC), 139.88 (ArC), 147.44 (ArC), 160.16 (ArC), 160.89 (ArC), 172.64 (ArC). **MS**, *m*/*z*: 472 (M^+^). Analysis calcd. for C_29_H_33_N_3_O_3_: C, 73.86; H, 7.05; N, 8.91. Found: C, 74.01; H, 6.85; N, 8.58.

#### 2.1.12. N-(2-(4-fluorophenyl)-1-methyl-4-oxo-1,4-dihydroquinazolin-3(2H)-yl)-2–(4-isobutylphenyl) propenamide (7e)

Yellowish brown powder, yield 35%, m.p. 90–95 °C. **^1^H NMR** (DMSO-d_6_) *δ*: 0.88 (6H, d, *J* = 6.4, CH_3_CHCH_3_), 1.34 (3H, d, *J* = 7.04, CH_3_CHCO), 1.82–1.86 (1H, *m*, CH_3_CHCH_3_), 2.44 (2H, d, *J* = 7.12, CHCH_2_), 2.70 (3H, *s*, NCH_3_), 3.65 (1H, *q*, *J* = 7.04, CH_3_CHCO), 5.63 (1H, *s*, NCHN), 6.73 (1H, d, *J* = 8.28, quinazolinone-C_8_–H), 6.86 (1H, *t*, *J* = 7.44, quinazolinone -C_6_–H), 7.06 − 7.11 (6H, *m*, phenyl-C_2,6_–H, ibuprofen phenyl-C_3,5_–H, phenyl-C_3,5_–H), 7.17 (2H, d, *J* = 8.04, ibuprofen phenyl-C_2,6_–H), 7.54 (1H, *t*, *J* = 7.08, quinazolinone-C_7_–H), 7.79 (1H, d, *J* = 6.28, quinazolinone-C_5_–H), 10.29 (1H, *s*, CONH, exch.).**^13^C NMR** (DMSO-d_6_) *δ*: 18.41 (COCH_3_), 22.62 (CH_3_CHCH_3_), 22.65 (CH_3_CHCH_3_), 30.12 (CH_3_CHCH_3_), 35.33 (NCH_3_), 42.80 (COCHCH_3_), 44.70 (CH_2_), 79.20 (NCHN), 112.74 (ArCH), 114.77 (ArCH), 115.67 (d, *J* = 22, ArCH), 118.37 (ArCH), 127.50 (ArCH), 128.50 (ArCH), 129.29 (ArCH), 129.32 (d, *J* = 8, ArCH), 133.73 (d, *J* = 3, ArC), 135.14 (ArC), 138.82 (ArC), 139.95 (ArC), 147.32 (ArC), 160.90 (ArC), 164.06 (d, *J* = 245.5, ArC), 172.75 (ArC). **MS**, *m*/*z*: 459 (M^+^), 461 (M^+2^). Analysis calcd. for C_28_H_30_FN_3_O_2_: C, 73.18; H, 6.58; N, 9.14. Found: C, 73.13; H, 6.43; N, 8.95.

#### 2.1.13. Method for synthesis of ethyl 2-((4-oxo-3-(benzamido)- 3,4-dihydroquinazolin-2-yl)thio) acetate (12a), and ethyl 2-((4-oxo-3– (2-phe nylacetamido)-3,4-dihydroquinazolin-2-yl)thio)acetate (12 b)

A mixture of compounds (**11a, b,** 0.01 mole) were mixed with anhydrous K_2_CO_3_ (4 g, 0.03 mole) and ethyl chloro acetate (1.84 g, 0.015 mole) in anhydrous acetone (25 ml) and stirred at 30 °C for 4–8 h^29^. The mixture was filtered and acetone distilled off. The remaining precipitates were stirred with distilled H_2_O, filtered and dried to be used in the next step without further purification.

#### 2.1.14. N-(2-mercapto-4-oxoquinazolin-3(4H)-yl)-2-phenylacetamide (11 b)

White powder, yield 70%, m.p. 279 − 280 °C **^1^H NMR** (DMSO-d_6_) *δ*: 3.69 (2H, *q*, *J* = 14.8, CH_2_), 7.26 (1H, *t*, *J* = 7.2, phenyl-C_4_–H), 7.34 (2H, *t*, *J* = 7.28, phenyl-C_3,5_–H), 7.38 − 7.44 (4H, m, phenyl-C_2,6_ –H, quinazolinone-C_8_,_6_–H), 7.80 (1H, *t*, *J* = 7.80, quinazolinone-C_7_–H), 7.98 (1H, d, *J* = 6.8, quinazolinone-C_5_–H), 11.17 (1H, *s*, CONH, exch.), 13.16 (1H, *s*, CSNH, exch.)^28^.

#### 2.1.15. Ethyl 2-((4-oxo-3-(2-phenylacetamido)-3,4-dihydroquinazolin-2-yl)thio)acetate) 12 b)

White powder, yield 84%, m.p. 107–108 °C.**^1^H NMR** (DMSO-d_6_) *δ* 1.23 (3H, *t*, *J* = 7.08, CH_3_), 3.77 (2H, *s*, COCH_2_), 4.01 (2H, *s*, S-CH_2_), 4.15 (2H, *q*, *J* = 7.04, CH_2_CH_3_), 7.29 (1H, *m*, phenyl-C_4_–H), 7.38 (4H, *m*, phenyl -C_2,6 3,5_–H), 7.51 (2H, *t*, *J* = 5.48, quinazolinone-C_6, 8_–H), 7.85 (1H, *t*, *J* = 6.96, quinazolinone-C_7_–H), 8.09 (1H, d, *J* = 6.96, quinazolinone-C_5_–H), 11.49 (1H, s, CONH, exch.).

#### 2.1.16. General method of preparing N-(2-((2-hydrazinyl-2-oxoethyl)thio)-4-oxoquinazolin-3(4H)-yl) benzamide (13a), and N-(2-((2-hydrazinyl-2-oxoethyl)thio)-4-oxoquinazolin-3(4H)-yl) 2-phenylacetamide (13 b)

A mixture of ethyl ester for compounds (**12a, b**, 4 g, 0.01 mole), and hydrazine hydrate (1.5 g, 0.03 mole) in absolute ethanol (20 ml) was stirred at 30 °C for 12 h. The white precipitates formed were filtered, washed with distilled H_2_O and dried off to give the pure target compounds without further purification.

#### 2.1.17. N-(2-((2-hydrazinyl-2-oxoethyl)thio)-4-oxoquinazolin-3(4H)-yl)benzamide (13a)

white powder, yield 87%, m.p. 208–213 °C.**^1^H NMR** (DMSO-d_6_) *δ*: 3.83 (1H, d, *J =* 14.44, SCH), 3.96 (1H, d, *J =* 14.44, SCH), 4.34 (2H, bs, NH_2_, exch.), 7.54 (1H, *t*, *J* 7.76, quinazolinone-C_6_–H), 7.61 − 7.72 (4H, *m*, benzoyl-C_3,5_, _4_–H, quinazolinone-C_8_–H), 7.88 (1H, *t*, *J* = 6.44, quinazolinone-C_7_–H), 8.00 (2H, d, *J* = 7.28, benzoyl-C_2,6_–H), 8.11 (1H, d, *J* = 7.88, quinazolinone-C_5_–H), 9.38 (1H, *s*, CONH, exch.), 11.15 (1H, bs, CONH, exch.). ^13^**^ ^C NMR** (DMSO-d_6_) *δ* 33.95 (SCH_2_), 119.74 (ArCH), 126.86 (ArCH), 126.90 (ArCH), 127.14 (ArCH), 128.32 (ArCH), 129.37 (ArCH), 131.47 (ArCH), 133.50 (ArC), 135.87 (ArC), 147.15 (ArC), 158.92 (ArC), 159.12 (ArC), 166.32 (ArC), 166.74 (ArC). **MS**, *m*/*z*: 370 (M^+^). Analysis calcd. for C_17_H_15_N_5_O_3_S: C, 55.28; H, 4.09; N, 18.96. Found: C, 55.14; H, 4.03; N, 18.76.

#### 2.1.18. N-(2-((2-hydrazinyl-2-oxoethyl)thio)-4-oxoquinazolin-3(4H)-yl)-2-phenylacetamide (13 b)

white powder, yield 83%, m.p. 210–215 °C.**^1^H NMR** (DMSO-d_6_), 3.78 (3H, d, *J =* 17.24, COCH_2_ + SCH), 3.89 (1H, d, *J =* 14.56, SCH), 4.32 (2H, bs, NH_2_, exch.), 7.30 (1H, *m*, phenyl-C_4_ –H), 7.34 − 7.40 (4H, *m*, phenyl -C_2,6_, _3,5_–H), 7.5 (1H, *t*, *J* = 7.28, quinazolinone-C_6_– H), 7.62 (1H, d, *J =* 8.08, quinazolinone-C_8_ –H), 7.85 (1H, *t*, *J* = 7.04, quinazolinone-C_7_–H), 8.08 (1H, d, *J* = 7, quinazolinone-C_5_–H), 9.36 (1H, *s*, CONH, exch.), 11.23 (1H, bs, CONH, exch.). ^13^**^ ^C NMR** (DMSO-d_6_) *δ* 33.89 (SCH_2_), 119.67 (ArCH), 126.78 (ArCH), 126.83 (ArCH), 127.10 (ArCH), 127.29 (ArCH), 128.84 (ArCH), 129.76 (ArCH), 134.90 (ArC), 135.79 (ArC), 147.05 (ArC), 158.72 (ArC), 158.85 (ArC), 166.77 (ArC), 170.55 (ArC). ^13^**^ ^C NMR** (Pyridine-d_6_) *δ* 34.48 (SCH_2_), 41.15 (CH_2_), 120.26 (ArCH), 125.86 (ArCH), 126.55 (ArCH), 127.03 (ArCH), 127.09 (ArCH), 128.68 (ArCH), 129.82 (ArCH), 134.69 (ArC), 134.82 (ArC), 147.40 (ArC), 159.12 (ArC), 159.34 (ArC), 167.96 (ArC), 171.21 (ArC). **MS**, *m*/*z*: 383(M^+^). Analysis calcd. for C_18_H_17_N_5_O_3_S: C, 56.39; H, 4.47; N, 18.27. Found: C, 56.53; H, 4.43; N, 18.47.

#### 2.1.19. General method for preparation of N-(2-((2-(2-(4-substitutedbenzoyl) hydrazinyl)-2-oxoethyl)thio)-4-oxoquinazolin-3(4H)-yl)-(2-phenylacetamide) or (benzamide) (14a-d)

The titled compounds were prepared as in the reported method^30^ with some modification as following: A mixture of substituted hydrazides compounds (**13a,b**, 1 g, 0.003 mole) and liquid benzoyl chloride derivatives (0.01 mole) were mixed using glass rod, then I_2_ pellets were added (0.1 g, 0.0003 mole) with continuous mixing for 5 min under fume cupboard. The brown semisolids formed were vigorously washed with saturated solutions of sodium thiosulphate, followed by adding sodium bicarbonate and stirring up to 5 h at 40 °C. The precipitates were filtered, washed with brine H_2_O then with petroleum ether and left to dry to give the desired pure compounds (**14a-d)**

#### 2.1.20. N-(2-((2-(2-benzoylhydrazinyl)-2-oxoethyl)thio)-4-oxoquinazolin-3(4H)-yl)benzamide (14a)

white powder, yield 78%, m.p. 194–198 °C.**^1^H NMR** (DMSO-d_6_) *δ* 4.03 (1H, d, *J* = 15, SCH), 4.12 (1H, d, *J =* 15, SCH), 7.48 − 7.65 (6H, *m*, benzoyl-C_3,5_, _4_–H, benzoyl-C_3,5_, _4_–H), 7.71 (1H, *t*, *J* = 7.4, quinazolinone -C_6_–H), 7.78 (1H, d, *J* = 8.08, quinazolinone-C_8_–H), 7.87 − 7.92 (3H, m, benzoyl-C_2,6_–H, quinazolinone-C_7_–H), 8.01 (2H, d, *J* = 7.2, benzoyl-C_2,6_–H), 8.12 (1H, d, *J* = 6.96, quinazolinone-C_5_ –H) , 10.40 (1H, *s*, CONH, exch.), 10.51 (1H, *s*, CONH, exch.), 11.87 (1H, *s*, CONH, exch.). ^13^**^ ^C NMR** (DMSO-d_6_) *δ* 33.83 (SCH_2_), 119.75 (ArCH), 126.90 (ArCH), 127.10 (ArCH), 127.16 (ArCH), 127.94 (ArCH), 128.32 (ArCH), 128.92 (ArCH), 129.39 (ArCH), 131.43 (ArCH), 132.32 (ArCH), 132.82 (ArC), 133.53 (ArC), 135.85 (ArC), 147.19 (ArC), 158.87 (ArC), 158.94 (ArC), 165.82 (ArC), 166.33 (ArC), 166.83 (ArC) **MS**, *m*/*z*: 474 (M^+^). Analysis calcd. for C_24_H_19_N_5_O_4_S: C, 60.88; H, 4.04; N, 14.79. Found: C, 60.60; H, 3.82; N, 15.06.

#### 2.1.21. N-(2-((2-(2-(4-chlorobenzoyl)hydrazinyl)-2-oxoethyl)thio)-4 oxoquinazolin-3(4H)-yl)benzamide (14 b)

white powder, yield 90%, m.p. 215–220 °C.**^1^H NMR** (DMSO-d_6_) *δ*: 4.07 (2H, *q*, *J* = 14.92, SCH_2_), 7.53 − 7.79 (7H, *m*, benzoyl-C_3,5_, _4_,–H, chloro benzoyl-C_3,5_–H, quinazolinone-C_6_, _8_–H), 7.89 − 7.94 (3H, *m*, benzoyl-C_2,6_–H, quinazolinone-C_7_–H), 8.01 (2H, d, *J* = 7.36, chloro benzoyl-C_2,6_–H), 8.13 (1H, d, *J* = 7.8, quinazolinone-C_5_ –H), 10.44 (1H, *s*, CONH, exch.), 10.61 (1H, *s*, CONH, exch.), 11.87 (1H, *s*, CONH,exch.).**^13^C NMR** (DMSO-d_6_) *δ*: 33.79 (SCH_2_), 119.74 (ArCH), 126.89 (ArCH), 127.09 (ArCH), 127.15 (ArCH), 128.33 (ArCH), 129.09 (ArCH), 129.18 (ArCH), 129.38 (ArCH), 129.88 (ArCH), 131.52 (ArC), 133.50 (ArC), 135.82 (ArC), 137.20 (ArC), 147.18 (ArC), 158.86 (ArC), 158.95 (ArC), 164.86 (ArC), 166.40 (ArC), 166.89 (ArC). **MS**, *m*/*z*: 507 (M^+^), 509 (M ^+ 2^). Analysis calcd. for C_24_H_18_ClN_5_O_4_S: C, 56.75; H, 3.57; N, 13.79. Found: C, 56.64; H, 3.66; N, 14.00.

#### 2.1.22. N-(2-((2-(2-benzoylhydrazinyl)-2-oxoethyl)thio)-4-oxoquinazolin-3(4H)-yl)-2-phenylacetamide (14c)

white powder, yield 99%, m.p. 235–240 °C.**^1^H NMR** (DMSO-d_6_) *δ*: 3.77 (2H, *s*, COCH_2_), 3.98 (1H, d, *J* = 15, SCH), 4.10 (1H, d, *J* = 15, SCH), 7.27 − 7.31 (1H, *m*, phenyl C_4_–H), 7.35 − 7.41 (4H, *m*, phenyl C_2,6, 3,5_–H), 7.48 − 7.53 (3H, *m*, benzoyl C_4_, _3,5_–H), 7.58 (1H, *t*, *J* = 7.32, quinazolinone C_6_ –H), 7.74 (1H, d, *J* = 8.04, quinazolinone C_8_–H), 7.86 − 7.89 (3H, *m*, quinazolinone C_7_–H, benzoyl C_2,6_–H), 8.09 (1H, d, *J* = 7.88, quinazolinone C_5_ –H), 10.38 (1H, *s*, CONH, exch.), 10.51 (1H, *s*, CONH, exch.), 11.49 (1H, *s*, CONH, exch.). ^13^**^ ^C NMR** (DMSO-d_6_) *δ* 33.81 (SCH_2_), 41.15 (CH_2_), 119.68 (ArCH), 126.83 (ArCH), 127.11 (ArCH), 127.31 (ArCH), 127.93 (ArCH), 128.71 (ArCH), 128.95 (ArCH), 129.47 (ArCH), 129.77 (ArCH), 132.36 (ArCH), 132.77, (ArC), 134.91 (ArC), 135.78 (ArC), 147.10 (ArC), 158.59 (ArC), 158.75 (ArC), 165.86 (ArC), 166.89 (ArC), 170.61 (ArC). **MS**, *m*/*z*: 487 (M^+^). Analysis calcd. for C_25_H_21_N_5_O_4_S: C, 61.59; H, 4.34; N, 14.37. Found: C, 61.56; H, 4.30; N, 14.38.

#### 2.1.23. N-(2-((2-(2-(4-chlorobenzoyl)hydrazinyl)-2-oxoethyl)thio)-4-oxoquinazolin-3(4H)-yl)-2-phenylacetamide (14d)

White powder, yield 98%, m.p. 221–226 °C.**^1^H NMR** (DMSO-d_6_) *δ*: 3.78 (2H, *s*, CH2), 4.07 (2H, q, *J* = 27.44, SCH2), 7.28 (1H, *t*, *J* = 6.76, phenyl- C_4_–H), 7.35 − 7.41 (4H, *m*, phenyl-C_2,6, 3,5_–H), 7.51 (1H, t, *J* = 7.56, quinazolinone-C_6_–H), 7.58 (2H, d, *J* = 8.28, benzoyl-C_3,5_–H), 7.73 (1H, d, *J* = 8.12, quinazolinone-C_8_–H), 7.86 − 7.92 (3H, *m*, quinazolinone-C_7_ –H, benzoyl-C_2,6_–H), 8.08 (1H, d, *J* = 7.88, quinazolinone-C_5_–H), 10.44 (1H, *s*, CONH, exch.), 10.63 (1H, *s*, CONH, exch.), 11.57 (1H, *s*, CONH, exch.).**^13^C NMR** (DMSO-d_6_) *δ*: 33.80 (SCH_2_), 119.69 (ArCH), 126.80 (ArCH), 127.11 (ArCH), 127.27 (ArCH), 128.83 (ArCH), 129.07 (ArCH), 129.15 (ArCH), 129.79 (ArCH), 129.91 (ArCH), 131.54 (ArC), 134.99 (ArC), 135.74 (ArC), 137.16 (ArC), 147.09 (ArC), 158.63 (ArC), 158.70 (ArC), 164.81 (ArC), 166.85 (ArC), 170.59 (ArC). **MS**, *m*/*z*: 522 (M^+^), 524 (M ^+ 2^). Analysis calcd. for C_25_H_20_ClN_5_O_4_S: C, 57.53; H, 3.86; N, 13.42. Found: C, 57.77; H, 3.58; N, 13.49.

#### 2.1.24. The unexpected new compound N-(4-oxoquinazolin-3(4H)-yl)-2-phenylacetamide (IV):

White crystalline powder, yield 40%, m.p. 277–280 °C.**^1^H NMR** (DMSO-d_6_) *δ*: 3.75 (2H, *s*, CH2), 5.85 (1H, *s*, NCHN), 7.28 (1H, *t*, *J* = 6.76, phenyl- C_4_–H), 7.35 − 7.40 (4H, *m*, phenyl-C_2,6_, _3,5_–H), 7.52 (1H, *t*, *J* = 7.72, quinazolinone-C_6_–H), 7.86 (1H, *t*, *J* = 7.2, quinazolinone-C_7_–H) , 8.12 (1H, d, *J* = 6.48, quinazolinone C_8_–H), 8.94 (1H, d, *J* = 8.48, quinazolinone-C_5_–H), 11.23 (1H, s, CONH, exch).

### 2.2. Biological activity

#### 2.2.1. *In vitro* COX-1 and COX-2 inhibitory assay

All the newly synthesised compounds were tested for their ability to inhibit COX-1 and COX-2 enzymes using a screening assay method. The colorimetric ovine COX-1/human recombinant COX-2 assay Kit (catalogue No. 560131, Cayman Chemicals Inc., Ann Arbour, MI, USA) was used according to the supplier’s instructions and previously reported studies^12^^,^^31^^,^^32^

The IC_50_ of inhibition of COX-1/COX-2 activities in three replicates was calculated and is presented as the average of three values ± SEM (*n* = 3). The standards: Celecoxib, ibuprofen, indomethacin, and diclofenac were used as reference drugs in the study, and the SI values were calculated as IC_50_ (COX-1)/IC_50_ (COX-2).

#### 2.2.2. Experimental animals

Adult male Wistar rats (200–250 g) were purchased from the Faculty of Veterinary Medicine, Zagazig (Egypt) and kept in the animal house facility at the Pharmacology Department, Faculty of Pharmacy, Zagazig University (Egypt) for one week before starting the experiments under standard conditions of light/dark cycle and 23–25 °C. Animals had free access to standard laboratory chow and water (*ad libitum*). All the experimental protocols were approved by the ethical committee at Zagazig University (ECAHZU), Faculty of Pharmacy, Zagazig University, Egypt, with a registration number (P15-12–2017) and are in accordance with the National Institutes of Health Guide for the Care and Use of Laboratory Animals 8th Edition^33^.

##### 2.2.2.1. *In vivo* anti-inflammatory assay

Carrageenan-induced rat paw oedema test was used to investigate the anti-inflammatory activity of the selected compounds (**4a,b, 7c, 13 b,** and **14c**) as previously reported^13^^,^^14^^,^^34^^,^^35^.

The rats were divided into nine groups (*n* = 5/group). All tested compounds were suspended in 1% Tween-80. Group 1, controls, were given the vehicle (1% Tween80, 10 ml/kg). The remaining groups each received one of the selected compounds (50 mg/kg) or one of the three reference drugs ibuprofen (20 mg/kg), indomethacin (20 mg/kg) or celecoxib (50 mg/kg). The rats were given the drugs 1 h before the injection of carrageenan solution (1% in 0.9% NaCl, 0.1 ml) (Sigma Aldrich, USA) in the sub-planter tissue of the right hind paw. The paw thickness (mm) was measured using a calliper before (0 h) and after carrageenan injection at 1, 2, 3, 4, 5 and 24 h. The inhibition of oedema thickness was calculated using the following formula (control– drug/control) × 100.

##### 2.2.2.2. Gastric acute ulcerogenic activity

The selected compounds (**4a, b, 7c, 13 b, 14c**) were evaluated for their ulcerogenic activity in rats, using the high ulcerogenic indomethacin and low ulcerogenic celecoxib as references. The rats from the previous experiment were fasted for 12 h., followed by the administration of additional doses of the selected compounds or the two references for two consecutive days. Six hrs after the last treatment, the animals were sacrificed, and their stomachs were removed, washed with saline solution (0.9%) and examined for ulceration using a magnifying lens. The ulcer scores were estimated according to the method prescribed by Kulkarni and as detailed in our previous studies^36–38^. The following scores were individually assigned to each lesion: normal coloured stomach, 0; red colouration, 0.5; spot ulcers, 1; haemorrhagic streaks, 1.5; ulcer >3 but <5 mm, 2; and ulcers >5 mm, 3.

The ulcer index (UI) was calculated according to the following equation:

$$[\mathrm{UI}=\mathrm{UN}+\mathrm{US}+\mathrm{UP}\times 10^{-1}],$$

where UN, US and UP are the ulcers number, severity score, and the percentage of animals with an ulcer, respectively.

##### 2.2.2.3. *In vivo* analgesic assay: acetic acid induced writhing test

The analgesic activity of the selected 5 compounds was measured using acetic acid-induced writhing pain model as previously reported^39^ using celecoxib as reference drug. Briefly, mice were divided into 7 groups (*n* = 5/group) that received either the vehicle (1% Tween 80, 10 ml/kg), the tested compounds (**4a, b, 7c, 13b,** and **14c**) (50 mg/kg, p.o) or celecoxib (50 mg/kg, p.o) 1 h prior acetic acid injection (0.7%, 1 ml/100 g, i.p). The number of writhes, manifested as extension of hind legs, constriction of abdomen, or turning of trunk was recorded within 30 min.

#### 2.2.3. Cell culture studies

Human colorectal cancer cell lines, HCT116 and HT29, and RAW 264.7 macrophages were obtained from ATCC (Manassas, VA). HCA7 colorectal cancer cell line was obtained from Sigma. HT29 and HCT116 cells were cultured in McCoy's 5 A medium (Sigma Aldrich, St. Louis, MO) containing 10% heat-inactivated foetal bovine serum (HI-FBS) (Gibco, USA), 100 μg/mL streptomycin (Invitrogen, USA), and 100 mg/ml penicillin (Invitrogen, USA)^40^. HCA7 and RAW 264.7 cells were cultured in Dulbecco's minimal essential media (DMEM, Invitrogen) containing 10% HI-FBS, sodium pyruvate (1 mM), penicillin (100 mg/mL), and streptomycin (100 mg/m)^41^^,^^42^.

##### 2.2.3.1. NO production in LPS-activated RAW 264.7 macrophages:

NO was measured using 4-amino-5-methylamino-2,7-difluorofluorescein diacetate (DAF-FM diacetate; Molecular Probes, USA). Briefly, RAW 264.7 cells were cultured in black 96-well plates (200,000 cells/mL, 100 µl/well) for 24 h. The cells were incubated with the individual test compounds or the reference drugs at different concentrations (6.5, 12.5, 25, 50, and 100 µM) for 2 h. at 37 °C followed by the incubation with LPS at a final concentration of 1 µg/mL for additional 20 h.^43^. The assay was then performed by washing the cells with phosphate-buffered saline (PBS) and incubating with 2 μM 2′,7′-dichlorofluorescein diacetate (DAF-FM) in serum-free medium. The fluorescence intensity, which is directly proportional to NO levels, was quantified, as detailed in our previous study^44^. IC_50_ values were calculated from the dose–response curves.

##### 2.2.3.2. ROS production in LPS-activated RAW 264.7 macrophages

The general probe of oxidative species 2,7-dichlorofluorescein diacetate (DCFH-DA) (Molecular Probes), was used to investigate the antioxidant potential of the test compounds following the induction of inflammation in RAW 264.7 by LPS. The cells were cultured, incubated with the different test compounds, and activated with LPS (1 µg/mL) as detailed above. The cells were then incubated with DCFH-DA (25 µM) and the fluorescence intensity, which is directly proportional to intracellular ROS levels was measured as detailed in our previous report^44^. IC_50_ values were calculated from the dose–response curves.

##### 2.2.3.3. MTS cell viability assays

Cells were cultured in 96‐well plates for 48 h and then treated with serum-free media containing different concentrations of the tested compounds (12.5, 25, 50, 100 and 150 µM). After 48 h., MTS reagent (Promega) was added as directed by the manufacturer, and then, the absorbance was measured at 495 nm. The absorbance is proportional to the number of viable cells. IC_50_ was calculated from the dose-response curves as described previously^45^ using GraphPad Prism 8 software (GraphPad Software, San Diego, CA).

### 2.3. Molecular docking and *in silico* study

#### 2.3.1. Docking study

Molecular docking of the selected compounds (**4a,b, 7c, 13b,** and **14c**) was performed to provide insight on their binding efficiencies with the active sites of COX-1 and COX-2. The molecular modelling studies of the compounds 2 D, and 3 D were carried out using Molecular Operating Environment MOE version 2018 software (Chemical Computing Group, Montreal, CA). The X-ray crystallographic complex structures of Cyclooxygenase-2 enzyme (COX-2) with ligand SC-558 (PDB entry 1CX2), and Cyclooxygenase-1 enzyme (COX-1) with ibuprofen (PDB code 1EQG) were downloaded from protein data bank website (http://www.rcsb.org). We used ibuprofen and SC-558 as references and both were redocked for validation. The protein structures were prepared after deletion of H_2_O molecules, repeated chains, and unwanted surfactants. Hydrogen atoms and partial charges were added using MOE quick preparation tool. Final compound data were prepared by adding hydrogen atoms, calculating partial charges, and minimising energy (MMF94). The docking poses were selected according to the best scoring functions.

#### 2.3.2. *In silico* prediction of pharmacokinetic and physiochemical properties

Compounds (**4a, b, 7c, 13b,** and **14c**) were subjected to screening assays for drug likeness and water solubility, Lipinski’s rule of five for drug Topological polar surface area (TPSA), oral bioavailability, toxicity and other pharmacokinetic by three software: Molinspiration Chemoinformatics server^46^, PreADMET calculator^47^ and the OSIRIS Property Explorer^48^. The resulting parameters were used to predict the *in vivo* behaviour of synthesised drugs compared with reference drugs. The values of TPSA are used to calculate the percentage of oral absorption (%ABS) using the following equation: %ABS = 109 − 0.345 TPSA^49^.

Osiris property explorer^48^ an online portal by Thomas Sander, Idorsia Pharmaceuticals Ltd, that provides predictions about the toxicity of any organic compound using a two-colour indicator; properties with a high degree of undesired effects are shown in red, whereas a green colour indicates drug-conforming behaviour.

### 2.4. Statistical analysis

The data were analysed using Graph Pad Prism 8 software (GraphPad Software, San Diego, CA, USA). One-way analysis of variance (ANOVA) or repeated-measures analysis of variance (RM-ANOVA) followed by Tukey’s *post hoc* test were used to state significance between groups. Data are presented as the mean ± SEM. Differences were considered significant at *p* < 0.05.

## 3. Results and discussion

### 3.1. Chemistry

**Figure 2.**
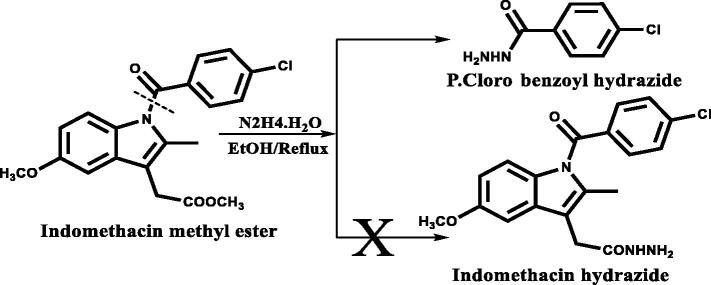
Illustrate the cleavage of indomethacin ester in hydrazinolysis^50^.

Two new series of hybrid bioactive molecules of 2,3 dihydroquinazolin-4(1H)-one with either indole-3- acetic acid or ibuprofen moiety were prepared using Schemes 1 and 2. We altered our hybrid construction of 2,3 dihydroquinazolin-4(1H) one with indole moiety instead of indomethacin upon experiencing difficulties in the preparation of indomethacin hydrazide via hydrazinolysis of its methyl ester. It was reported that this hydrazinolysis resulted in debenzoylation of indomethacin instead of formation of indomethacin hydrazide as illustrated in (Figure 2)^50^. The plethora of reported COX inhibitory activities associated with many indole derivatives^15–18^ encouraged us to utilise 2-(1H-indole-3- yl) acetohydrazide. This compound was prepared from indole-3-acetic acid as an alternative to indomethacin hydrazide. 

To incorporate a bioactive anti-inflammatory moiety (either indomethacin-alternative or ibuprofen, respectively), as the aryl ring attached to position 3 of the quinazolinone scaffold, the N-methyl-isatoic anhydride (**1**) was used as the starting material in the preparation of benzohydrazide derivatives (**3** and **6**) of the two series. Through condensation reactions with either 2-(1H-indole-3 yl) acetohydrazide or (R/S) 2–4-isobutyl phenyl propane-hydrazide, the intermediates **3** and **6** were synthesised. The chemical structure of intermediate **3** was investigated by ^1^H NMR to reveal four NH signals at *δ* = =evea (NHCH_3_), 9.97 (CONH), 10.06 (CONH), and 10.89 (Indole NH) ppm that are exchangeable by D_2_O. Similarly, the chemical structural investigation of intermediate **6** was identified by ^1^H NMR to indicate the presence of three NH singlet signals at *δ* = 7.45 (NHCH_3_), 9.97 (CONH), and 10.05 ppm (CONH) exchangeable with D_2_O, in addition to the presence of ^1^H NMR isobutyl profile; *δ* = 0.87 (6H, d, CH_3_CHCH_3_), 1.79–1.84 (1H, m, CH_3_CHCH_3_), and 2.42 (2H, d, CHCH_2_–C_6_H_4_ –).

To maintain the 2,3 diaryl-heterocyclic moiety (V-shape) of the final target bioactive compounds N-(2–(4-substitutedphenyl)-1-methyl-4-oxo-1,4-dihydroquinazolin-3(2H)-yl)-2-(1H-indol-3-yl) acetamide (**4a-c**) in Scheme 1 and N-(2–(4-substitutedphenyl)-1-methyl-4-oxo-1,4-dihydroquinazolin-3(2H)-yl)-2–(4-isobutylphenyl) propenamide (**7a-e**) in Scheme 2, cyclisation of the intermediates (**3** and **6**) was carried out using aromatic aldehydes in glacial acetic acid. The chemical structures of the final compounds **4a-c** were confirmed by ^1^H NMR, ^13 ^C NMR, mass spectra and elemental analysis. Using these methods, ^1^H NMR spectra indicated the cyclisation of the intermediate benzohydrazide (**3**) to 2,3 dihydroquinazolin-4 (1H)-one derivatives (**4a-c**), as only two NH signals presented in each of the final compounds representing CONH and indole NH, respectively, at *δ* = 10.43, 10.90 (**4a**), 10.48, 10.89 (**4 b**), and 10.39, 10.89 (**4c**) ppm. Additionally, the benzylic proton singlet signal for (**4a-c**) was at *δ* = 5.78, 5.96, and 5.68 ppm, respectively. Moreover, ^13 ^C NMR spectra revealed the presence of the characteristic C2-quinazoline carbon (NCHN) signal for (**4a-c**) at *δ* = 79.37, 79.06, and 79.74 ppm, respectively.

The chemical structures of the final compounds **(7a-e)** were identified by ^1^H NMR, ^13 ^C NMR, mass spectra, and elemental analysis. The ^1^H NMR spectra of these hybrids revealed the restriction of three NH signals of the intermediate **6** at *δ* = =t R spectraand 10.05 ppm to one NH signal of the final targets **(7a-e)** at *δ* ≈ 10.30 ppm with the benzylic proton appearing as a sharp singlet signal at *δ* ≈ 5.60 ppm. In addition to ^1^H NMR spectra of these compounds (**7a-e**) included the isobutyl profile. Moreover, ^13 ^C NMR spectra revealed the presence of the characteristic C2-quinazoline carbon (NCHN) signal for (**7a-e**) at *δ* ≈ 79 ppm.

**Figure 3.**
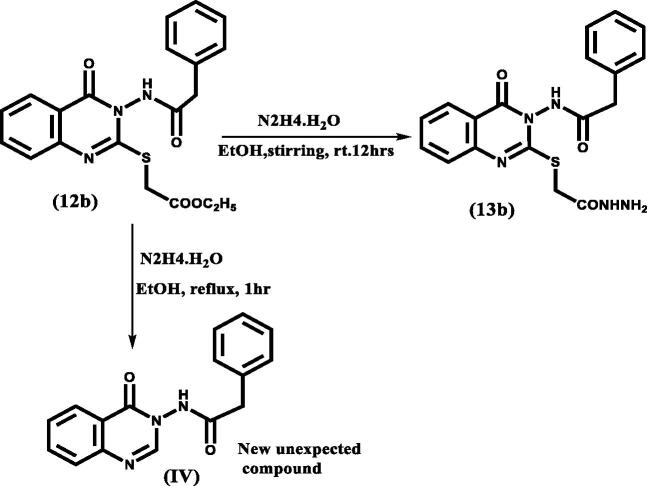
Different compounds for **12 b** resulted according to change in conditions.

In Scheme 3, the starting material was isatoic anhydride (**8**) which was condensed with either benzoic acid hydrazide or phenyl acetohydrazide to study the introduction of flexibility between the quinazolinone scaffold and the aryl moiety at position 3, producing benzohydrazide intermediates (**10 a, b**). Adding sulphur bridge at position 2 of the quinazolinone moiety was achieved by condensation of benzohydrazide intermediates (**10 a, b**) with CS_2_ in the presence of alcoholic KOH then acid neutralisation to give intermediates (**11a, b**) which were identified by their reported melting points^27^^,^^28^. The synthesis of intermediates (**12a, b**) was performed by alkylation of 2-mercapto-4(3H) quinazolinone derivatives (**11a, b**) with ethyl chloroacetate in anhydrous acetone. The final hydrazides (**13a, b**) that incorporated the targeted thioacetohydrazide linker were obtained by hydrazinolysis of the ester (**12a, b)** in mild conditions. We found that heating even as low as 40 °C led to the breakdown of the sulphur bridge and gave the unexpected compound **IV** as shown in the (Figure 3); its chemical structure was identified by ^1^H NMR (Supplementary data).

The ^1^H NMR spectra of compounds (**13a,b)** revealed the absence of triplet–quartette of ethyl group of the esters (**12a,b**) and the presence of three NH signals at *δ* = 4.34 (2H, NH_2_), 9.38 (1H, CONH), and 11.15 ppm (1H, CONH) for compound **13a** and at *δ* = 4.32 (2H, NH_2_), 9.36 (1H, CONH), and 11.23 (1H, CONH) for compound **13 b**. ^13 ^C NMR spectrum of compound **13a** revealed the presence of the SCH_2_ signal at *δ* = 33.95 ppm while that of compound **13 b** showed the SCH_2_ signal at *δ* = 34.48 ppm in addition to CH_2_ of phenylacetamide signal at *δ* = 41.15 ppm.

To maintain the 2,3 diaryl-heterocyclic moiety (V-shape), the final series N-(2-((2–(2-(4-substitutedbenzoyl) hydrazinyl)-2-oxoethyl) thio)-4-oxoquinazolin-3(4H)-yl) benzamide (XVIIa, b) and N-(2-((2–(2-(4-substitutedbenzoyl) hydrazinyl)-2-oxoethyl)thio)-4-oxoquinazolin-3(4H)yl)2phenylacetamide (**14a- d**) was formed by benzoylation of hydrazides (**13a, b**) using the catalytic acylation of amino compounds in presence of I_2_ as catalyst^30^.

The ^1^H NMR spectra of the final compounds **(14a- d)** indicates the absence of amino group signal from the hydrazides **(13a,b)** and the appearance of three amidic NH signals at *δ* = 10.40, 10.51, and 11.87 ppm for (**14a**), 10.44, 10.61, and 11.87 for (**14 b)**, 10.38, 10.51, and 11.49 ppm for (**14c)** and 10.44, 10.63, and 11.57 ppm for **(14d).**

### 3.2. Biological activity

#### 3.2.1. *In vitro* COX-1 and COX-2 inhibitory assay

All the final compounds **(4a-c, 7a-e, 13a,b and 14a-d)** were tested in comparison with indomethacin, ibuprofen, and celecoxib as reference drugs (Table 1). The efficacies of our compounds was evaluated by estimating the half-maximal inhibitory concentration (IC_50_) and the selectivity index (SI) values calculated as IC_50_ (COX-1)/IC_50_ (COX-2).

**Table 1.** *In vitro* COX-1/COX-2 inhibition assay

Compound Code	^a^COX-1 µM IC_50_	^a^COX-2 µM IC_50_	^b^Selectivity Index (SI)
Celecoxib	14.75 ± 0.15	0.04 ± 0.20	368.78
Indomethacin	0.09 ± 0.13	0.71 ± 0.15	0.13
Ibuprofen	4.13 ± 0.08	1.67 ± 0.25	2.47
**4a**	12.68 ± 0.11	0.04 ± 0.08	317.00
**4b**	6.93 ± 0.15	0.07 ± 0.22	99.00
**4c**	11.21 ± 0.13	0.03 ± 0.33	373.67
**7a**	12.76 ± 0.10	0.05 ± 0.13	255.02
**7b**	13.35 ± 0.10	0.04 ± 0.13	333.75
**7c**	14.73 ± 0.13	0.037 ± 0.20	398.11
**7d**	14.37 ± 0.16	0.04 ± 0.25	359.25
**7e**	11.85 ± 0.15	0.04 ± 0.23	296.25
**13a**	10.19 ± 0.14	0.04 ± 0.18	254.75
**13b**	16.92 ± 0.16	0.045 ± 0.18	373.77
**14a**	10.86 ± 0.20	0.03 ± 0.17	362.00
**14b**	12.98 ± 0.12	0.04 ± 0.29	324.5
**14c**	15.44 ± 0.17	0.04 ± 0.33	359.74
**14d**	12.56 ± 0.11	0.04 ± 0.38	314.00

^a^IC_50 in_ (µM) concentration as expressed as mean ± SEM, for three replications.

^b^Selectivity index=(COX-1 IC_50_/COX-2 IC_50_).

The incorporation of a bioactive anti-inflammatory moiety (either indomethacin-like (compounds **4a,c**) or ibuprofen (compounds **7a-e**) to position 3 of the quinazolinone scaffold, as well as the incorporation of a thioacetohydrazide linker at position 2 of the quinazolinone moiety (compounds **13a,b** and **14a-d**), not only succeeded in making our new compounds exhibit superior potency and selectivity towards COX-2 (SI = 254–398) over previously reported quinazolinones (**I**)^13^ (SI= 38.63–99.67), but also showed nearly the same COX-1 and COX-2 inhibitory activities (COX-1 IC_50,_ = 10.19 − 16.92 and COX-2 IC_50_= 0.03–0.05 µM) as that of celecoxib. Excitingly, the SI values of the new compounds are comparable to that of celecoxib (SI = 368.75) with the exception of compound **4 b** which has relatively lower COX-2 selectivity (SI= 99, COX-1 IC_50_ = 6.93 µM, COX-2 IC_50_ =0.07 µM).

For Scheme 1: compounds (**4a,c**) of indole bioactive molecule showed SI values (SI = 373–317) that are several-fold higher than that of indomethacin (SI = 0.13) and nearly the same as that of celecoxib. The difference in the *para* substituent of the phenyl ring attached to position 2 of the quinazolinone in this series caused a dramatic change in COX-1 inhibitory activity, as the *para* nitro derivative (**4 b**) showed a significantly greater potency (2-fold) towards COX-1 than compounds (**4a,c**) carrying *para* Cl or OCH_3_, respectively. The latter modification resulted in a decrease in **4 b** selectivity index (SI = 99.00) which is superior to that of indomethacin.

For Scheme 2: series (**7a-e**) of ibuprofen bioactive molecule showed SI (SI = 255–398) of approximately 150-fold greater than that of ibuprofen (SI = 2.47) and nearly the same as that of celecoxib. Conversely to the indole series **(4a-c)**, no significant change in COXs inhibitory activity was observed upon alternating the *para* substituent of the phenyl ring attached to position 2 of the quinazolinone. Importantly, the *para* nitro derivative (**7c**) showed slight improvement in SI value (SI = 398.11) compared to that of celecoxib (SI = 368.75).

For Scheme 3: Thioacetohydrazide containing series (**13a,b–14a-d**), all compounds showed SI values (SI = 254–373) that are nearly equal or slightly higher than that of celecoxib. Introducing a degree of flexibility between the quinazolinone scaffold and the aryl moiety at position 3 led to an improvement in the SI. Compounds with phenyl acetamide moiety (X = CH_2_) (**13 b** and **14c,d**) showed better SI values compared with their benzamide counterparts (**13a** and **14a,b**). This difference may also be due to the increase in their sizes to reduce COX-1 affinity.

In general, increasing the overall bulkiness of the quinazolinone scaffold either at position 3 (compounds **4a-c** and **7a-e**) or position 2 (compounds **13a,b** and **14a-d**) enhanced COX-2 inhibition activity and selectivity for COX-1. This may contribute to the larger size of the COX-2 active site and/or the ability of the inserted extension (indole-like, ibuprofen or thioacetohydrazide) to engage in additional intermolecular interactions within COX-2 active site.

Ibuprofen was better than indomethacin compounds. The incorporated bioactive anti-inflammatory moiety with COX-2 selectivity in the ibuprofen containing compounds **7 b,c** (with Cl (SI = 333) and NO_2_ (SI = 398)) showed superior SI values compared to their indomethacin-like containing counterparts **4a,b** (with Cl (SI = 317), with NO_2_ (SI = 99).

Both based on these favourable results and in order to limit animal use, we chose five compounds **4a,b, 7c, 13 b,** and **14c** for further *in vivo* investigation. Each of these compounds chosen represents those with the best SI in each series; **4 b** showed the lowest SI among all the synthesised compounds and was included for comparison. The potential ability to limit the production of nitric oxide (NO) and reactive oxygen species (ROS) as well as to identify anticancer activity was investigated *in vitro* using RAW 264.7 macrophages and colorectal cancer cell lines, respectively.

#### 3.2.2. *In vivo* anti-inflammatory assay

The carrageenan-induced rat paw oedema assay was used to test the anti-inflammatory activity of the selected compounds (**4a,b, 7c,** and **13 b, 14c**). Table 2 showed that the percent of inhibition of oedema for compounds **4 b** (with indomethacin-like moiety), **7c** (with ibuprofen moiety) and **13 b** (with thioacetohydrazide moiety) was nearly the same as that of celecoxib (47.60%) and ibuprofen (47.18%), and greater than that of indomethacin (33.81%). The greatest percent inhibition was 49.47% for compound **4 b** which has the indole ring as bioactive molecule and nitro group in the *para* position. The other indole derivative (**4a**) with a *para* chloro group achieved 33.40% inhibition of oedema, which was similar to that of indomethacin (33.81% inhibition) and lower than that of celecoxib (47.60% inhibition).

**Table 2.** *In vivo* anti-inflammatory activity in carrageenan-induced paw **o**edema in rat.

Tested compounds	Mean oedema thickness (mm) ± SEM							Average oedema inhibition%
	0h	1h	2h	3h	4h	5h	24h	
Control	0.00 ± 0.00	2.98 ± 0.09	3.66 ± 0.02	2.90 ± 0.18	2.71 ± 0.17	2.79 ± 0.12	0.96 ± 0.10	–
4a	0.00 ± 0.00	2.67 ± 0.24	2.90 ± 0.33*	2.14 ± 0.25	2.04 ± 0.24	1.39 ± 0.06*	0.54 ± 0.21	33.40
4b	0.00 ± 0.00	1.39 ± 0.09*	1.89 ± 0.25*	1.70 ± 0.17*	1.14 ± 0.22*	1.42 ± 0.28*	0.16 ± 0.08	49.47
7c	0.00 ± 0.00	1.79 ± 0.04*	2.21 ± 0.35*	1.90 ± 0.44*	1.62 ± 0.26*	1.55 ± 0.29	0.06 ± 0.04	45.37
13 b	0.00 ± 0.00	2.26 ± 0.30	2.58 ± 0.41*	2.67 ± 0.15	1.97 ± 0.29	1.61 ± 0.23*	0.45 ± 0.14	45.49
14c	0.00 ± 0.00	3.02 ± 0.35	2.89 ± 0.13*	2.42 ± 0.18	2.37 ± 0.19	2.34 ± 0.23	0.57 ± 0.09	31.86
Indomethacin	0.00 ± 0.00	2.00 ± 0.14*	2.21 ± 0.12*	1.82 ± 0.13*	2.02 ± 0.16	1.87 ± 0.18*	0.49 ± 0.10	33.81
Ibuprofen	0.00 ± 0.00	1.76 ± 0.07*	2.00 ± 0.15*	1.74 ± 0.08*	1.23 ± 0.16*	1.36 ± 0.18*	0.48 ± 0.15	47.18
Celecoxib	0.00 ± 0.00	1.27 ± 0.07*	1.51 ± 0.07*	1.49 ± 0.14*	1.39 ± 0.12*	1.19 ± 0.08*	0.53 ± 0.12	47.60

The thickness of paw oedema was measured at before (0) and 1, 2, 3, 4, 5 and 24 h. after the induction of inflammation. Data are mean ± SEM. The percentage inhibition of oedema thickness was calculated for each compound using the area under the curve of all time points (*n* = 5). *p˂0.05, significantly different from control.

The two compounds **4 b** and **7c** with *para* nitro substitution as bulk electron withdrawing group seems to have enhanced activity (nearly the same as celecoxib, 47.60% oedema inhibition) than that of compounds **4a** and **14c** with a *para* chloro or no substitution, respectively. In contrast to COX-2 selectivity, the percent of inhibition of oedema was slightly improved by incorporating an indomethacin-alternative entity as an active moiety (**4 b,** 49.47%**)** rather than incorporating ibuprofen one (**7c**, 45.37%).

For the class of thioacetohydrazides, the addition of phenyl ring in compound **14c** decreased the *in vivo* anti-inflammatory activity more than that of compound **13 b** which lacks the phenyl ring (31.86% vs. 45.49% oedema inhibition).

#### 3.2.3. Acute gastric ulcerogenic activity

All the tested compounds (**4a,b, 7c, 13 b,** and **14c**) had better ulcer index (UI) (3 − 8.26), than that of the reference compounds indomethacin (23.8) and ibuprofen (15). Compound **4a** that has the indole ring as bioactive molecule and a *para* chloro substitution, and an UI of 3 which is similar to the value of the reference drug celecoxib (2.4) (Table 3**)**

**Table 3.** Acute ulcerogenicity activity.

Compounds	Number of rats with ulcer	Lesion Incidence (%)	Average Ulcer number	Ulcer Index (UI)^a^
Control	0	0	0	Nil
**4a**	3	60	1.6	8.26
**4b**	1	20	0.8	3
**7c**	3	60	1	8
**13b**	2	40	0.4	4.8
**14c**	2	40	0.8	5.3
Indomethacin	5	100	12.4	23.8
Ibuprofen	5	100	3.8	15
Celecoxib	1	20	0.2	2.4

**^a^**The ulcer index (UI) was calculated according to the equation: (**UI = UN + US + UPX10^−1^**), (*n* = 5).

#### 3.2.4. *In vivo* analgesic assay: Acetic acid-induced writhing test

**Figure 4.**
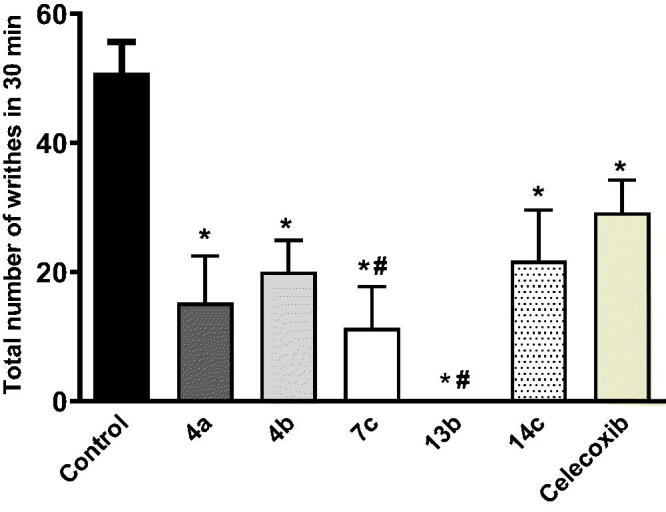
Effect of the tested compounds (50 mg/kg, p.o.) and celecoxib (50 mg/kg, p.o) on acetic acid-induced writhing in mice. Statistical analysis was performed using one way ANOVA followed by Tukey’s *post hoc* test. Data is expressed as mean ± SEM (*n* = 5). *p < 0.05 vs. control values. ^#^*p* < 0.05 vs. celecoxib.

The analgesic activity for the selected compounds (**4a,b, 7c, 13 b,** and **14c**) was evaluated using the acetic acid-induced writhing test using celecoxib as a positive control. The efficacy of the tested compounds as analgesic were measured by their ability to attenuate acetic acid-induced abdominal writhing. Notably, all the tested compounds showed better analgesic activity (range 0 − 21.75 writhes) than that of celecoxib (29.20 writhes) (Figure 4).

Interestingly, the thioacetohydrazide containing **13 b** showed exceptional analgesic activity as it was able to completely abolish the pain response with no writhes followed by compound **7c** (11.33 writhes), which has the ibuprofen as bioactive molecule and nitro group in *para* position, and showed 78% reduction in the pain response (writhes number).

Ibuprofen was favoured to indomethacin-like as the incorporated bioactive anti-inflammatory moiety to attenuate the abdominal pain as the ibuprofen conjugate **7c** showed better analgesic activity than its indomethacin-like conjugated counterparts **4a,b.** Similarly, the addition of phenyl ring in the thioacetohydrazide **14c** decreased the analgesic activity more than compound **13 b** which lacks the phenyl ring.

#### 3.2.5. Effects on NO and ROS production in LPS-activated RAW 264.7 macrophages cells

Lipopolysaccharides (LPS)-activated RAW 264.7 macrophage cells are a widely used *in vitro* model to study different inflammatory responses and to screen the mechanism of action of new anti-inflammatory candidates. Exposure of RAW 264.7 cells to the bacterial toxin LPS triggers a strong inflammatory status with the release of a number of inflammatory mediators including COX-2^43^. LPS also induces nitric oxide (NO) production by upregulating the inducible isoform of nitric oxide synthase which is required to maintain prolonged COX-2 expression^51^. Reactive oxygen species (ROS) are highly involved in the inflammatory response including LPS-mediated inflammation and can induce the production of a myriad of inflammatory cytokines^52^. Numerous *in vivo* and *in vitro* studies have shown that compounds with antioxidant potential are effective as anti-inflammatory and anti-cancer drugs^52–54^. All the tested compounds (**4a,b, 7c, 13 b,** and **14c**) inhibited the production of the inflammatory mediator NO with IC_50_ = 9.76 − 32.16 µM with some compounds having an effect that was greater than that of indomethacin (IC_50_= 25.28 µM). The two compounds with a thioacetohydrazide bridge **13 b** and **14c** (IC_50_ = 9.76 and 12.98 µM, respectively) showed superior scores compared to the three reference drugs celecoxib, ibuprofen, and indomethacin (IC_50_ = 19.51, 18.77 and 25.28, respectively). The two compounds with an indole bioactive molecule (**4a, b**) showed approximately 1.3-fold better IC_50_ values than that of indomethacin. The compound (**7c)** that was conjugated with ibuprofen as bioactive molecule showed IC_50_ of 23.41 µM which is slightly higher than that of ibuprofen (IC_50_=18.77 µM) (Table 4).

**Table 4.** *In vitro* NO and ROS production:

Compound	NO IC_50_ (µM)	ROS IC_50_ (µM)
**4a**	31.46 ± 1.08	29.67 ± 1.07
**4b**	32.16 ± 1.316	24.46 ± 2.06
**7c**	23.41 ± 1.29	9.228 ± 1.76
**13b**	9.76 ± 2.14	24.37 ± 1.43
**14c**	12.98 ± 1.36	16.18 ± 1.30
Celecoxib	19.51 ± 1.11	11.75 ± 1.11
Ibuprofen	18.77 ± 1.19	36.43 ± 1.45
Indomethacin	25.28 ± 1.01	68.92 ± 1.29

NO: nitric oxide; ROS: reactive oxygen species.

All the selected compounds (**4a,b, 7c, 13 b,** and **14c**) inhibited ROS production with IC_50_= 9.23 − 29.67 µM, indicating improved antioxidant activity compared with the two reference compounds ibuprofen (IC_50_= 36.43 µM) and indomethacin (IC_50_=68.92 µM). The most potent compound in reducing ROS levels was the ibuprofen-containing compound **7c** that showed an IC_50_ value, which was lower than that of celecoxib (9.22 vs. 11.75 µM) and of the thioacetohydrazide-containing compound **14c** with IC_50_ of 16.18 µM (Table 4). Notably, none of the tested concentrations were toxic to RAW 264.7 macrophages as tested by MTS cell bioavailability assay.

Again, incorporating ibuprofen as an active moiety was favoured to an indomethacin-alternative one in reducing both NO and ROS levels. The ibuprofen conjugate **7c** was the most potent in reducing both NO and ROS levels compared with its indomethacin-like conjugated counterparts **4a,b.**

#### 3.2.6. MTS Cell viability assays

NSAIDs of highly selective cyclooxygenase COX-2 inhibitory activity were proven by numerous experimental, epidemiologic, and clinical studies to be promising candidates as anticancer agents. COX-2 activity and expression are increased in colorectal cancer; NSAIDs, which inhibit COX-2 activity, could have the potential to inhibit colorectal carcinogenesis^55^^,^^56^. In order to explore the anticancer potential of the tested compounds owing to their COX-2 inhibition activity, we performed *in vitro* anticancer activity evaluation of the 5 tested compounds (**4a,b, 7c, 13 b,** and **14c**) against three colon cancer cell lines that express different levels of COX-2: The HT29 cell line, which moderately expresses COX-2, the HCT116 cell line, which lacks COX-2 expression, and the HCA7 cell line, which expresses high levels of COX-2^57^.

Compound **4a** has an indole ring as its bioactive molecule and a *para* chloro substitution and was the only compound that showed anticancer efficacy in all three tested cell lines HCT116, HT29 and HCA7 with IC_50_ values of 75.35, 15.42, and 137.3 µM, respectively. Interestingly, the active anticancer compounds **4a**, **4 b** (indole conjugates), and **7c** (ibuprofen conjugate) showed their maximal effect in the HT29 cell line which moderately expresses COX-2 with IC_50_ values of 15.42, 66.67, and 13.42 µM, respectively, indicating their effectiveness as COX-2 inhibitors (Table 5). As expected, all the tested compounds have relatively low cytotoxic activity against HCT116 cell line, which lacks COX-2 expression. Additionally, only compound **4a** was able to achieve a cytotoxic effect at concentrations less than 150 µM (IC_50_ = 137.3 µM) in HCA7 cell line, having high levels of COX-2 expression. This finding could be explained by the relatively high concentrations required to overcome high COX-2 activity in this particular cell line. Notably, neither thioacetohydrazide containing compounds **13 b** or **14c** showed any cytotoxic activities against any of the three tested cell lines.

**Table 5.** Cell viability assays

Compound	*HCT116 IC_50_ (μM)	*HT29 IC_50_ (μM)	*HCA7 IC_50_ (μM)
**4a**	75.35 ± 0.10	15.42 ± 0.06	137.3 ± 0.08
**4b**	>150	66.67 ± 0.07	>150
**7c**	>150	13.42 ± 0.17	>150
**13 b**	>150	>150	>150
**14c**	>150	>150	>150
Celecoxib	53.77 ± 0.05	5.82 ± 0.38	51.36 ± 0.04

*(HCT116), *(HT29), *(HCA7) are colon cancer cell lines that either scarcely, moderately or highly express COX-2, respectively.

### 3.3. Molecular modelling and *in silico* study

#### 3.3.1. Docking study

The docking of compounds (**4a,b, 7c, 13 b, and 14c**) into both the COX-1 (PDB code: 1EQG) and COX-2 (PDB code: 1CX2) binding sites were examined using Molecular Operating Environment (MOE) 2018 software^58^. For each compound, the pose with the best score was selected. The process was validated by re-docking SC-558 into the COX-2 active site and ibuprofen into the COX-1 active site, and their original conformations were reproduced (Score −9.39 and −7.56, RMSD: 1.34 A^0^ and 1.16 A^0^, respectively).

The original main interactions of the co-crystalized ligand SC-558 into the COX-2 active site are four hydrogen bonds with Tyr355, His90, Arg513, and Arg120 and one hydrophobic interaction with Ser353. While within COX-1 active site, ibuprofen forms three hydrogen bonds with Arg120 and Tyr355. The COX-2 active site is larger than that of COX-1 owing to the presence of an extra side pocket. This extra pocket is bordered by Tyr355, His90, Gln192, and Arg513 (the last residue is altered in COX-1 by His513). The high selective COX-2 inhibitors usually bind to Arg513 through the sulphone of their sulphonamide groups^13^^,^^59^. The docking results for the selected compounds showed better scoring within COX-2 active site (−8.54 to −6.26) than that within the COX-1 active site (-6.42 to −2.06).

**Figure 5.**
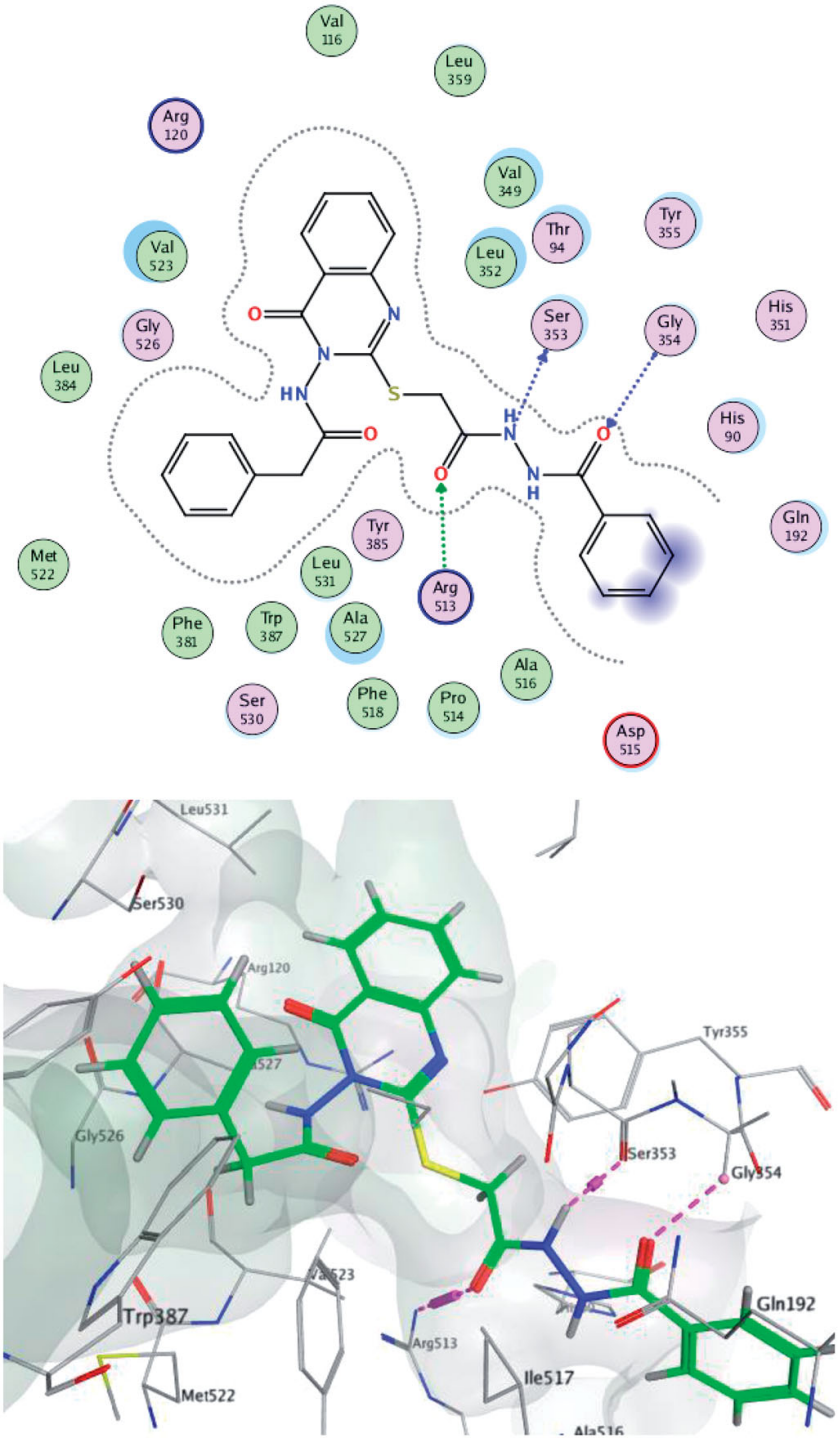
Two-/Three-dimensional (2-D, 3-D) binding interaction pattern of 14c in the binding site of 1CX2.

**Figure 6.**
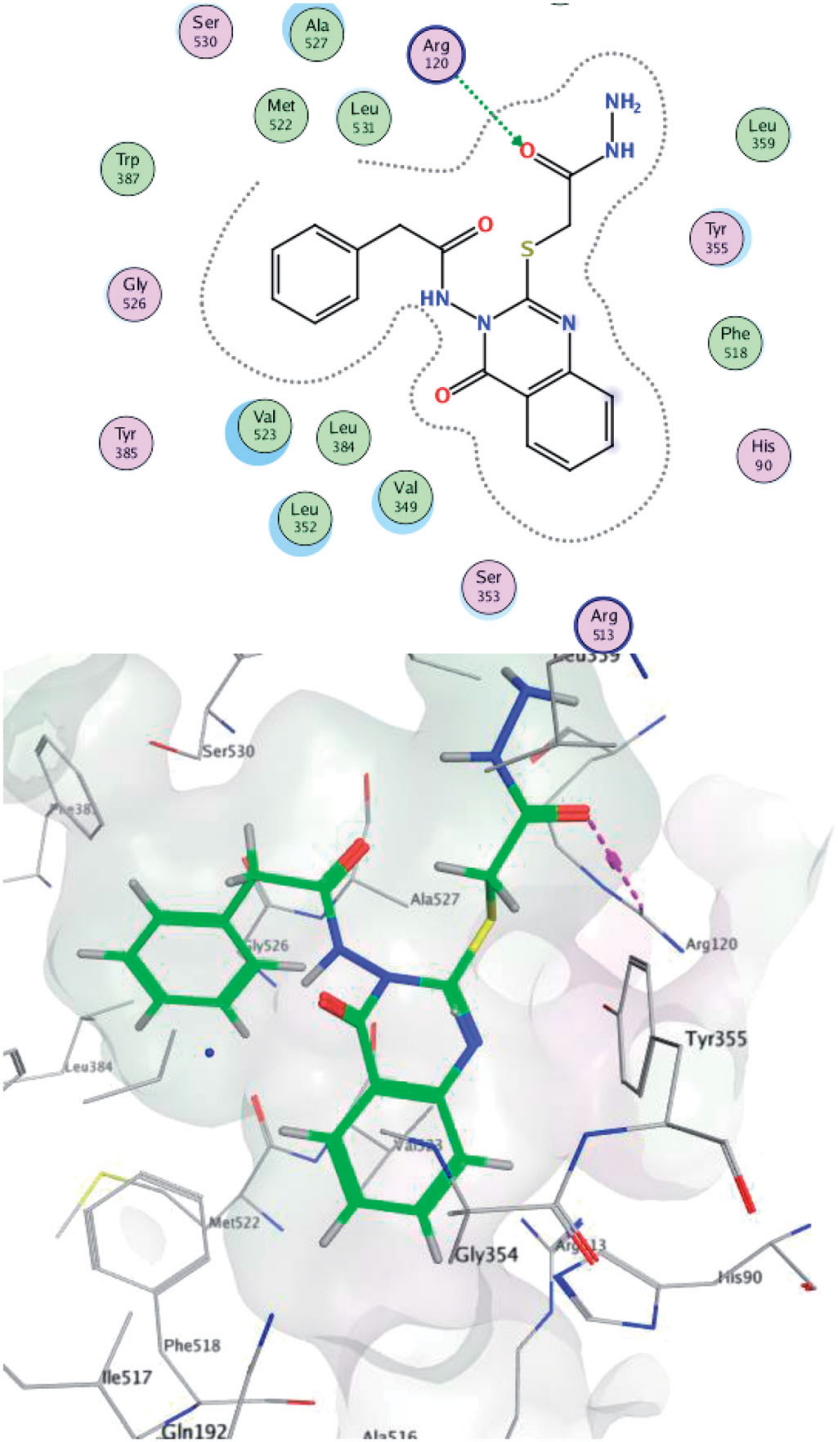
Two-/Three-dimensional (2-D, 3-D) binding interaction pattern of **13 b** in the binding site of 1CX2.

Compound **14c** showed the best score on COX-2 (-8.54) and was able to make hydrogen bonds with Arg513; these interactions have been reported to be responsible for the high selective inhibition of COX-2^19^^,^^60^ for residues Ser353 (one of the key residues in the binding mode of SC-558) and Gly354 (Figure 5). Compound **13 b** showed the highest score difference between COX-2 and COX-1 (−8.41, −2.06 respectively). These results are in line with the *in vitro* enzyme-binding assay and the high SI = 373 for the formation of hydrogen bond with Arg120 (one of the key residues in the binding mode of SC-558) within the COX-2 active site (Figure 6). Data provided in Supplementary Data Table 1.

**Figure 7.**
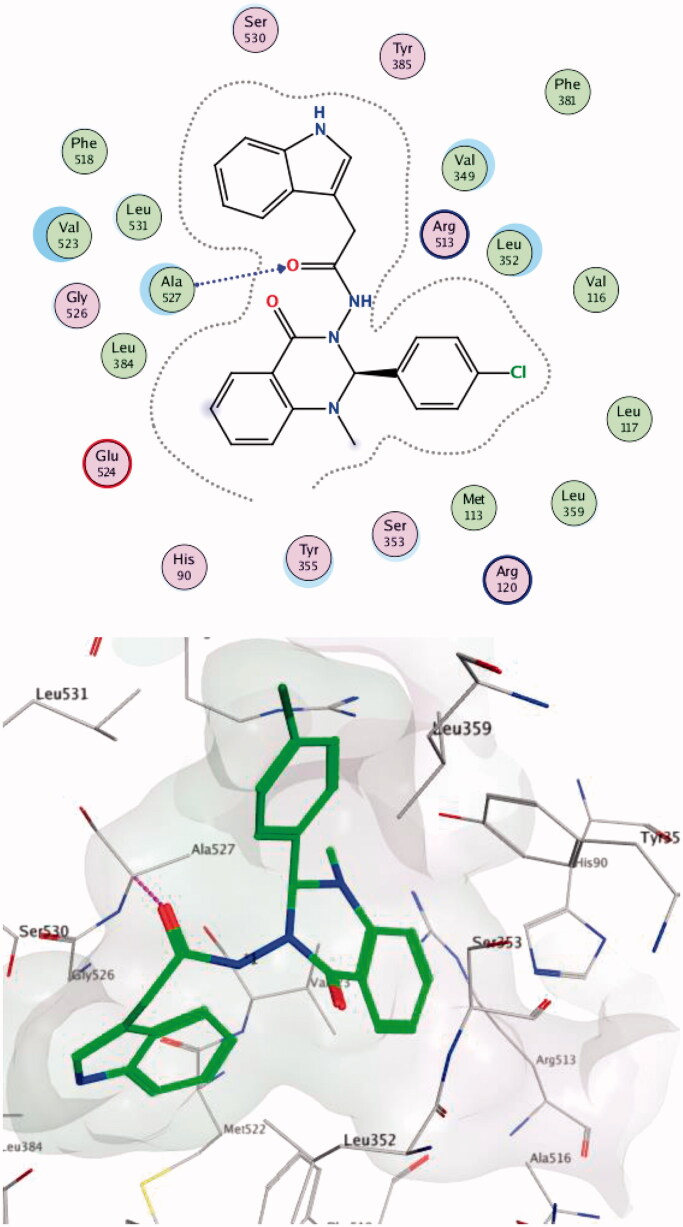
Two/Three-dimensional (2-D, 3-D) binding interaction pattern of **4a** in the binding site of 1CX2.

**Figure 8.**
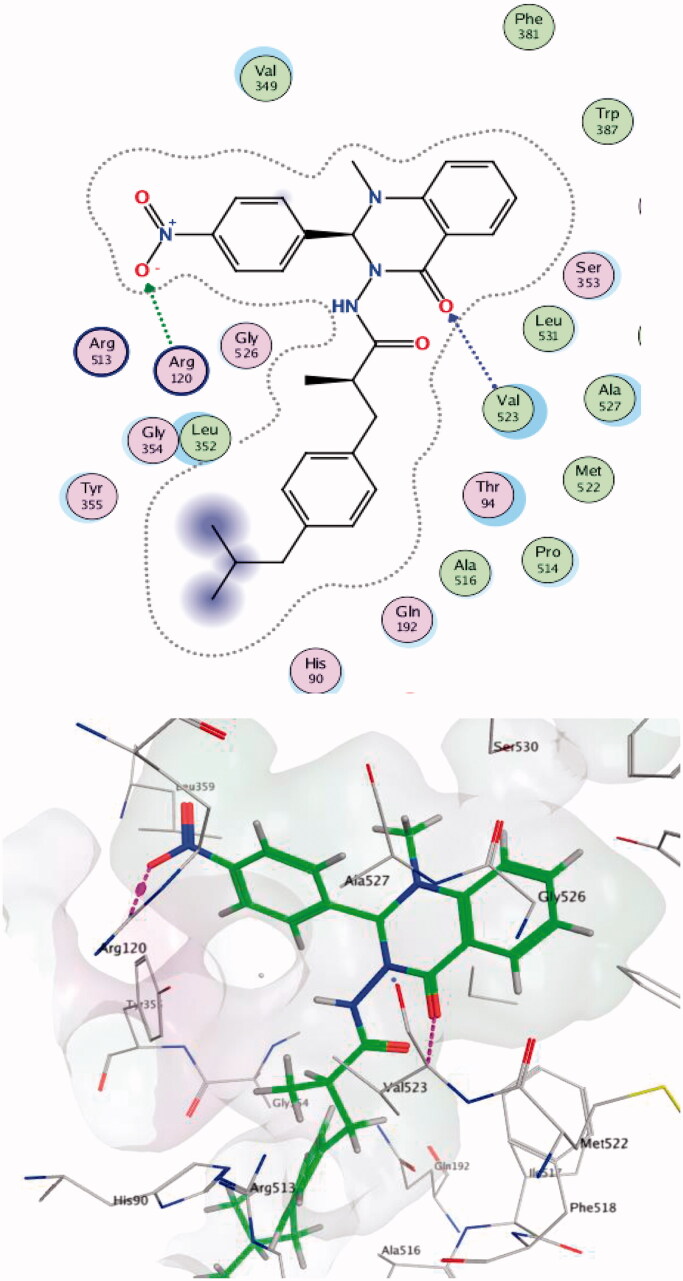
Two-/Three-dimensional (2-D, 3-D) binding interaction pattern of **7c** in the binding site of 1CX2.

**Figure 9.**
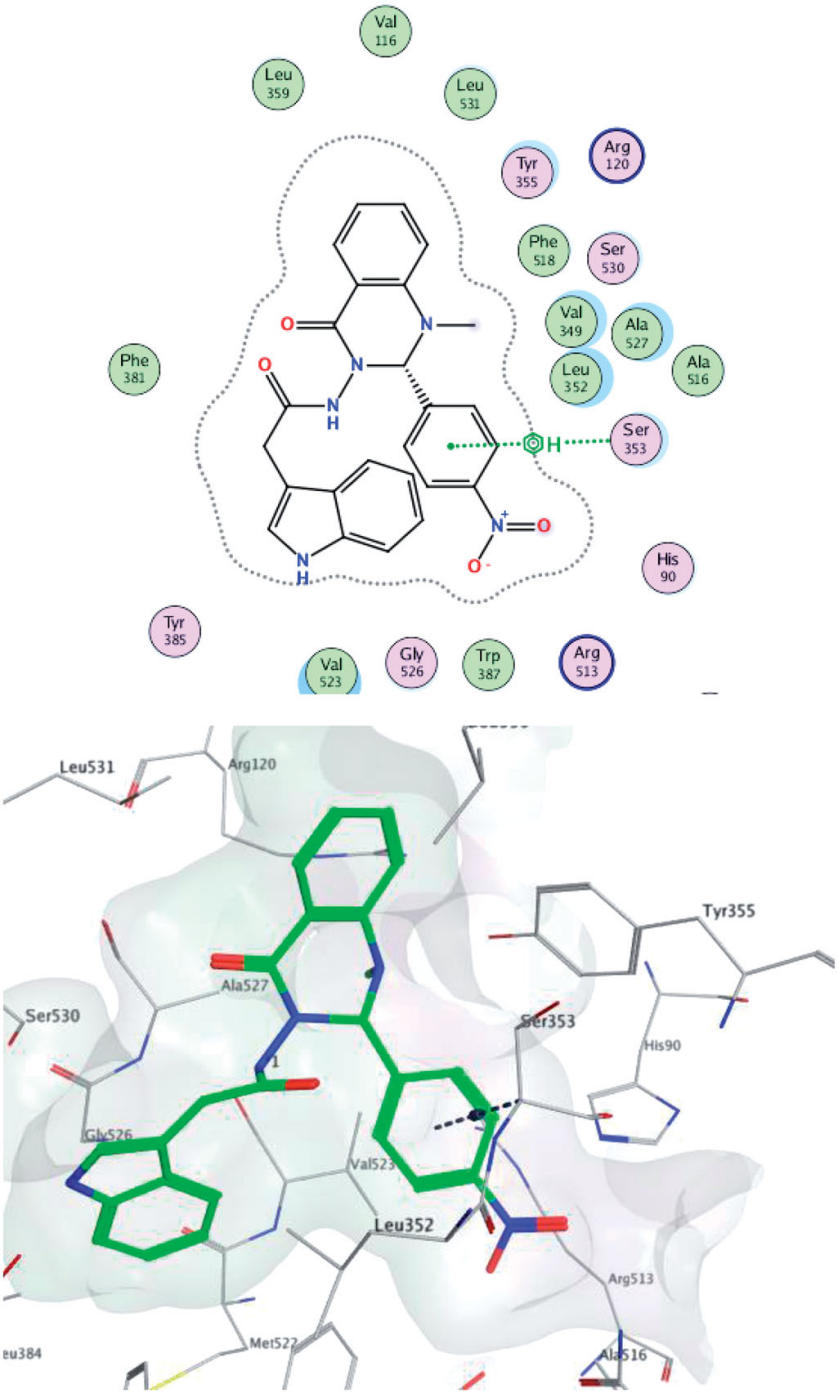
Two-/Three-dimensional (2-D, 3-D) binding interaction pattern of **4 b** in the binding site of 1CX2.

Within the COX-2 active site, compound **4a** formed a hydrogen bond with Ala527 while compound **7c** made two hydrogen bonds with Val523 and Arg120 (one of the key residues in the binding mode of SC-558). Compound **4 b** succeeded in making hydrophobic interactions with Ser353 (one of the key residues in the binding mode of SC-558) (Figures 7–9).

Regarding COX-1 docking results and scores, compound **7c** failed to make any interaction with the surrounding residues. Compounds **4a**, **4 b**, **13 b,** and **14c** made only one or two binding interactions with the surrounding residues including some with Arg120 (one of the key residues in the binding mode of ibuprofen) but with inferior scoring. This may be due to the bulkiness of the compounds which made them less preferred to fit into the COX-1 active site. Data provided in Supplementary Data Table 1.

The scoring for the poses of each compound with the COX-1/2 matches with our *in vitro* COX-1/COX-2 inhibition assay results and emphasise the occurrence of preferred binding between our compounds and COX-2 inhibition. Data provided in Supplementary Data Table 1.

#### 3.3.2. *In silico* prediction of pharmacokinetic and physiochemical properties

MOLINSPIRATION software^46^ was used to predict the oral bioavailability of the selected new compounds (**4a,b, 7c, 13 b,** and **14c**) through Lipinski’s rule of five and to determine the violation of the rule. The topological polar surface area (TPSA)(Å^2^) is another parameter that provides information about bioavailability. Compounds with TPSA values below 140–150 Å are expected to have good bioavailability; while compounds with TPSA values lower than 70 − 80 Å^2^ are expected to cross the blood–brain barrier (BBB) and effectively target the CNS. The TPSA was also used in the calculation of oral bioavailability (%ABS) by the following previously reported equation: (%ABS) = 109–0.345 TPSA^13^^,^^49^. The TPSA and number of rotatable bonds (NROTB) both affect oral bioavailability in our animal studies. Compounds are expected to have high oral bioavailability if the NROTB and TPSA values are ≤10 and 140 Å^2^, respectively. All data for selected new compounds provided in Supplementary Data Table 2.

The selected compounds **(4a,b, 13 b, and 14c)** did not violate Lipinski's rule, and therefore reveal suitable oral bioavailability. Only compound **7c** violated the parameters with log *P* = 5.80. Compounds **(4 b, 7c, 13 b,** and **14c)** had TPSA values (range from 98.47–122.19) of less than 140 Å^2^ and more than 80 Å^2^. These values indicate a diminished ability of these compounds to cross the BBB and therefore support the notion of limited potential CNS adverse effects. The compound **4a** had TPSA value of 68.44 and was an exception to this.

The Pre-ADMET calculator^47^ is used mainly for the prediction of permeability and absorption of synthesised drugs by two main models: the *in vitro* passive absorption through 2 parameter human epithelial colorectal adenocarcinoma cells (Caco2), and Mandin Dar by Canine Kidney (MDCK). Those two cell lines predict cell permeability as well as four other *in vivo* parameters: human intestinal absorption (HIA), blood brain barrier (BBB), plasma protein binding (PPB), and inhibition of cytochrome P450 2D6 (CYP2D6). (Supplementary Data Table 3).

All selected compounds (**4a, b, 7c, 13 b, and 14c**) have increased cell permeability for Caco2 over MDCK when compared with the reference compounds celecoxib, ibuprofen and indomethacin. For Caco2, the best compound was **4a** (34.04 nm/s) followed by **7c** (21.15 nm/s) and **4 b** (19.75 nm/s); they showed relatively lower permeability when tested for MDCK (0.06 − 0.42 nm/s). For HIA, all the selected compounds showed similar readings that ranged from 91.51% to 96.97% and were comparable to the references, again supporting suitable oral bioavailability.

Our tested compounds showed low potential to cross the BBB, with BBB permeability values (0.03–0.08) which are similar to that of celecoxib (0.03). The exception to this low potential was found for compounds **4a** and **4 b** which showed greater effectiveness for CNS penetration, having scored multiple-fold higher BBB permeability values of 4.07 and 0.31, respectively (Supplementary Data Table 3). Despite the low BBB penetration of celecoxib, it can reach concentrations in the CNS sufficient to effectively inhibit the COX-2 enzyme in that tissue. It is hypothesised that this mechanism is involved in celecoxib’s central pain control and may explain its therapeutic efficacy in ischaemic brain injury, malignant brain tumours and neurodegenerative diseases such as Parkinson disease, amyotrophic lateral sclerosis, and Alzheimer disease. It is of interest, therefore, to identify analogues of celecoxib that have a similar efficacy profile but with improved BBB permeability^61–63^. Interestingly, the permeability scores of compounds **4a** and **4 b** (4.07 and 0.31, respectively) predicted a greater BBB penetration compared to celecoxib (0.03). These compounds may resolve the CNS bioavailability limitations observed for celecoxib given these results. Further studies to explore the *in vivo* central anti-inflammatory potentials of both these compounds are currently in progress. This finding is especially relevant given that quinazolinone’s ability to cross BBB as an anticonvulsant therapeutic is well reported^64^^,^^65^.

Notably, the selected compounds showed strong PPB-binding capacity that ranges from of 90.25% to 100%. Compound **14c** is the one with the highest score as it showed 100% PPB binding (Supplementary Data Table 3).

Lastly, similar to the three reference drugs (celecoxib, ibuprofen and indomethacin), the selected compounds (**4a, b, 7c, 13 b,** and **14c**) do not inhibit the CYP2D6 enzyme; thus, they are expected to possess minimal drug-drug interactions either as inhibitors and/or inducers of this enzyme.

The results obtained by Osiris property explorer^48^, an online portal that predicts the possible toxicity of the tested compounds, showed that all our selected compounds exhibited drug-like behaviour with the exception of compound **13 b,** which is predicted to be associated with risk for tumorigenesis. Taken together, the results demonstrate that the newly synthesised compounds (**4a, b, 7c, 13 b,** and **14c**) display acceptable physicochemical properties and fulfil Lipinski’s rule of five. According to the pharmacokinetics predictions, these compounds are suitable future drug candidates.

## 4. Conclusion

Novel quinazolinones conjugates with either indole acetamide **(4a-c),** ibuprofen **(7a-e)** or thioacetohydrazide **(13a,b** and **14a-d)** have been designed to be selective COX-2 inhibitors. All the designed compounds exhibited potent and selective COX-2 inhibitory profiles. The docking studies were in line with the *in vitro* COX1/2 assays. The compounds **4 b, 7c**, and **13 b** showed nearly the same *in vivo* anti-inflammatory activity as ibuprofen and celecoxib and were more effective than indomethacin. Compounds **4a, b, 7c,** and **14c** showed superior analgesic activity than that of celecoxib while **13 b** showed the highest analgesic activity with complete abolishment of the pain response. Compounds **4a, b, 7c, 13 b,** and **14c** exhibited greater inhibitory effects on LPS-induced NO and ROS production in RAW 264.7 macrophage cells than that of ibuprofen and indomethacin. Moreover, compared to celecoxib, compounds **13 b** and **14a** showed greater inhibition of NO release and compound **7 C** showed higher antioxidant potential (via inhibition of ROS production). The cell viability assay for anticancer activity revealed that compounds **4a**, **4 b,** and **7c** had acceptable cytotoxic activity against HT29 cells, a cell line with moderate expression of COX-2 (IC_50_ values = 13.42–66.67 µM). Collectively, our findings demonstrate that compounds **4a, b, 7c, 13 b,** and **14c** represent potential candidates as selective COX-2 inhibitors with promising *in vivo* and *in vitro* anti-inflammatory and antioxidant activities. Additionally, compounds **4a and 7c** showed an additional promising anticancer activity. Moreover, the *in silico* physicochemical and pharmacokinetic studies for these compounds showed promising results with excellent oral bioavailability, lower potential for drug-drug interactions, and overall acceptable physicochemical properties that fulfilled Lipinski’s rule of five. Interestingly, compound **4a** and **4 b** exhibited higher estimated BBB permeability compared with celecoxib. Due to this enhanced property, these compounds may be better able to overcome limitations to CNS bioavailability observed for celecoxib and to extend their clinical use as central inflammatory therapeutic targets. The findings of the current study suggest that compounds **4a, b, 7c, 13 b,** and **14c** are all suitable potential drug candidates.

## Supplementary Material

Supplemental Material
