# Supplementary material for: Design and synthesis of novel quinazolinones conjugated ibuprofen, indole acetamide, or thioacetohydrazide as selective COX-2 inhibitors: anti-inflammatory, analgesic and anticancer activities
Source: J Enzyme Inhib Med Chem. 2021 Aug 1;36(1):1810–28. doi: 10.1080/14756366.2021.1956912 (PMC8330735; doi:10.1080/14756366.2021.1956912)
Supplement: Supplemental Material [file IENZ_A_1956912_SM5001.pdf]

Design and synthesis of novel quinazolinones conjugated ibuprofen, indole acetamide or thioacetohydrazide as selective COX-2 inhibitors: Anti-inflammatory, analgesic and anti-cancer activities

Asmaa Sakr[a], Samar Rezq[b,c,d,e,f], Samy M. Ibrahim[a], Eman Soliman[b], Mohamed M. Baraka[a], Damian G. Romero[c,d,e,f] and Hend Kothayer[a1]

[a] *Department of Medicinal Chemistry, Faculty of Pharmacy, Zagazig University, Egypt.*
[b] *Department of Pharmacology and Toxicology, Faculty of Pharmacy, Zagazig University, Egypt.*
Departments of [c]Cell and Molecular Biology, [d]Mississippi Center for Excellence in Perinatal Research, [e]Women's Health Research Center, [f]Cardio Renal Research Center, University of Mississippi Medical Center, Jackson, MS, USA

***Correspondence:** Hend Kothayer, Department of Medicinal Chemistry, Faculty of Pharmacy, Zagazig University, 44511, Zagazig, Egypt.

E-mail: hendo1311@hotmail.com or HKElhamalawy@pharmacy.zu.edu.eg

Tel.: 00201224578259 Fax: 0020552303266

Keywords: COX-2 inhibition; quinazolinone; anti-inflammatory; molecular modeling; Anticancer.

[1] Corresponding author. Tel. 00201224578259; E-mail: hendo1311@hotmail.com or hkelhamalawy@pharmacy.zu.edu.eg

Asmaa Alaa_H_IN-1

Microanalytical Unit - FOPCU - NMR laboratory
www.pharma.cu.edu.eg dir-mau.fopcu@pharma.cu.edu.eg

BRUKER

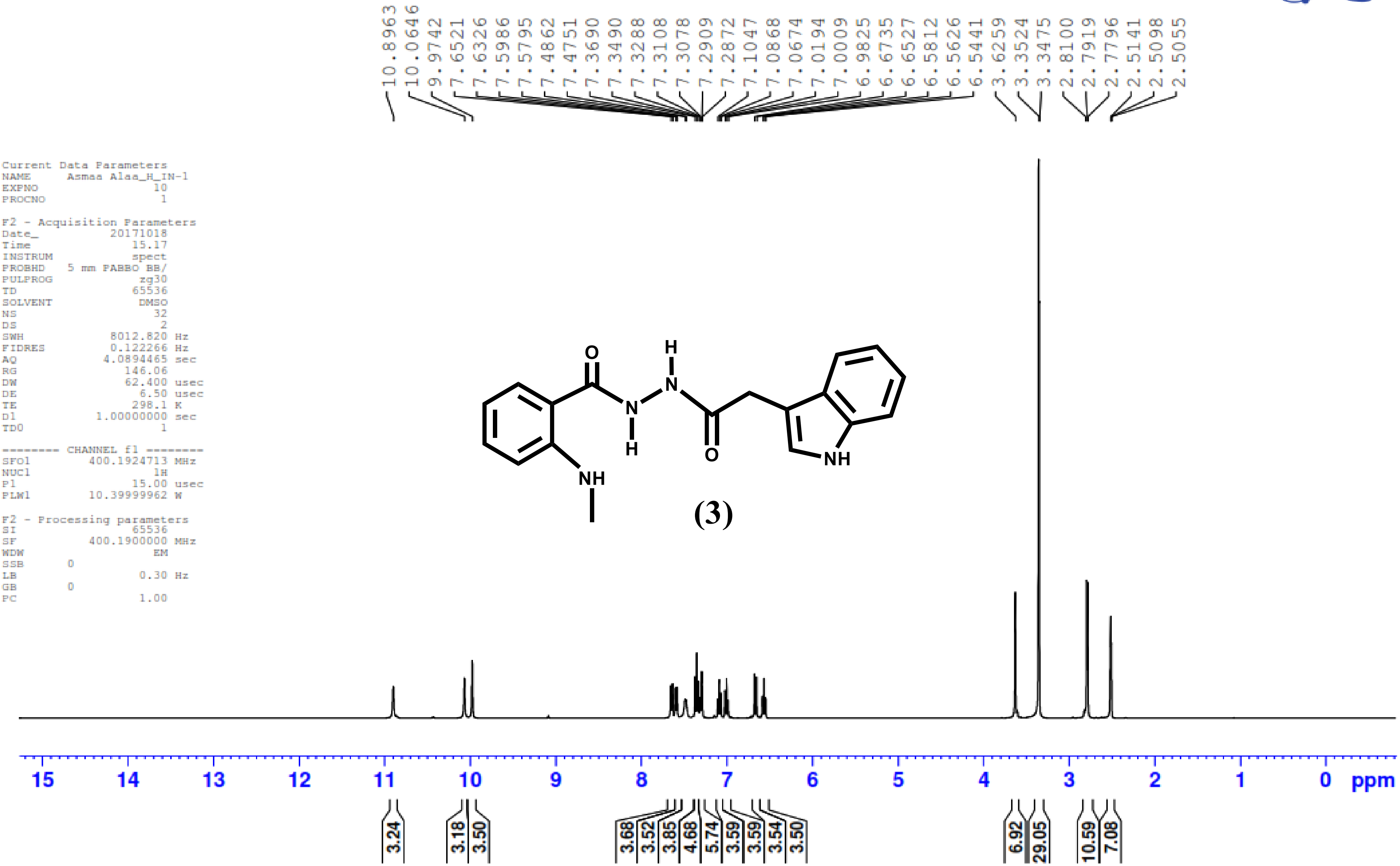

(3)

```
Current Data Parameters
NAME      Asmaa Alaa_H_IN-1
EXPNO                   10
PROCNO                   1

F2 - Acquisition Parameters
Date_             20171018
Time                 15.17
INSTRUM              spect
PROBHD   5 mm PABBO BB/
PULPROG               zg30
TD                   65536
SOLVENT               DMSO
NS                      32
DS                       2
SWH               8012.820 Hz
FIDRES            0.122266 Hz
AQ               4.0894465 sec
RG                  146.06
DW                  62.400 usec
DE                    6.50 usec
TE                   298.1 K
D1              1.00000000 sec
TD0                      1

======== CHANNEL f1 ========
SFO1          400.1924713 MHz
NUC1                    1H
P1                   15.00 usec
PLW1           10.39999962 W

F2 - Processing parameters
SI                   65536
SF            400.1900000 MHz
WDW                     EM
SSB       0
LB                    0.30 Hz
GB        0
PC                    1.00
```

Asmaa Alaa_H_IN-1_D2O

Microanalytical Unit - FOPCU - NMR laboratory
www.pharma.cu.edu.eg dir-mau.fopcu@pharma.cu.edu.eg

```
Current Data Parameters
NAME      Asmaa Alaa_H_IN-1_D2O
EXPNO                  10
PROCNO                  1

F2 - Acquisition Parameters
Date_           20171019
Time               11.36
INSTRUM            spect
PROBHD   5 mm PABBO BB/
PULPROG             zg30
TD                 65536
SOLVENT             DMSO
NS                    32
DS                     2
SWH             8012.820 Hz
FIDRES          0.122266 Hz
AQ             4.0894465 sec
RG                129.43
DW                62.400 usec
DE                  6.50 usec
TE                 298.0 K
D1            1.00000000 sec
TD0                    1

======== CHANNEL f1 ========
SFO1         400.1924713 MHz
NUC1                  1H
P1                 15.00 usec
PLW1         10.39999962 W

F2 - Processing parameters
SI                 65536
SF           400.1900000 MHz
WDW                   EM
SSB      0
LB                  0.30 Hz
GB       0
PC                  1.00
```

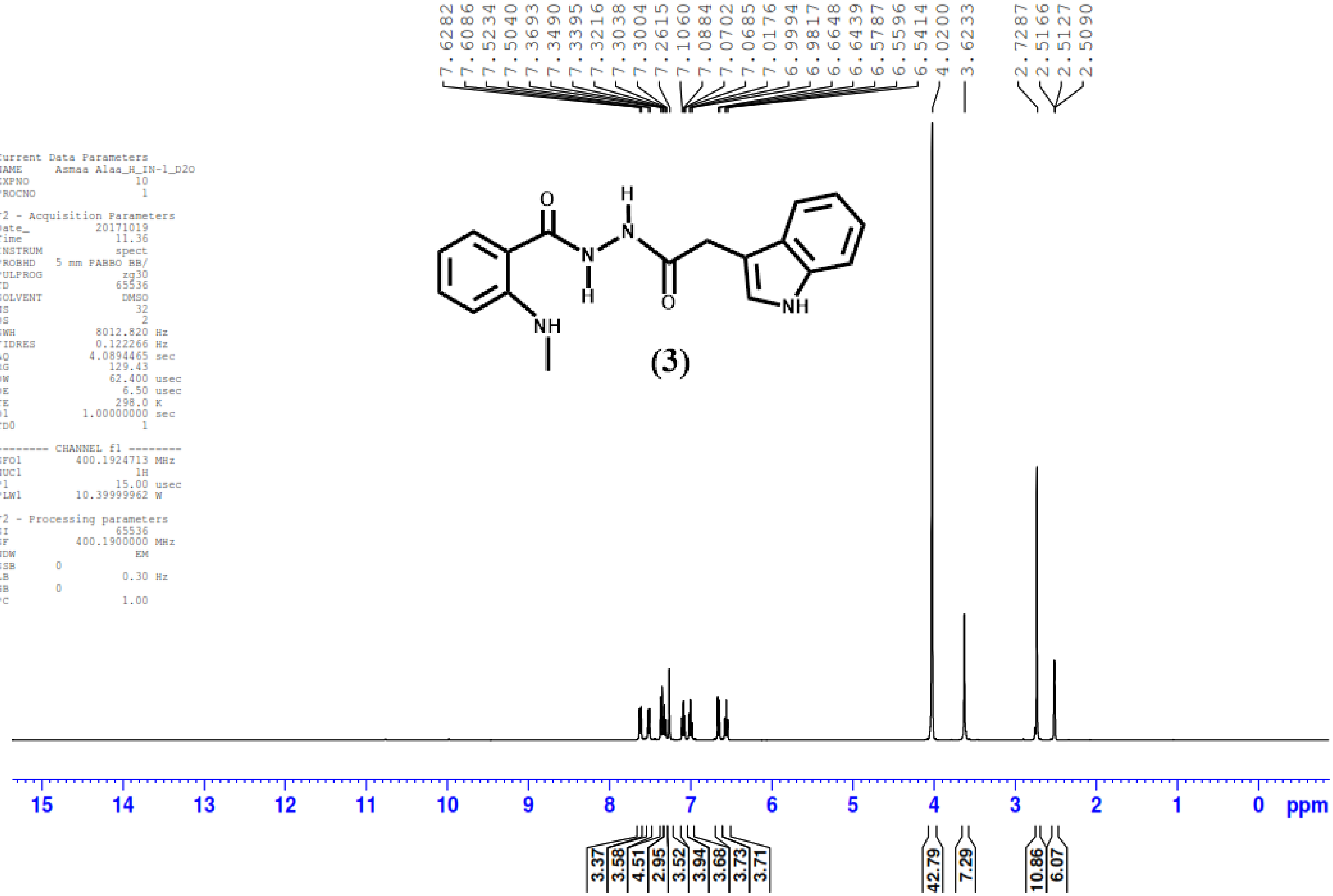

(3)

Asmaa Alaa_H_B-S30

Microanalytical Unit - FOPCU - NMR laboratory
www.pharma.cu.edu.eg dir-mau.fopcu@pharma.cu.edu.eg

BRUKER

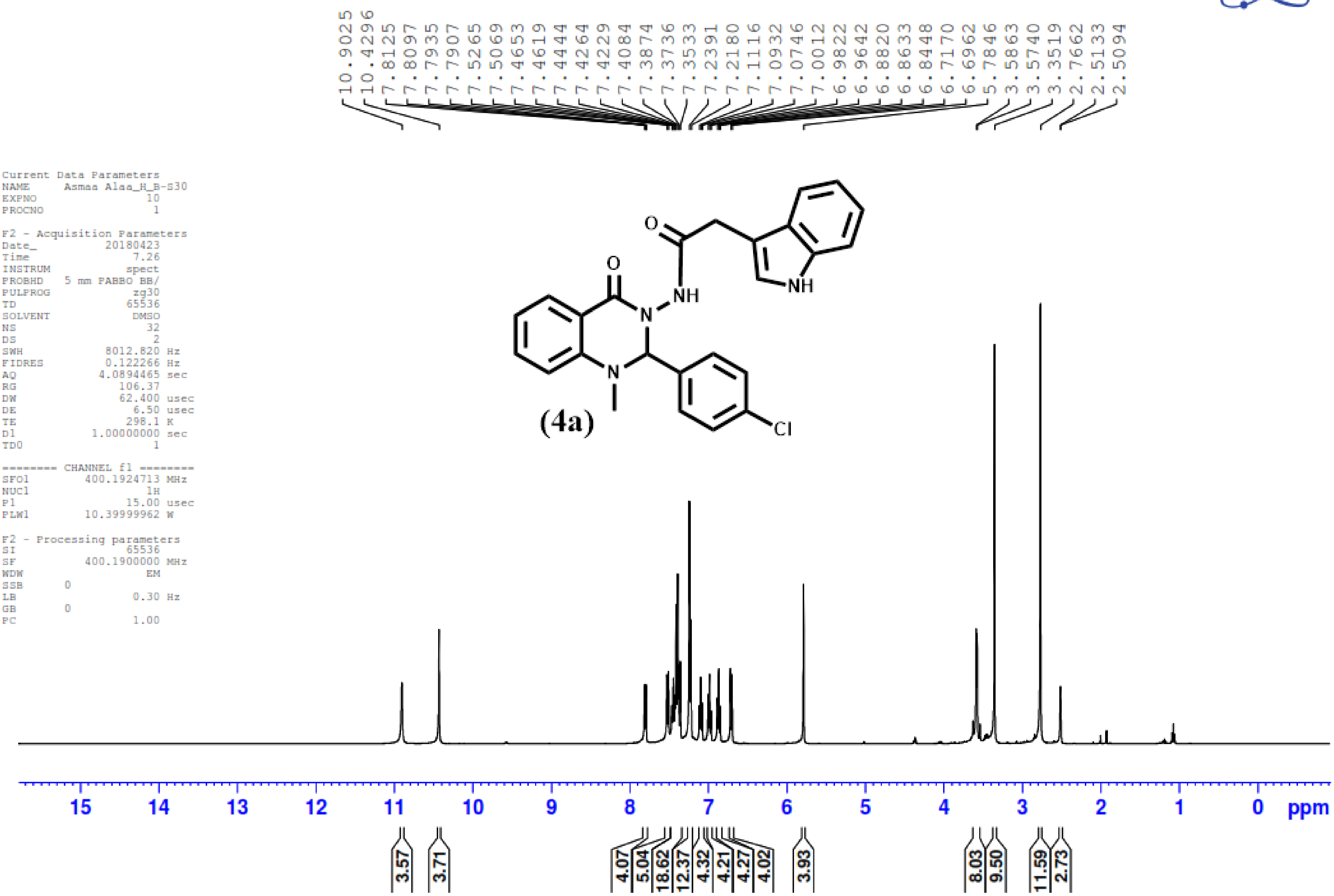

(4a)

```
Current Data Parameters
NAME     Asmaa Alaa_H_B-S30
EXPNO              10
PROCNO              1

F2 - Acquisition Parameters
Date_          20180423
Time               7.26
INSTRUM           spect
PROBHD   5 mm PABBO BB/
PULPROG            zg30
TD                65536
SOLVENT            DMSO
NS                   32
DS                    2
SWH            8012.820 Hz
FIDRES         0.122266 Hz
AQ            4.0894465 sec
RG               106.37
DW               62.400 usec
DE                 6.50 usec
TE                298.1 K
D1           1.00000000 sec
TD0                   1

======== CHANNEL f1 ========
SFO1        400.1924713 MHz
NUC1                 1H
P1                15.00 usec
PLW1        10.39999962 W

F2 - Processing parameters
SI                65536
SF          400.1900000 MHz
WDW                  EM
SSB       0
LB                 0.30 Hz
GB        0
PC                 1.00
```

Asmaa Sakr_C_B-S30

Microanalytical Unit - FOPCU - NMR laboratory
www.pharma.cu.edu.eg dir-mau.fopcu@pharma.cu.edu.eg

BRUKER

```
Current Data Parameters
NAME      Asmaa Sakr_C_B-S30
EXPNO                     10
PROCNO                     1

F2 - Acquisition Parameters
Date_               20180510
Time                    2.31
INSTRUM                spect
PROBHD    5 mm PABBO BB/
PULPROG              zgpg30
TD                     65536
SOLVENT                 DMSO
NS                      1200
DS                         4
SWH              24038.461 Hz
FIDRES            0.366798 Hz
AQ               1.3631488 sec
RG                    202.37
DW                    20.800 usec
DE                      6.50 usec
TE                     298.0 K
D1               2.00000000 sec
D11              0.03000000 sec
TD0                        1

======== CHANNEL f1 ========
SFO1          100.6379178 MHz
NUC1                   13C
P1                    10.00 usec
PLW1         45.00000000 W

======== CHANNEL f2 ========
SFO2          400.1916008 MHz
NUC2                    1H
CPDPRG[2            waltz16
```

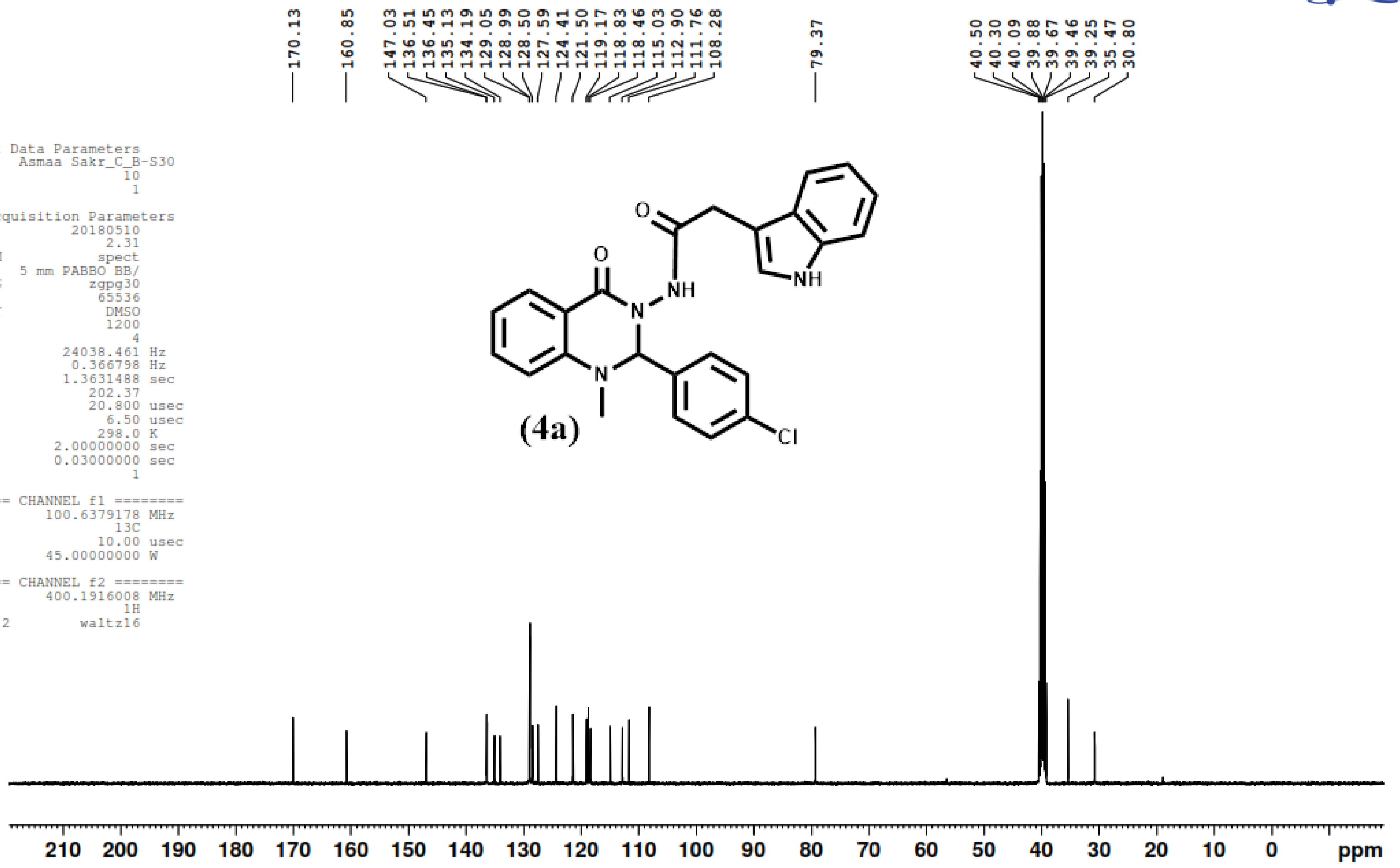

(4a)

Asmaa Alaa_H_B-S11

Microanalytical Unit - FOPCU - NMR laboratory
www.pharma.cu.edu.eg dir-mau.fopcu@pharma.cu.edu.eg

BRUKER

```
Current Data Parameters
NAME      Asmaa Alaa_H_B-S11
EXPNO                   10
PROCNO                   1

F2 - Acquisition Parameters
Date_             20171126
Time                 14.42
INSTRUM              spect
PROBHD   5 mm PABBO BB/
PULPROG               zg30
TD                   65536
SOLVENT               DMSO
NS                      32
DS                       2
SWH               8012.820 Hz
FIDRES            0.122266 Hz
AQ               4.0894465 sec
RG                  146.06
DW                  62.400 usec
DE                    6.50 usec
TE                   298.1 K
D1              1.00000000 sec
TD0                      1

======== CHANNEL f1 ========
SFO1         400.1924713 MHz
NUC1                    1H
P1                   15.00 usec
PLW1           10.39999962 W

F2 - Processing parameters
SI                   65536
SF             400.1900000 MHz
WDW                     EM
SSB       0
LB                    0.30 Hz
GB        0
PC                    1.00
```

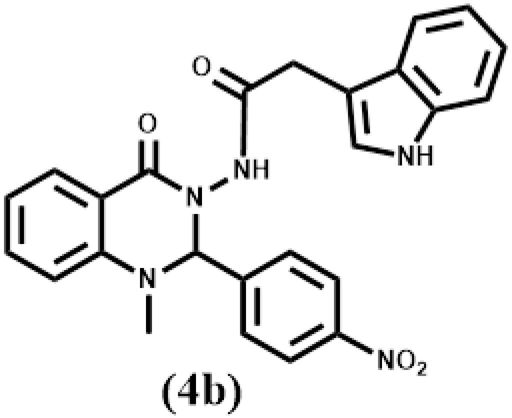

(4b)

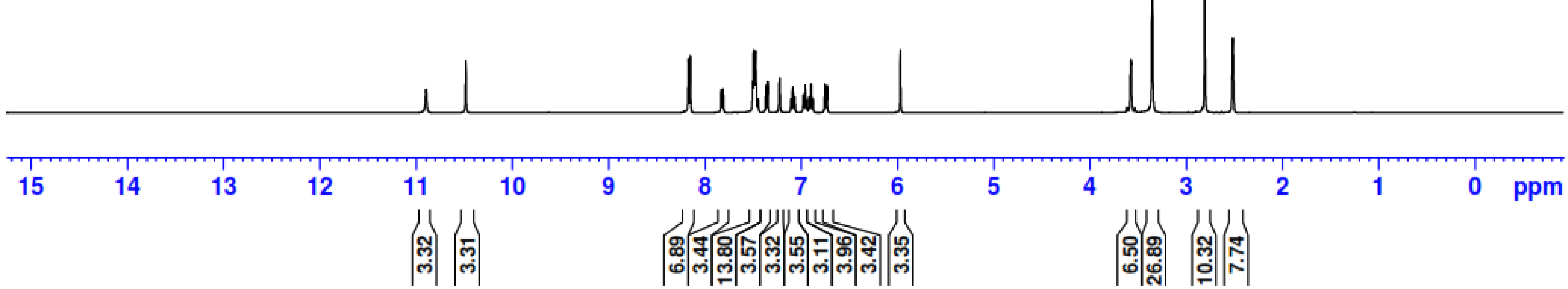

Asmaa Alaa_H_B-S11_D2O

Microanalytical Unit - FOPCU - NMR laboratory
www.pharma.cu.edu.eg dir-mau.fopcu@pharma.cu.edu.eg

```
Current Data Parameters
NAME      Asmaa Alaa_H_B-S11_D2O
EXPNO               10
PROCNO               1

F2 - Acquisition Parameters
Date_         20171210
Time             14.18
INSTRUM          spect
PROBHD   5 mm PABBO BB/
PULPROG           zg30
TD               65536
SOLVENT           DMSO
NS                  32
DS                   2
SWH           8012.820 Hz
FIDRES        0.122266 Hz
AQ           4.0894465 sec
RG              106.37
DW              62.400 usec
DE                6.50 usec
TE               298.0 K
D1          1.00000000 sec
TD0                  1

======== CHANNEL f1 ========
SFO1       400.1924713 MHz
NUC1                1H
P1               15.00 usec
PLW1       10.39999962 W

F2 - Processing parameters
SI               65536
SF         400.1900000 MHz
WDW                 EM
SSB      0
LB                0.30 Hz
GB       0
PC                1.00
```

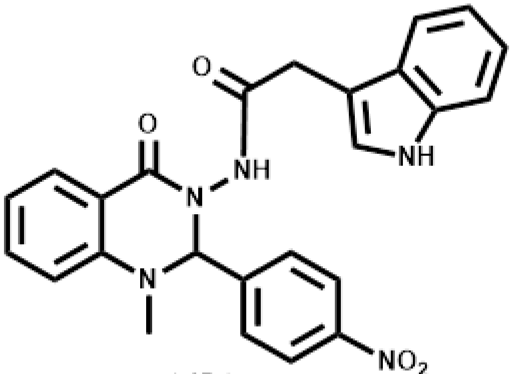

(4b)

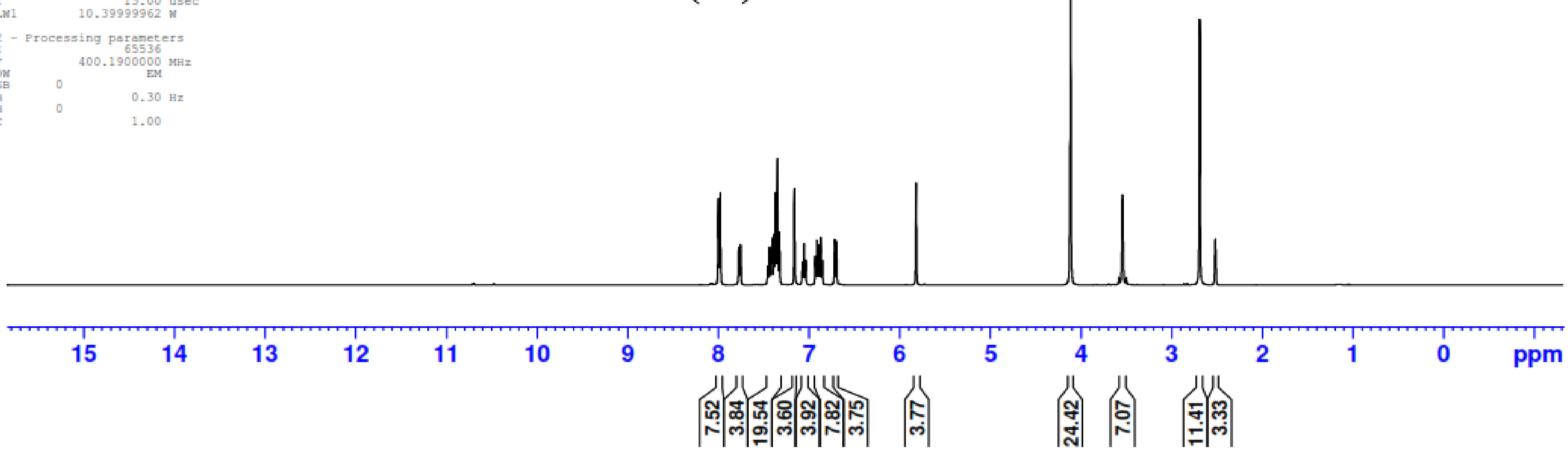

Asmaa Alaa_C_B-S11

Microanalytical Unit - FOPCU - NMR laboratory
www.pharma.cu.edu.eg dir-mau.fopcu@pharma.cu.edu.eg

```
Current Data Parameters
NAME      Asmaa Alaa_C_B-S11
EXPNO                   10
PROCNO                   1

F2 - Acquisition Parameters
Date_             20171207
Time                 16.47
INSTRUM              spect
PROBHD   5 mm PABBO BB/
PULPROG            zgpg30
TD                   65536
SOLVENT               DMSO
NS                    1200
DS                       4
SWH              24038.461 Hz
FIDRES            0.366798 Hz
AQ               1.3631488 sec
RG                  202.37
DW                  20.800 usec
DE                    6.50 usec
TE                   298.1 K
D1              2.00000000 sec
D11             0.03000000 sec
TD0                      1

======== CHANNEL f1 ========
SFO1           100.6379178 MHz
NUC1                   13C
P1                   10.00 usec
PLW1           45.00000000 W

======== CHANNEL f2 ========
SFO2           400.1916008 MHz
NUC2                    1H
CPDPRG[2           waltz16
```

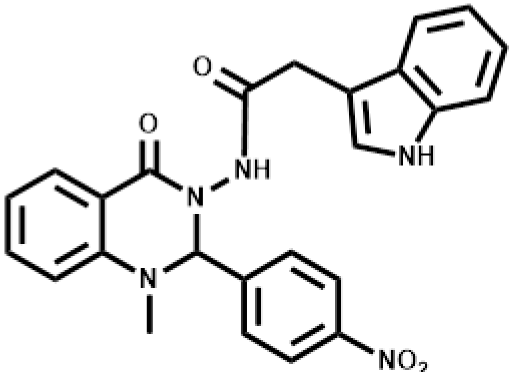

(4b)

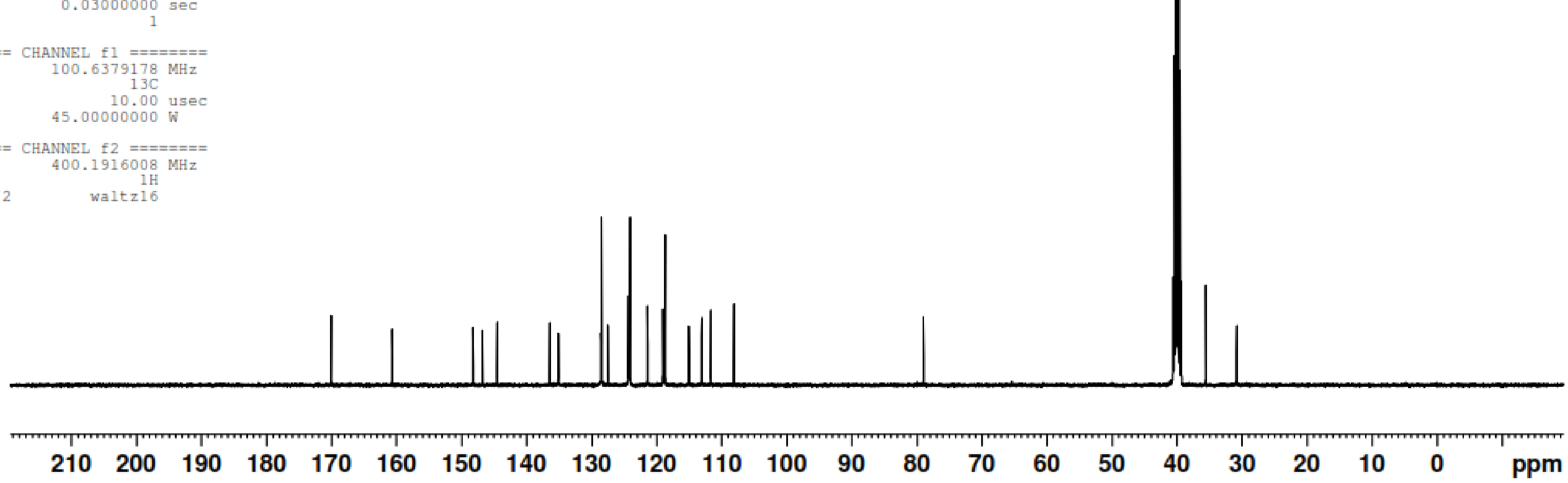

Asmaa Alaa_H_B-S31

Microanalytical Unit - FOPCU - NMR laboratory
www.pharma.cu.edu.eg dir-mau.fopcu@pharma.cu.edu.eg

BRUKER

```
Current Data Parameters
NAME      Asmaa Alaa_H_B-S31
EXPNO               10
PROCNO               1

F2 - Acquisition Parameters
Date_          20180423
Time               7.21
INSTRUM           spect
PROBHD   5 mm PABBO BB/
PULPROG            zg30
TD                65536
SOLVENT            DMSO
NS                   32
DS                    2
SWH            8012.820 Hz
FIDRES         0.122266 Hz
AQ            4.0894465 sec
RG                91.58
DW               62.400 usec
DE                 6.50 usec
TE                298.0 K
D1           1.00000000 sec
TD0                   1

======== CHANNEL f1 ========
SFO1        400.1924713 MHz
NUC1                 1H
P1                15.00 usec
PLW1        10.39999962 W

F2 - Processing parameters
SI                65536
SF          400.1900000 MHz
WDW                  EM
SSB       0
LB                 0.30 Hz
GB        0
PC                 1.00
```

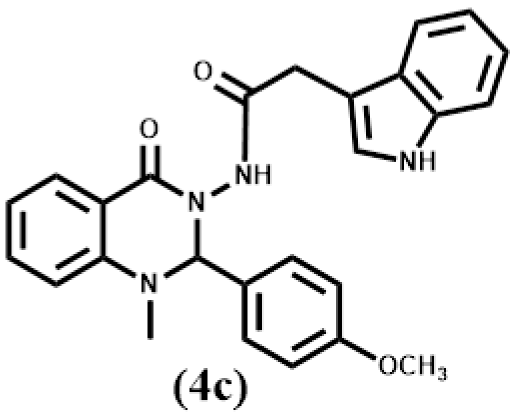

(4c)

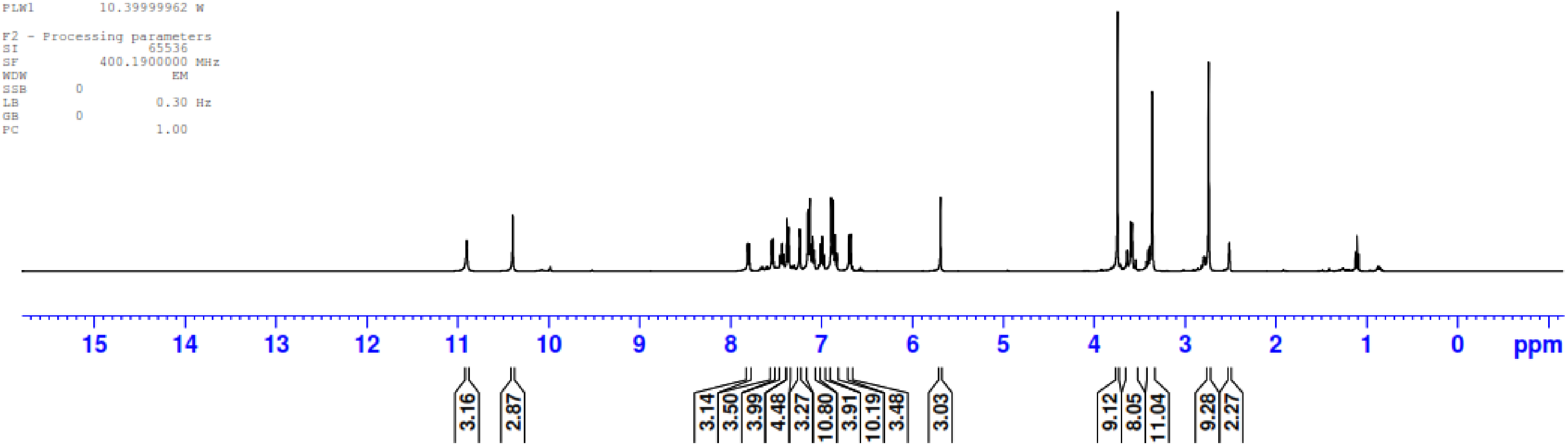

Asmaa Sakr_H_B-S31_D2O

Microanalytical Unit - FOPCU - NMR laboratory
www.pharma.cu.edu.eg dir-mau.fopcu@pharma.cu.edu.eg

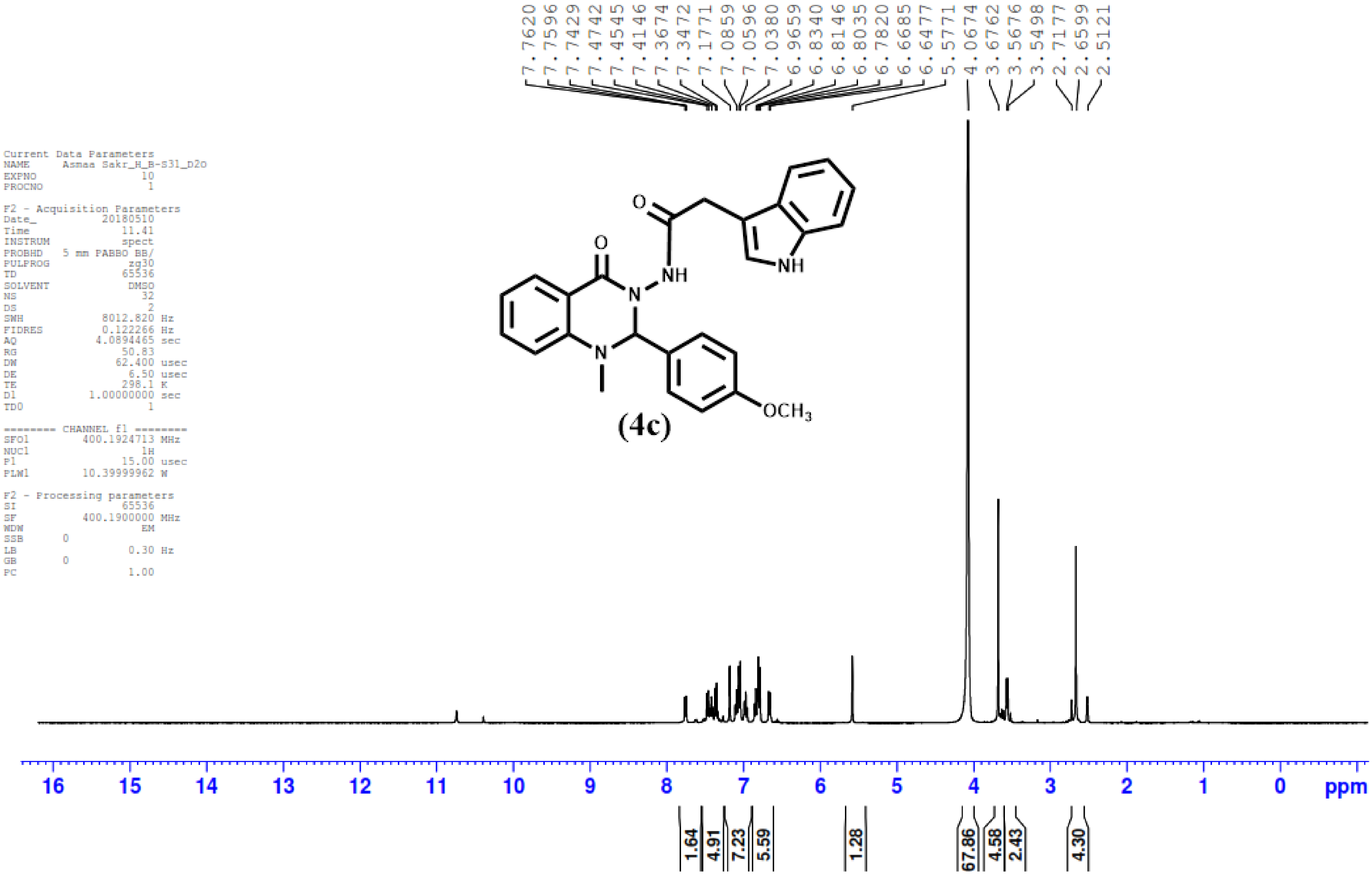

Asmaa Sakr_C_B-S31

Microanalytical Unit - FOPCU - NMR laboratory
www.pharma.cu.edu.eg dir-mau.fopcu@pharma.cu.edu.eg

BRUKER

```
Current Data Parameters
NAME      Asmaa Sakr_C_B-S31
EXPNO                   10
PROCNO                   1

F2 - Acquisition Parameters
Date_             20180510
Time                  1.20
INSTRUM              spect
PROBHD   5 mm PABBO BB/
PULPROG            zgpg30
TD                  65536
SOLVENT              DMSO
NS                   1200
DS                      4
SWH             24038.461 Hz
FIDRES           0.366798 Hz
AQ              1.3631488 sec
RG                 202.37
DW                 20.800 usec
DE                   6.50 usec
TE                  298.1 K
D1             2.00000000 sec
D11            0.03000000 sec
TD0                     1

======== CHANNEL f1 ========
SFO1          100.6379178 MHz
NUC1                  13C
P1                  10.00 usec
PLW1          45.00000000 W

======== CHANNEL f2 ========
SFO2          400.1916008 MHz
NUC2                   1H
CPDPRG[2          waltz16
```

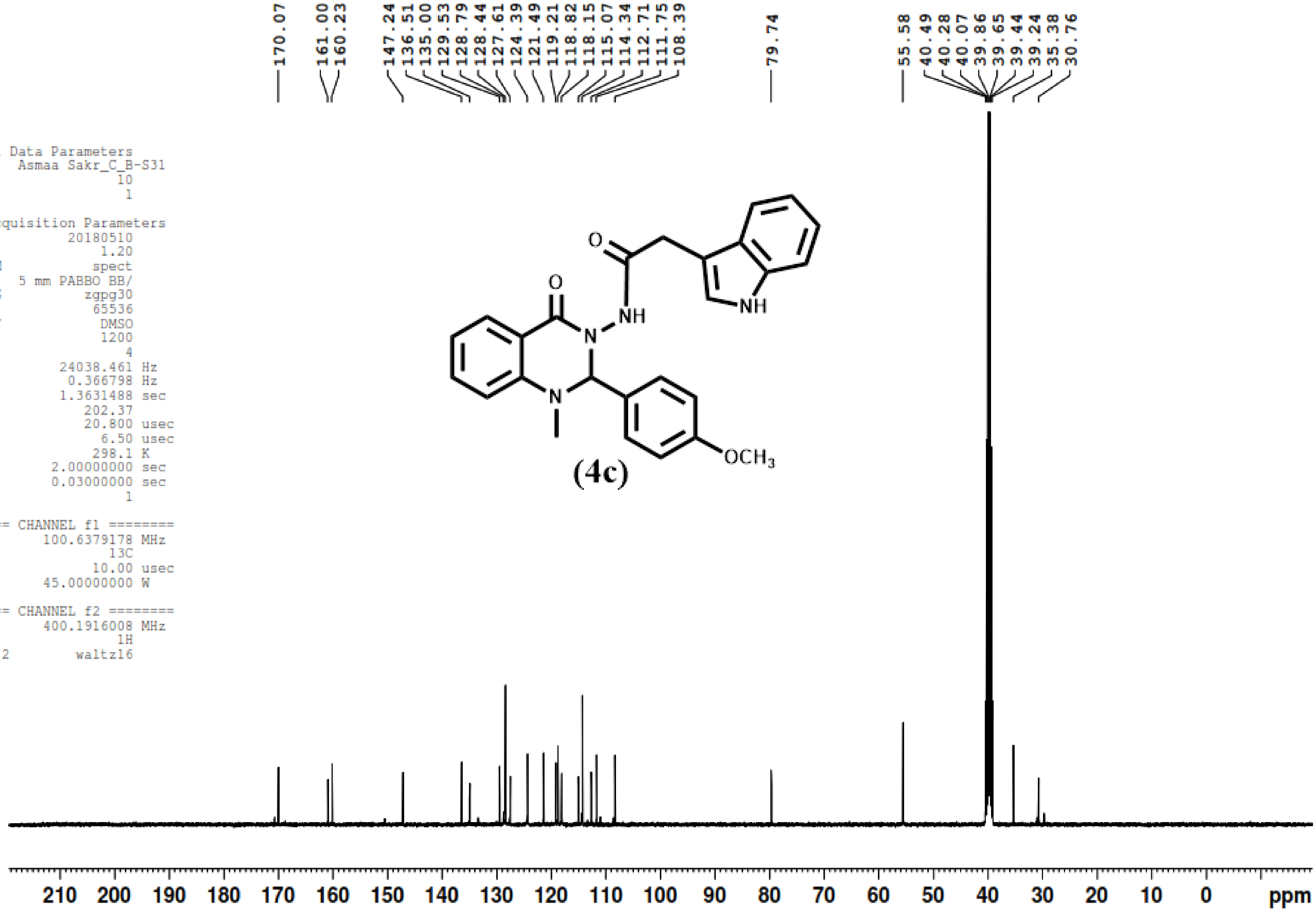

(4c)

Asmaa Alaa_H_IN2

Microanalytical Unit - FOPCU - NMR laboratory
www.pharma.cu.edu.eg dir-mau.fopcu@pharma.cu.edu.eg

BRUKER

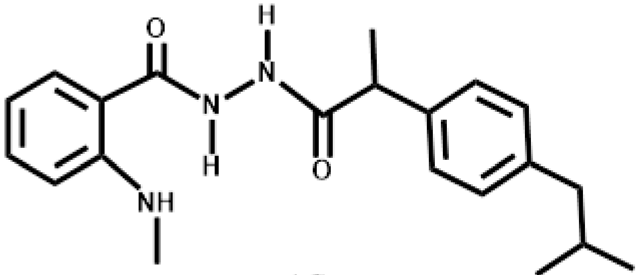

(6)

```
Current Data Parameters
NAME       Asmaa Alaa_H_IN2
EXPNO                    10
PROCNO                    1

F2 - Acquisition Parameters
Date_              20171107
Time                  14.42
INSTRUM               spect
PROBHD    5 mm PABBO BB/
PULPROG                zg30
TD                    65536
SOLVENT                DMSO
NS                       32
DS                        2
SWH                8012.820 Hz
FIDRES             0.122266 Hz
AQ                4.0894465 sec
RG                   114.95
DW                   62.400 usec
DE                     6.50 usec
TE                    298.0 K
D1               1.00000000 sec
TD0                       1

======== CHANNEL f1 ========
SFO1            400.1924713 MHz
NUC1                     1H
P1                    15.00 usec
PLW1            10.39999962 W

F2 - Processing parameters
SI                    65536
SF              400.1900000 MHz
WDW                      EM
SSB       0
LB                     0.30 Hz
GB        0
PC                     1.00
```

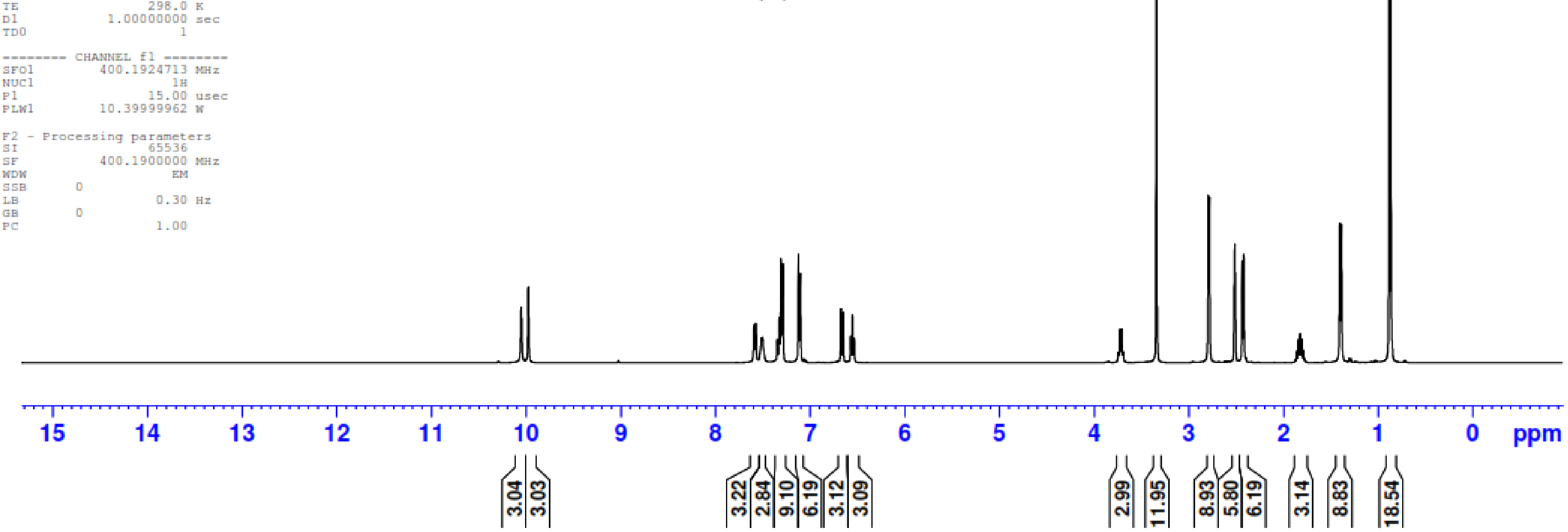

Asmaa Alaa_H_IN2_D2O

Microanalytical Unit - FOPCU - NMR laboratory
www.pharma.cu.edu.eg dir-mau.fopcu@pharma.cu.edu.eg

BRUKER

```
Current Data Parameters
NAME      Asmaa Alaa_H_IN2_D2O
EXPNO                   10
PROCNO                   1

F2 - Acquisition Parameters
Date_             20171108
Time                 12.33
INSTRUM              spect
PROBHD   5 mm PABBO BB/
PULPROG               zg30
TD                   65536
SOLVENT               DMSO
NS                      32
DS                       2
SWH              8012.820 Hz
FIDRES           0.122266 Hz
AQ              4.0894465 sec
RG                 129.43
DW                 62.400 usec
DE                   6.50 usec
TE                  298.0 K
D1            1.00000000 sec
TD0                      1

======== CHANNEL f1 ========
SFO1         400.1924713 MHz
NUC1                    1H
P1                   15.00 usec
PLW1           10.39999962 W

F2 - Processing parameters
SI                   65536
SF             400.1900000 MHz
WDW                     EM
SSB       0
LB                    0.30 Hz
GB        0
PC                    1.00
```

(6)

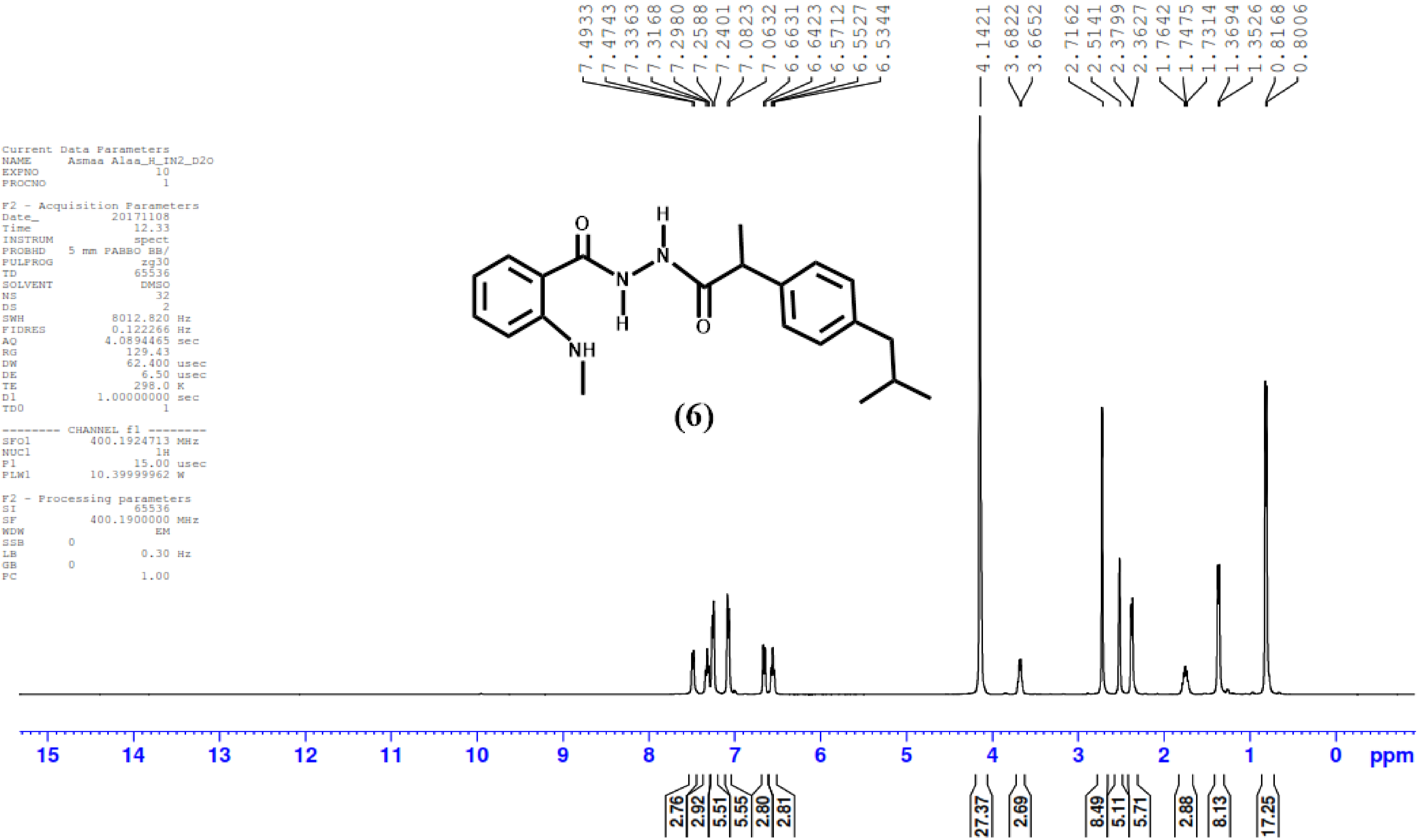

Asmaa Alaa_H_B-S17

Microanalytical Unit - FOPCU - NMR laboratory
www.pharma.cu.edu.eg dir-mau.fopcu@pharma.cu.edu.eg

BRUKER

```
Current Data Parameters
NAME       Asmaa Alaa_H_B-S17
EXPNO                   10
PROCNO                   1

F2 - Acquisition Parameters
Date_          20180423
Time               7.15
INSTRUM           spect
PROBHD   5 mm PABBO BB/
PULPROG            zg30
TD                65536
SOLVENT            DMSO
NS                   32
DS                    2
SWH            8012.820 Hz
FIDRES         0.122266 Hz
AQ            4.0894465 sec
RG                84.65
DW               62.400 usec
DE                 6.50 usec
TE                298.0 K
D1           1.00000000 sec
TD0                   1

======== CHANNEL f1 ========
SFO1        400.1924713 MHz
NUC1                 1H
P1                15.00 usec
PLW1        10.39999962 W

F2 - Processing parameters
SI                65536
SF          400.1900000 MHz
WDW                  EM
SSB       0
LB                 0.30 Hz
GB        0
PC                 1.00
```

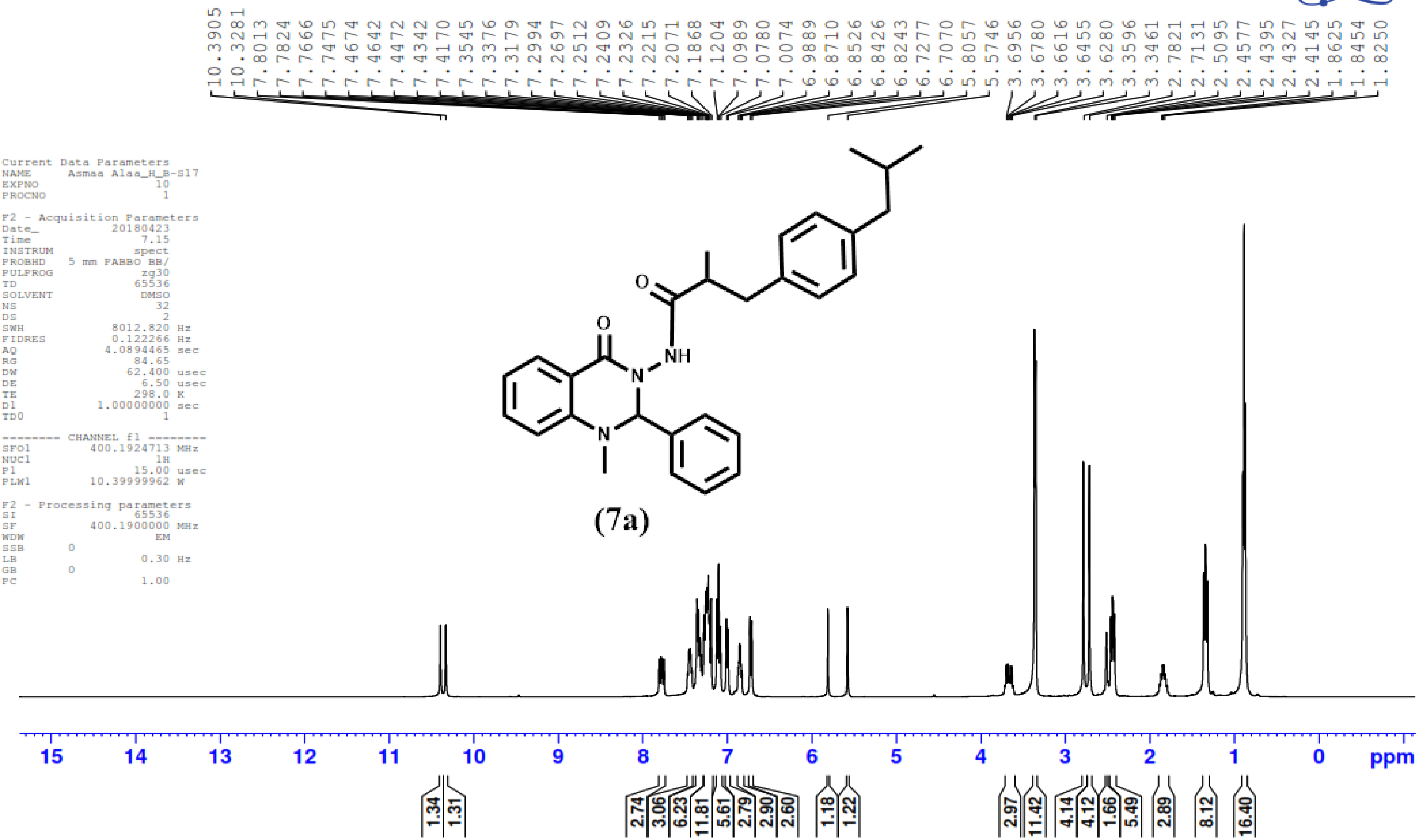

Asmaa Sakr_C_B-S17

Microanalytical Unit - FOPCU - NMR laboratory
www.pharma.cu.edu.eg dir-mau.fopcu@pharma.cu.edu.eg

BRUKER

```
Current Data Parameters
NAME      Asmaa Sakr_C_B-S17
EXPNO                   10
PROCNO                   1

F2 - Acquisition Parameters
Date_            20180510
Time                 0.09
INSTRUM             spect
PROBHD   5 mm PABBO BB/
PULPROG           zgpg30
TD                 65536
SOLVENT             DMSO
NS                  1200
DS                     4
SWH            24038.461 Hz
FIDRES          0.366798 Hz
AQ             1.3631488 sec
RG                202.37
DW                20.800 usec
DE                  6.50 usec
TE                 298.0 K
D1            2.00000000 sec
D11           0.03000000 sec
TD0                    1

======== CHANNEL f1 ========
SFO1         100.6379178 MHz
NUC1                 13C
P1                 10.00 usec
PLW1         45.00000000 W

======== CHANNEL f2 ========
SFO2         400.1916008 MHz
NUC2                  1H
CPDPRG[2          waltz16
```

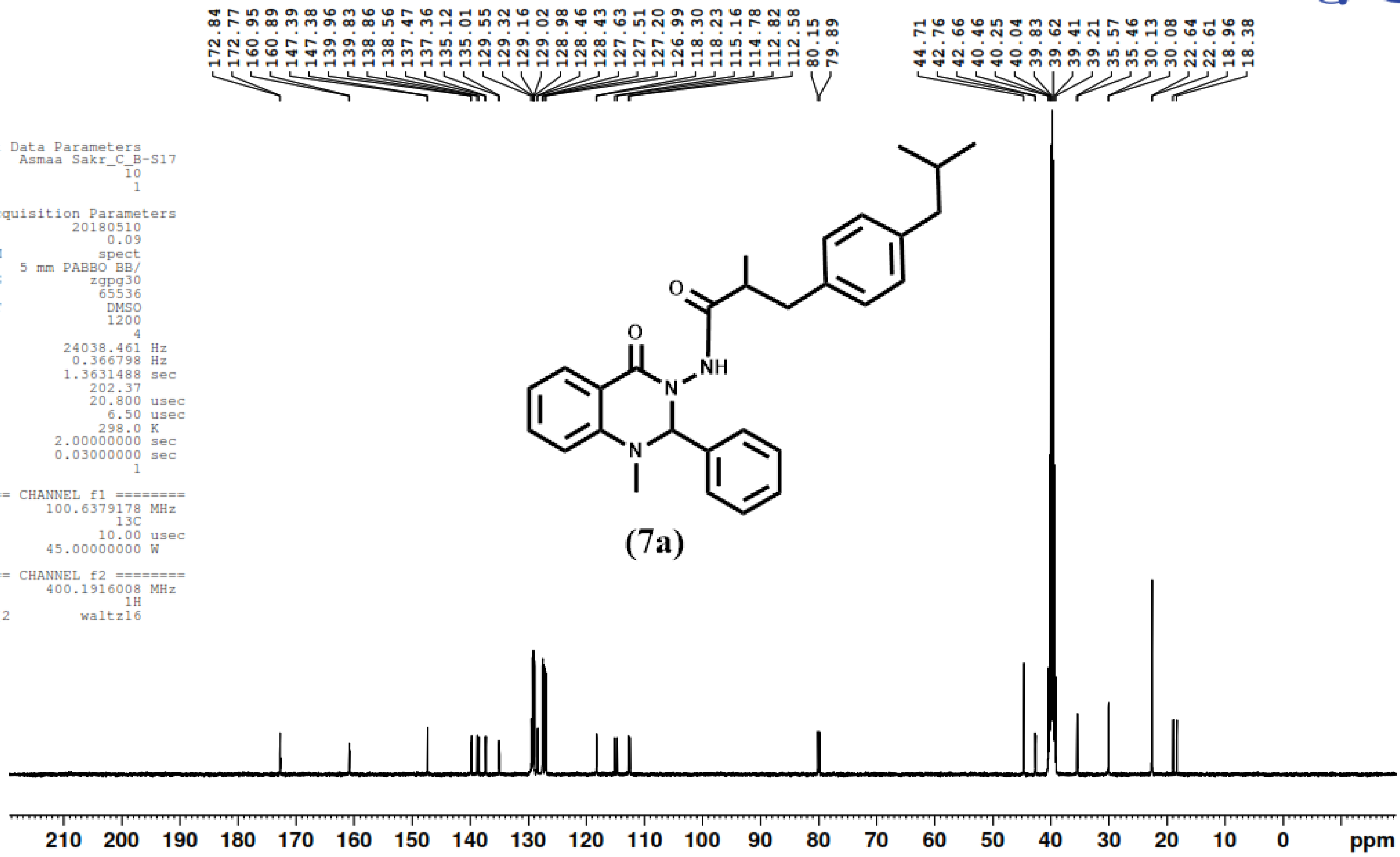

(7a)

Asmaa Alaa_H_B-S13

Microanalytical Unit - FOPCU - NMR laboratory
www.pharma.cu.edu.eg dir-mau.fopcu@pharma.cu.edu.eg

BRUKER

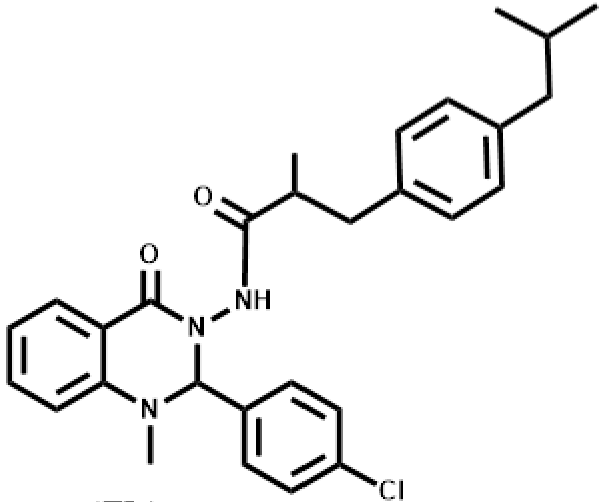

(7b)

```
Current Data Parameters
NAME     Asmaa Alaa_H_B-S13
EXPNO              10
PROCNO              1

F2 - Acquisition Parameters
Date_          20171207
Time              14.42
INSTRUM           spect
PROBHD   5 mm PABBO BB/
PULPROG            zg30
TD                65536
SOLVENT            DMSO
NS                   32
DS                    2
SWH           8012.820 Hz
FIDRES         0.122266 Hz
AQ            4.0894465 sec
RG                91.58
DW               62.400 usec
DE                 6.50 usec
TE                298.1 K
D1           1.00000000 sec
TD0                   1

======== CHANNEL f1 ========
SFO1        400.1924713 MHz
NUC1                 1H
P1                15.00 usec
PLW1        10.39999962 W

F2 - Processing parameters
SI                65536
SF          400.1900000 MHz
WDW                  EM
SSB      0
LB                 0.30 Hz
GB       0
PC                 1.00
```

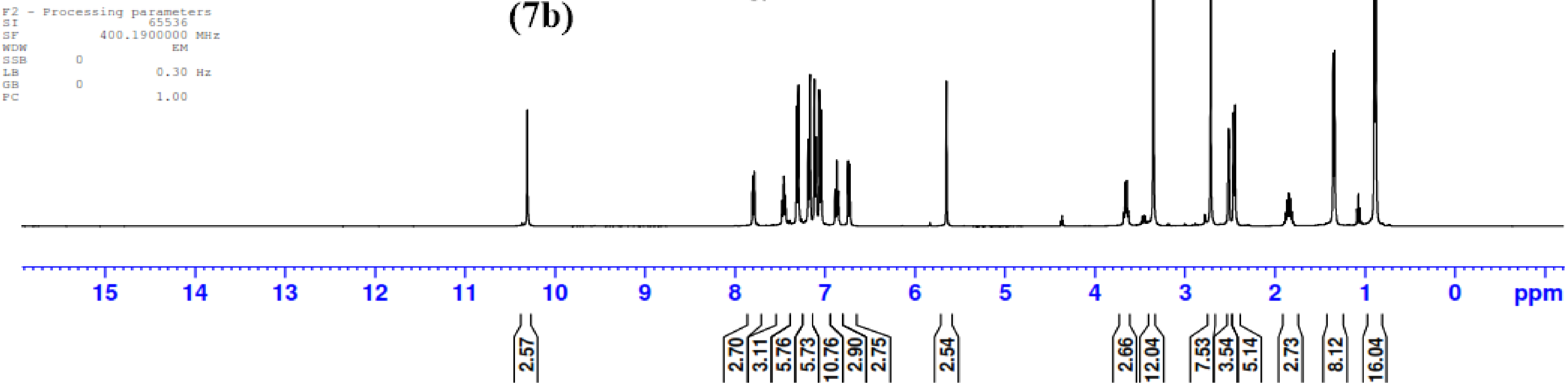

Asmaa Alaa_H_BS13_D2O

Microanalytical Unit - FOPCU - NMR laboratory
www.pharma.cu.edu.eg dir-mau.fopcu@pharma.cu.edu.eg

BRUKER

```
Current Data Parameters
NAME       Asmaa Alaa_H_BS13_D2O
EXPNO                 10
PROCNO                 1

F2 - Acquisition Parameters
Date_           20180102
Time               15.33
INSTRUM            spect
PROBHD   5 mm PABBO BB/
PULPROG             zg30
TD                 65536
SOLVENT             DMSO
NS                    32
DS                     2
SWH             8012.820 Hz
FIDRES          0.122266 Hz
AQ             4.0894465 sec
RG                 84.65
DW                62.400 usec
DE                  6.50 usec
TE                 298.0 K
D1            1.00000000 sec
TD0                    1

======== CHANNEL f1 ========
SFO1         400.1924713 MHz
NUC1                  1H
P1                 15.00 usec
PLW1         10.39999962 W

F2 - Processing parameters
SI                 65536
SF           400.1900000 MHz
WDW                   EM
SSB        0
LB                  0.30 Hz
GB         0
PC                  1.00
```

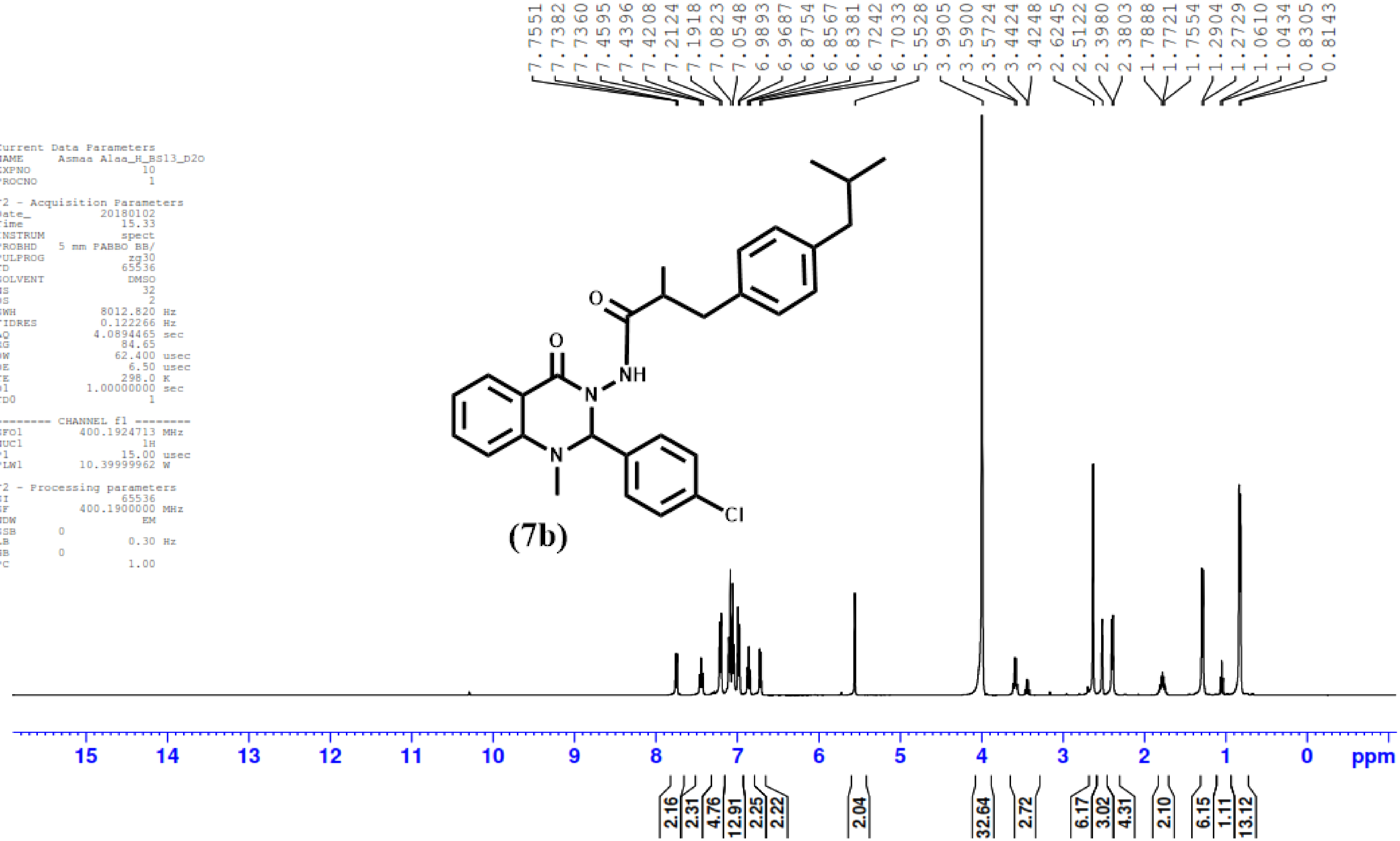

(7b)

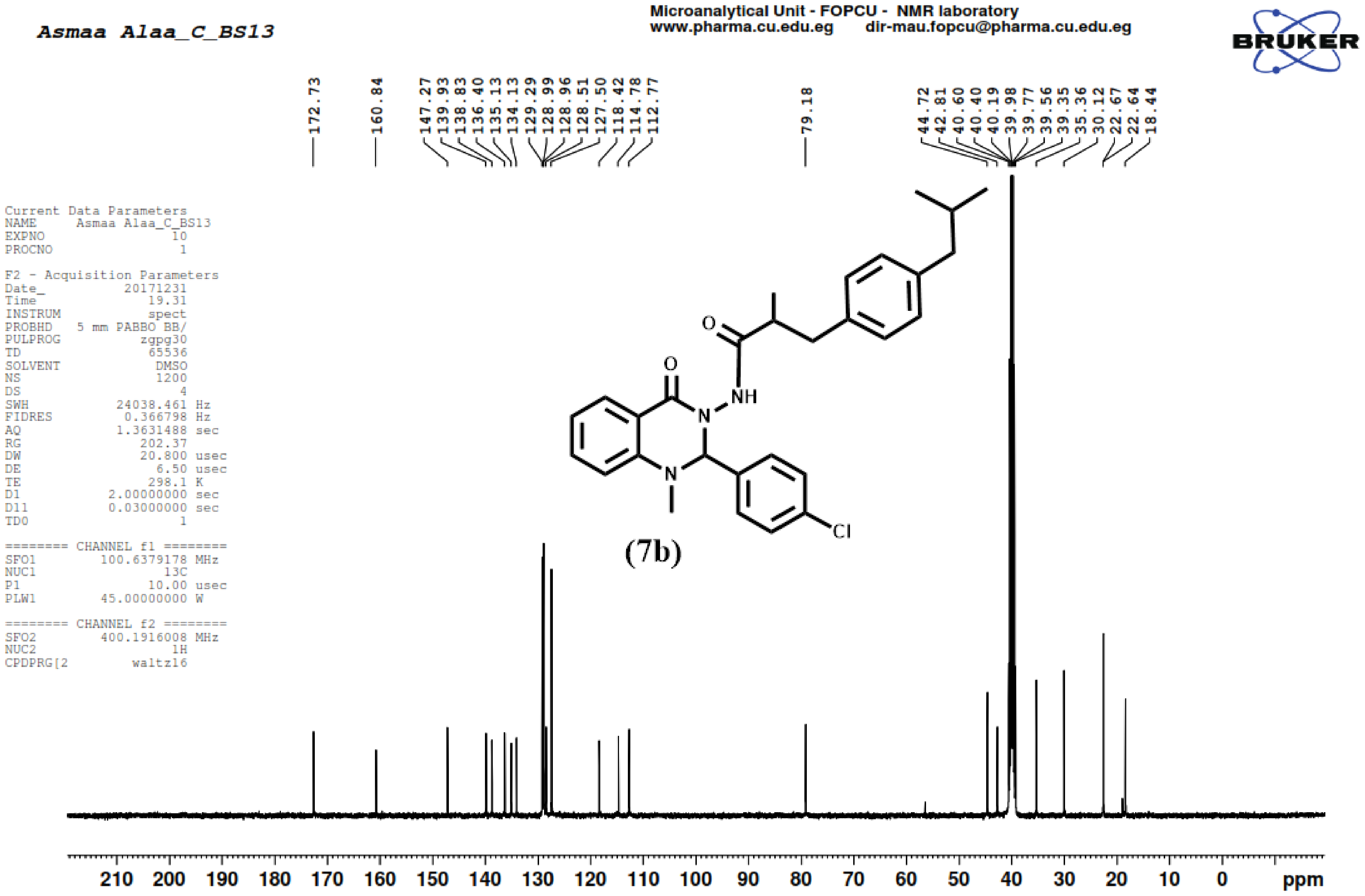
Asmaa Alaa_C_BS13
Microanalytical Unit - FOPCU - NMR laboratory
www.pharma.cu.edu.eg dir-mau.fopcu@pharma.cu.edu.eg
BRUKER
172.73
160.84
147.27
139.93
138.83
136.40
135.13
134.13
129.29
128.99
128.96
128.51
127.50
118.42
114.78
112.77
79.18
44.72
42.81
40.60
40.40
40.19
39.98
39.77
39.56
39.35
35.36
30.12
22.67
22.64
18.44
Current Data Parameters
NAME Asmaa Alaa_C_BS13
EXPNO 10
PROCNO 1
F2 - Acquisition Parameters
Date_ 20171231
Time 19.31
INSTRUM spect
PROBHD 5 mm PABBO BB/
PULPROG zgpg30
TD 65536
SOLVENT DMSO
NS 1200
DS 4
SWH 24038.461 Hz
FIDRES 0.366798 Hz
AQ 1.3631488 sec
RG 202.37
DW 20.800 usec
DE 6.50 usec
TE 298.1 K
D1 2.00000000 sec
D11 0.03000000 sec
TD0 1
======== CHANNEL f1 ========
SFO1 100.6379178 MHz
NUC1 13C
P1 10.00 usec
PLW1 45.00000000 W
======== CHANNEL f2 ========
SFO2 400.1916008 MHz
NUC2 1H
CPDPRG[2 waltz16
O
O
NH
N
N
Cl
(7b)
210 200 190 180 170 160 150 140 130 120 110 100 90 80 70 60 50 40 30 20 10 0 ppm

Asmaa Alaa_H_B-S14

Microanalytical Unit - FOPCU - NMR laboratory
www.pharma.cu.edu.eg dir-mau.fopcu@pharma.cu.edu.eg

BRUKER

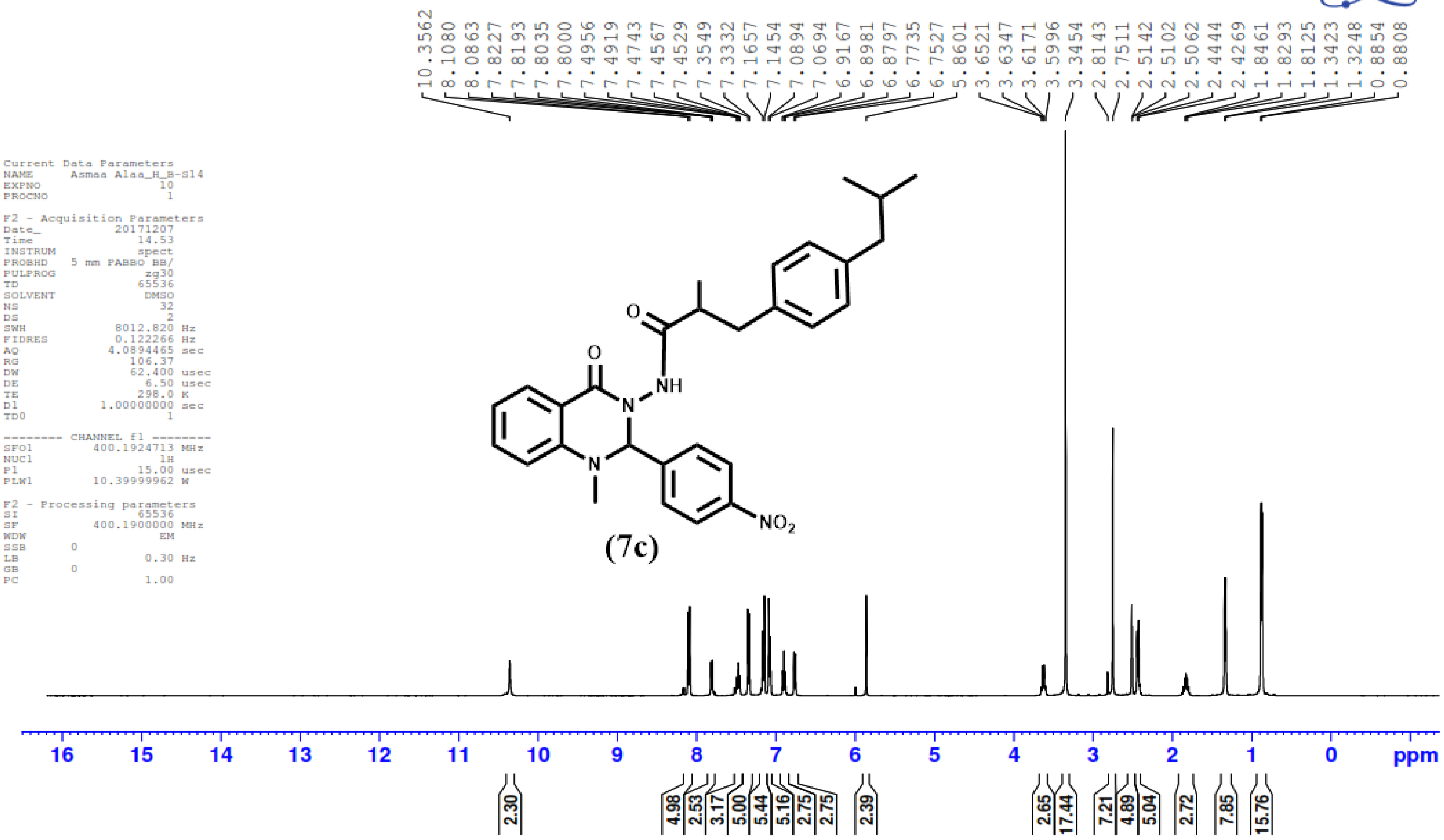

Asmaa Alaa_H_BS14_D2O

Microanalytical Unit - FOPCU - NMR laboratory
www.pharma.cu.edu.eg dir-mau.fopcu@pharma.cu.edu.eg

```
Current Data Parameters
NAME      Asmaa Alaa_H_BS14_D2O-
EXPNO               10
PROCNO               1

F2 - Acquisition Parameters
Date_         20180103
Time             12.43
INSTRUM          spect
PROBHD   5 mm PABBO BB/
PULPROG           zg30
TD               65536
SOLVENT           DMSO
NS                  32
DS                   2
SWH           8012.820 Hz
FIDRES        0.122266 Hz
AQ           4.0894465 sec
RG               32.12
DW              62.400 usec
DE                6.50 usec
TE               298.1 K
D1          1.00000000 sec
TD0                  1

======== CHANNEL f1 ========
SFO1       400.1924713 MHz
NUC1                1H
P1               15.00 usec
PLW1       10.39999962 W

F2 - Processing parameters
SI               65536
SF         400.1900000 MHz
WDW                 EM
SSB      0
LB                0.30 Hz
GB       0
PC                1.00
```

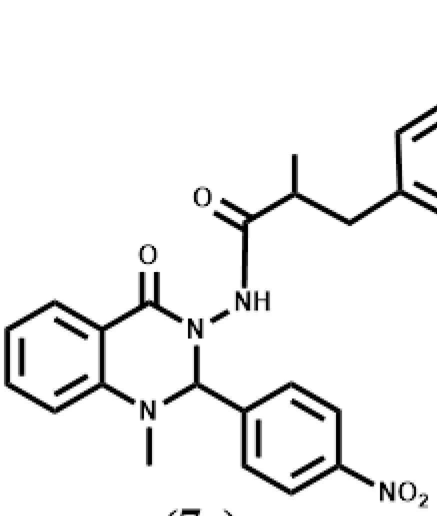

(7c)

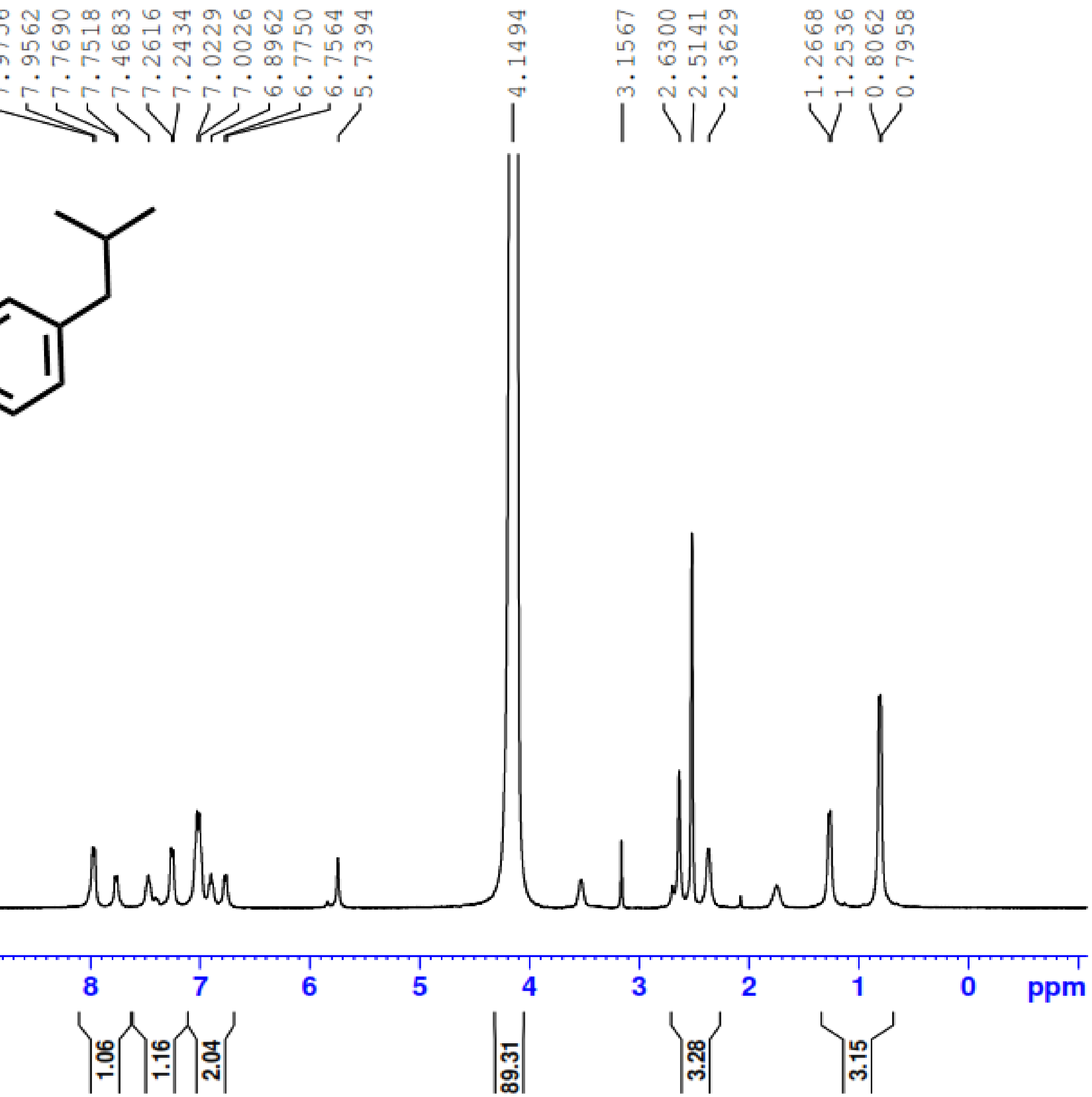

Asmaa Alaa_C_BS14

Microanalytical Unit - FOPCU - NMR laboratory
www.pharma.cu.edu.eg dir-mau.fopcu@pharma.cu.edu.eg

BRUKER

```
Current Data Parameters
NAME       Asmaa Alaa_C_BS14
EXPNO                    10
PROCNO                    1

F2 - Acquisition Parameters
Date_              20171231
Time                  21.54
INSTRUM               spect
PROBHD    5 mm PABBO BB/
PULPROG             zgpg30
TD                   65536
SOLVENT               DMSO
NS                    1200
DS                       4
SWH             24038.461 Hz
FIDRES           0.366798 Hz
AQ              1.3631488 sec
RG                 202.37
DW                 20.800 usec
DE                   6.50 usec
TE                  298.1 K
D1             2.00000000 sec
D11            0.03000000 sec
TD0                     1

======== CHANNEL f1 ========
SFO1          100.6379178 MHz
NUC1                  13C
P1                  10.00 usec
PLW1          45.00000000 W

======== CHANNEL f2 ========
SFO2          400.1916008 MHz
NUC2                   1H
CPDPRG[2            waltz16
```

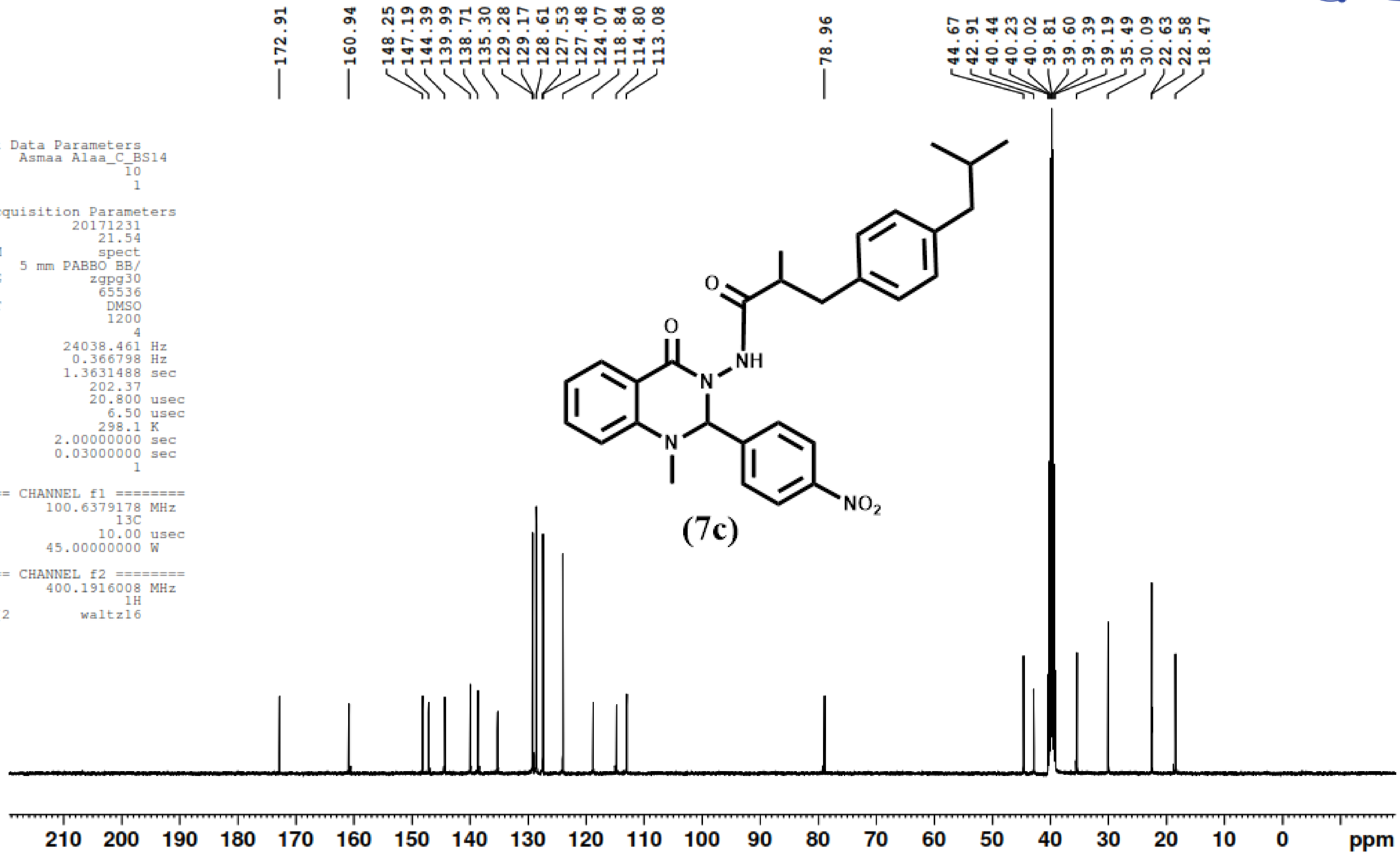

Asmaa Alaa_H_B-S15

Microanalytical Unit - FOPCU - NMR laboratory
www.pharma.cu.edu.eg dir-mau.fopcu@pharma.cu.edu.eg

```
Current Data Parameters
NAME       Asmaa Alaa_H_B-S15
EXPNO                      10
PROCNO                      1

F2 - Acquisition Parameters
Date_              20180102
Time                  18.04
INSTRUM               spect
PROBHD    5 mm PABBO BB/
PULPROG                zg30
TD                    65536
SOLVENT                DMSO
NS                       32
DS                        2
SWH             8012.820 Hz
FIDRES          0.122266 Hz
AQ             4.0894465 sec
RG                   129.43
DW                62.400 usec
DE                  6.50 usec
TE                   298.0 K
D1            1.00000000 sec
TD0                       1

======== CHANNEL f1 ========
SFO1         400.1924713 MHz
NUC1                     1H
P1                 15.00 usec
PLW1         10.39999962 W

F2 - Processing parameters
SI                    65536
SF           400.1900000 MHz
WDW                      EM
SSB       0
LB                  0.30 Hz
GB        0
PC                     1.00
```

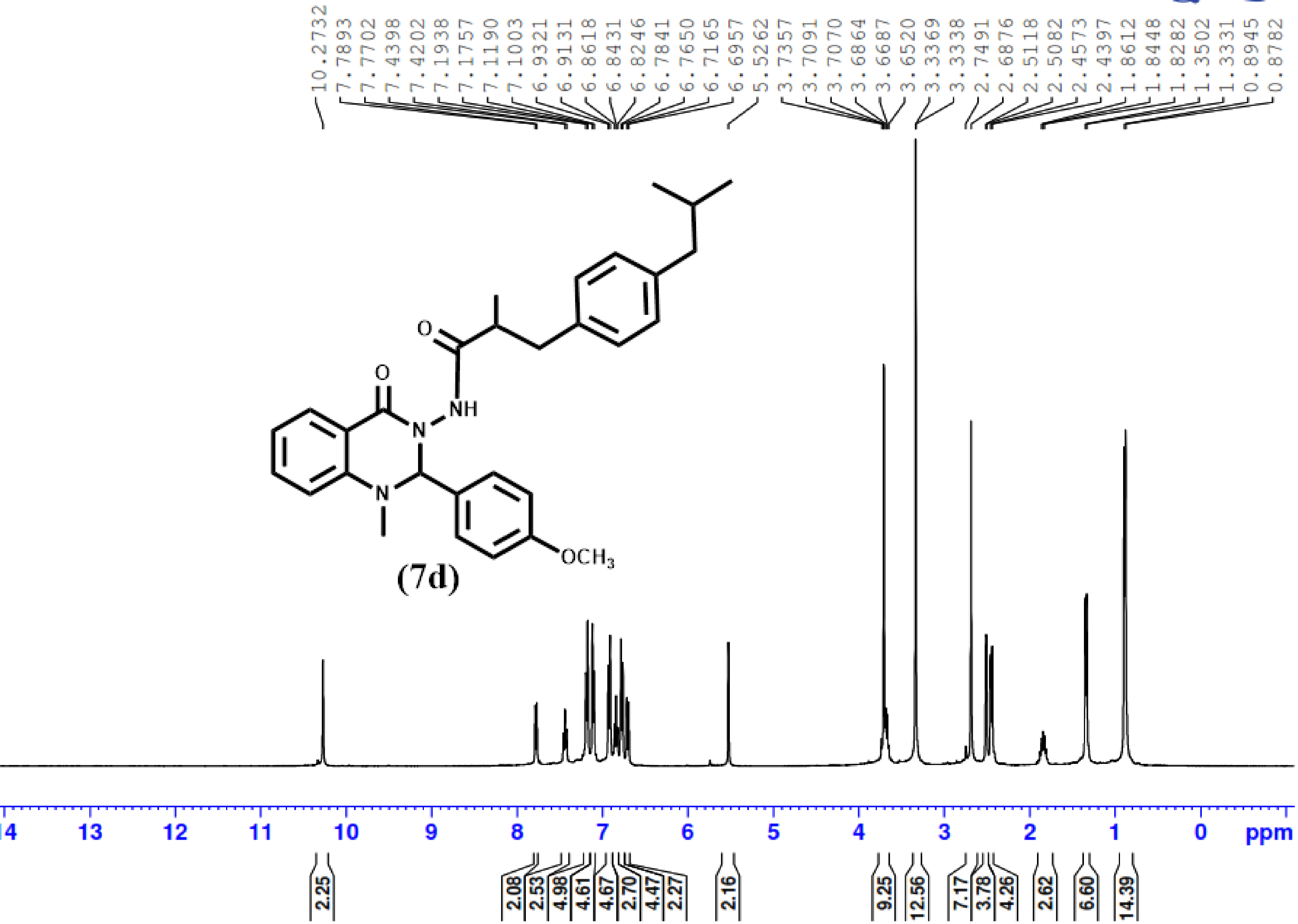

(7d)

Asmaa Alaa_H_BS-15_D2O

Microanalytical Unit - FOPCU - NMR laboratory
www.pharma.cu.edu.eg dir-mau.fopcu@pharma.cu.edu.eg

BRUKER

```
Current Data Parameters
NAME      Asmaa Alaa_H_BS-15_D2O
EXPNO                   10
PROCNO                   1

F2 - Acquisition Parameters
Date_             20180114
Time                 13.07
INSTRUM              spect
PROBHD    5 mm PABBO BB/
PULPROG              zg30
TD                   65536
SOLVENT               DMSO
NS                      32
DS                       2
SWH               8012.820 Hz
FIDRES            0.122266 Hz
AQ               4.0894465 sec
RG                  129.43
DW                  62.400 usec
DE                    6.50 usec
TE                   298.1 K
D1              1.00000000 sec
TD0                      1

======== CHANNEL f1 ========
SFO1           400.1924713 MHz
NUC1                    1H
P1                   15.00 usec
PLW1           10.39999962 W

F2 - Processing parameters
SI                   65536
SF             400.1900000 MHz
WDW                     EM
SSB       0
LB                    0.30 Hz
GB        0
PC                    1.00
```

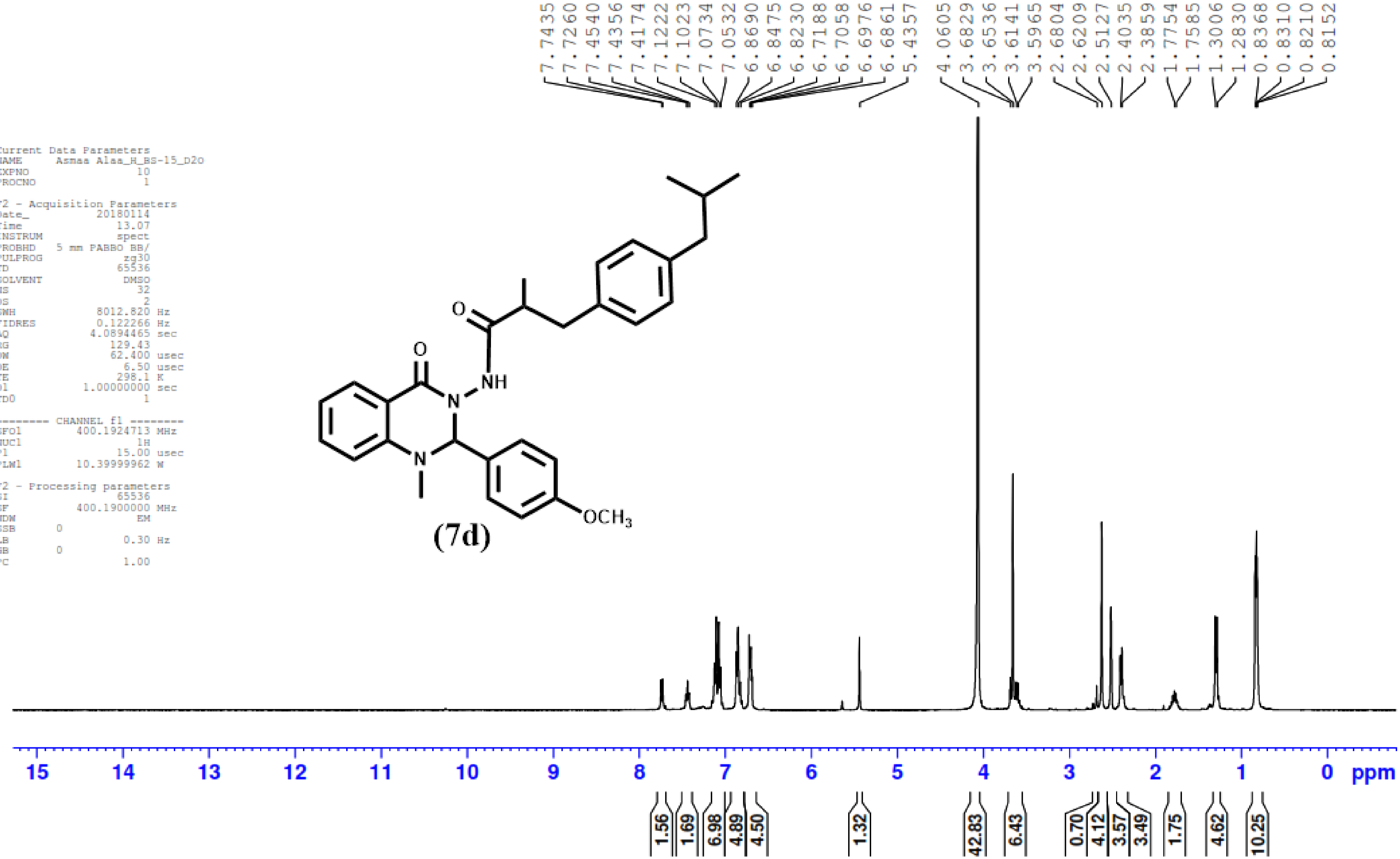

Asmaa Alaa_C_B-S15

Microanalytical Unit - FOPCU - NMR laboratory
www.pharma.cu.edu.eg dir-mau.fopcu@pharma.cu.edu.eg

BRUKER

```
Current Data Parameters
NAME      Asmaa Alaa_C_B-S15
EXPNO                   10
PROCNO                   1

F2 - Acquisition Parameters
Date_             20180111
Time                 21.54
INSTRUM              spect
PROBHD   5 mm PABBO BB/
PULPROG            zgpg30
TD                   65536
SOLVENT               DMSO
NS                    1200
DS                       4
SWH              24038.461 Hz
FIDRES            0.366798 Hz
AQ               1.3631488 sec
RG                  202.37
DW                  20.800 usec
DE                    6.50 usec
TE                   298.0 K
D1              2.00000000 sec
D11             0.03000000 sec
TD0                      1

======== CHANNEL f1 ========
SFO1         100.6379178 MHz
NUC1                   13C
P1                   10.00 usec
PLW1           45.00000000 W

======== CHANNEL f2 ========
SFO2         400.1916008 MHz
NUC2                    1H
CPDPRG[2           waltz16
```

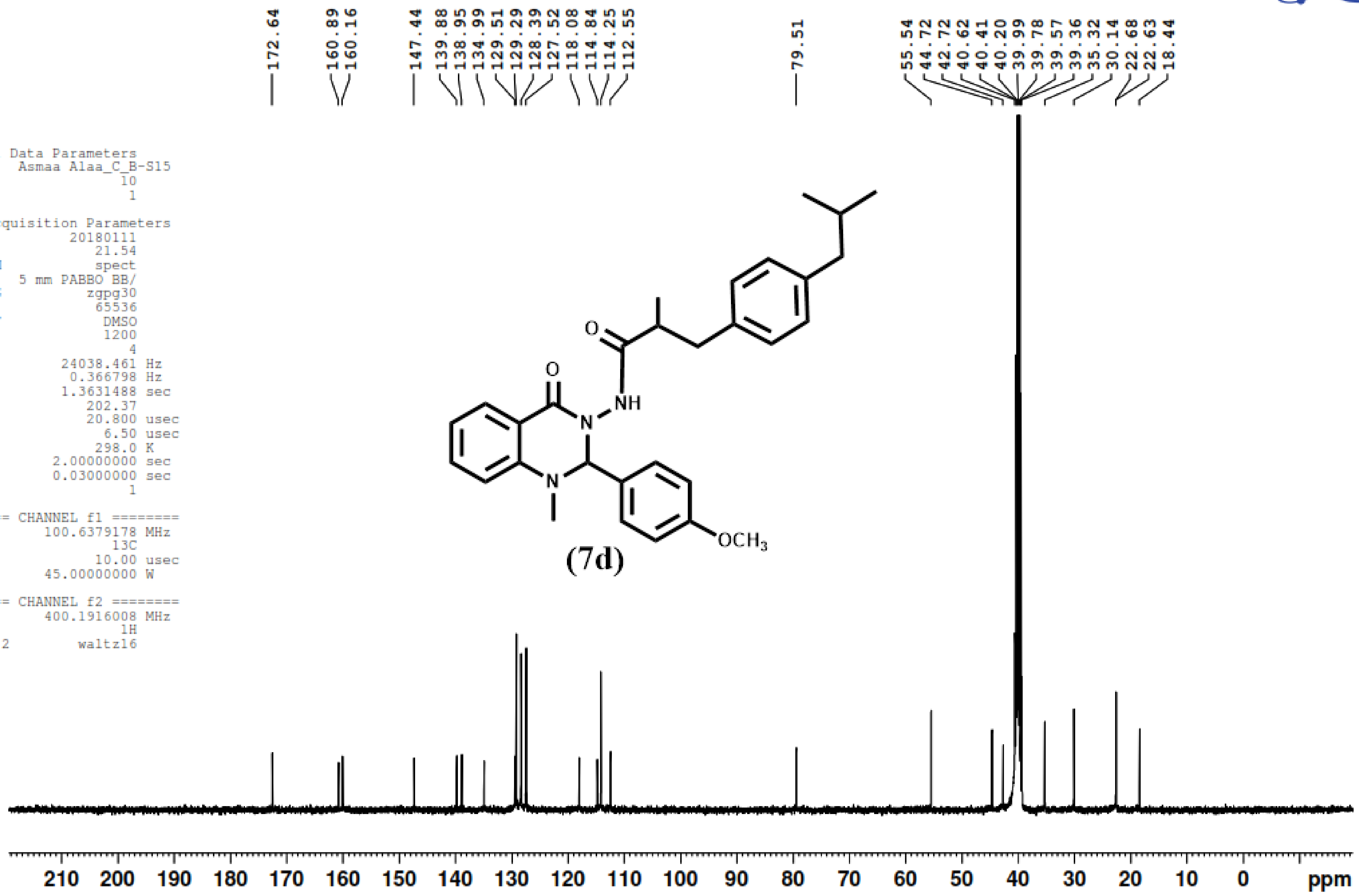

Asmaa Alaa_H_B-S16

Microanalytical Unit - FOPCU - NMR laboratory
www.pharma.cu.edu.eg dir-mau.fopcu@pharma.cu.edu.eg

BRUKER

```
Current Data Parameters
NAME     Asmaa Alaa_H_B-S16
EXPNO                  10
PROCNO                  1

F2 - Acquisition Parameters
Date_          20180423
Time               7.09
INSTRUM           spect
PROBHD   5 mm PABBO BB/
PULPROG            zg30
TD                65536
SOLVENT            DMSO
NS                   32
DS                    2
SWH            8012.820 Hz
FIDRES         0.122266 Hz
AQ            4.0894465 sec
RG               146.06
DW               62.400 usec
DE                 6.50 usec
TE                298.1 K
D1           1.00000000 sec
TD0                   1

======== CHANNEL f1 ========
SFO1        400.1924713 MHz
NUC1                 1H
P1                15.00 usec
PLW1        10.39999962 W

F2 - Processing parameters
SI                65536
SF          400.1900000 MHz
WDW                  EM
SSB      0
LB                 0.30 Hz
GB       0
PC                 1.00
```

(7e)

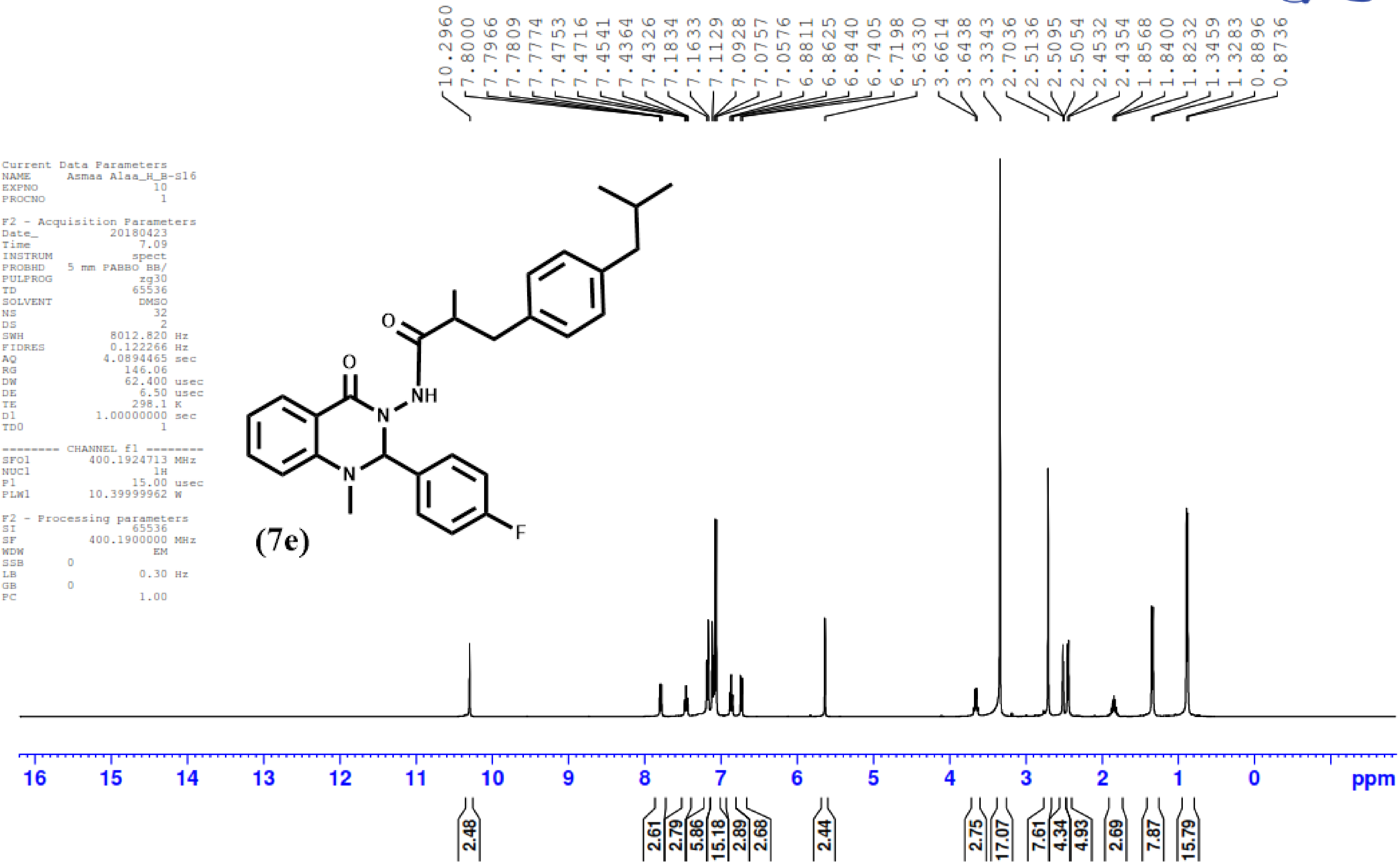

Asmaa Sakr_C_B-S16

Microanalytical Unit - FOPCU - NMR laboratory
www.pharma.cu.edu.eg dir-mau.fopcu@pharma.cu.edu.eg

BRUKER

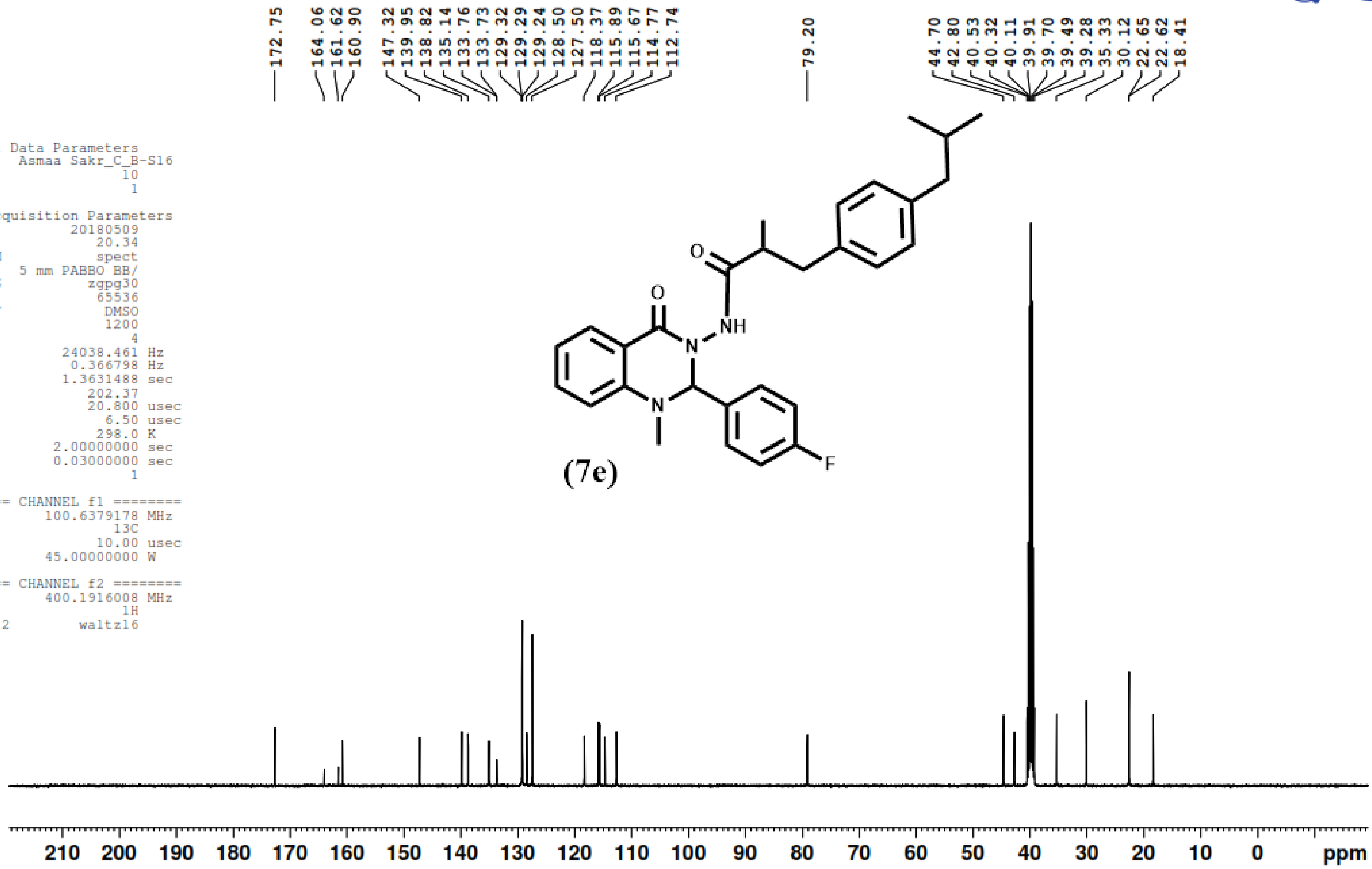

(7e)

```
Current Data Parameters
NAME      Asmaa Sakr_C_B-S16
EXPNO                     10
PROCNO                     1

F2 - Acquisition Parameters
Date_               20180509
Time                   20.34
INSTRUM                spect
PROBHD   5 mm PABBO BB/
PULPROG              zgpg30
TD                     65536
SOLVENT                 DMSO
NS                      1200
DS                         4
SWH              24038.461 Hz
FIDRES            0.366798 Hz
AQ               1.3631488 sec
RG                    202.37
DW                    20.800 usec
DE                      6.50 usec
TE                     298.0 K
D1               2.00000000 sec
D11              0.03000000 sec
TD0                        1

======== CHANNEL f1 ========
SFO1           100.6379178 MHz
NUC1                     13C
P1                     10.00 usec
PLW1           45.00000000 W

======== CHANNEL f2 ========
SFO2           400.1916008 MHz
NUC2                      1H
CPDPRG[2             waltz16
```

Asmaa Alaa_H_Sp2

Microanalytical Unit - FOPCU - NMR laboratory
www.pharma.cu.edu.eg dir-mau.fopcu@pharma.cu.edu.eg

BRUKER

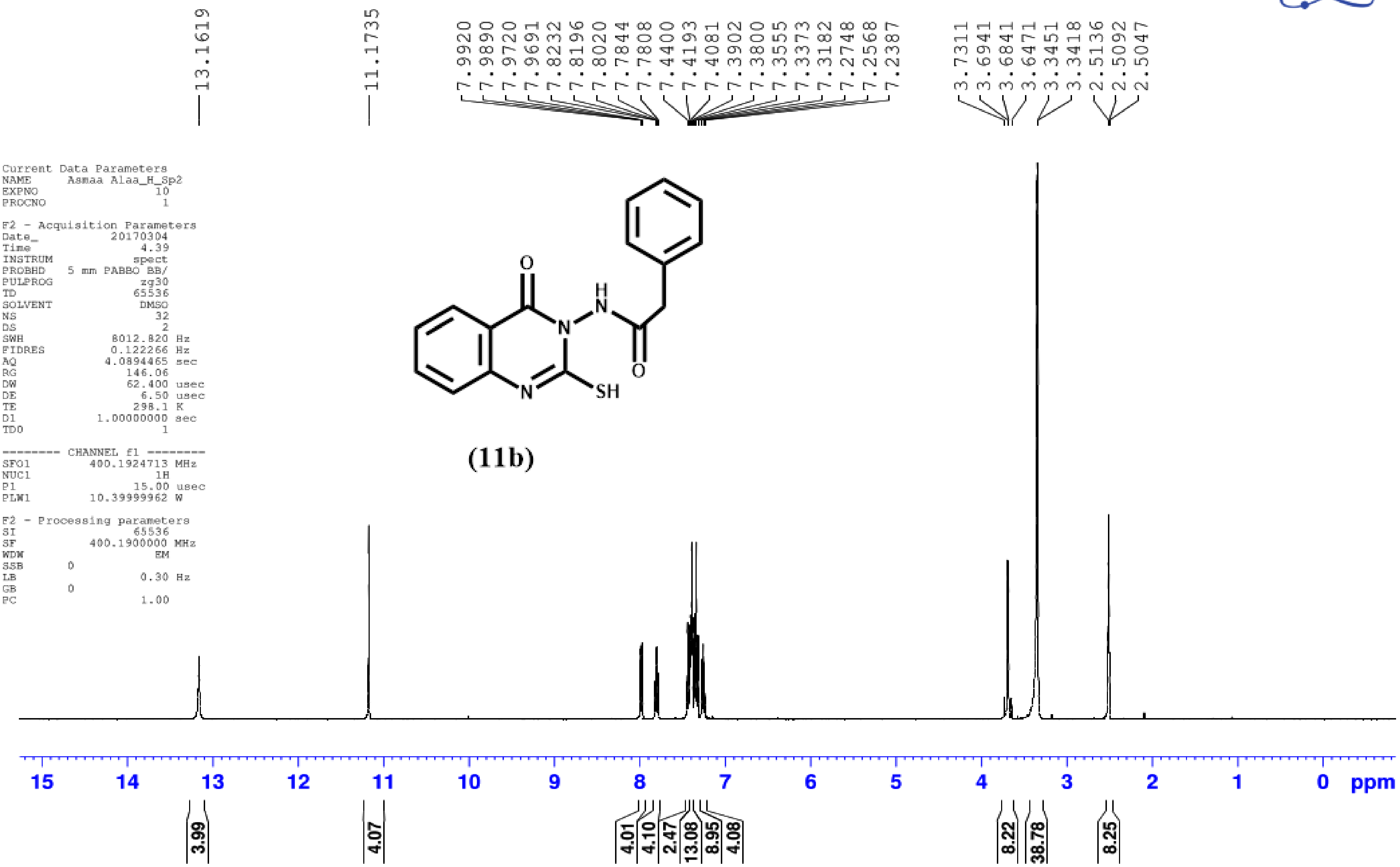


```
Current Data Parameters
NAME      Asmaa Alaa_H_Sp2
EXPNO                   10
PROCNO                   1

F2 - Acquisition Parameters
Date_             20170304
Time                  4.39
INSTRUM              spect
PROBHD   5 mm PABBO BB/
PULPROG               zg30
TD                   65536
SOLVENT               DMSO
NS                      32
DS                       2
SWH               8012.820 Hz
FIDRES            0.122266 Hz
AQ               4.0894465 sec
RG                  146.06
DW                  62.400 usec
DE                    6.50 usec
TE                   298.1 K
D1              1.00000000 sec
TD0                      1

======== CHANNEL f1 ========
SFO1           400.1924713 MHz
NUC1                    1H
P1                   15.00 usec
PLW1           10.39999962 W

F2 - Processing parameters
SI                   65536
SF             400.1900000 MHz
WDW                     EM
SSB      0
LB                    0.30 Hz
GB       0
PC                    1.00
```

(11b)

Asmaa Alaa_H_Sp2_D2O


```
Current Data Parameters
NAME       Asmaa Alaa_H_Sp2_D2O
EXPNO                     10
PROCNO                     1

F2 - Acquisition Parameters
Date_          20170305
Time              20.07
INSTRUM           spect
PROBHD   5 mm PABBO BB/
PULPROG            zg30
TD                65536
SOLVENT            DMSO
NS                   32
DS                    2
SWH            8012.820 Hz
FIDRES         0.122266 Hz
AQ            4.0894465 sec
RG               146.06
DW               62.400 usec
DE                 6.50 usec
TE                298.0 K
D1           1.00000000 sec
TD0                   1

======== CHANNEL f1 ========
SFO1        400.1924713 MHz
NUC1                 1H
P1                15.00 usec
PLW1        10.39999962 W

F2 - Processing parameters
SI                65536
SF          400.1900000 MHz
WDW                  EM
SSB      0
LB                 0.30 Hz
GB       0
PC                 1.00
```

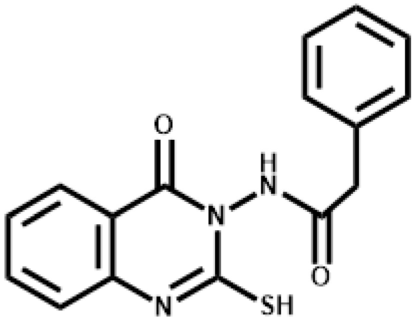

(11b)

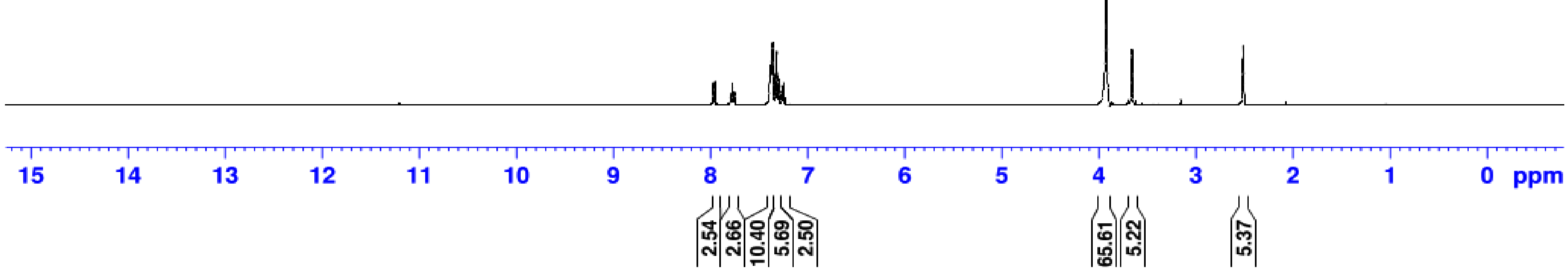

Asmaa Alaa_H_PH2

Microanalytical Unit - FOPCU - NMR laboratory
www.pharma.cu.edu.eg dir-mau.fopcu@pharma.cu.edu.eg

BRUKER

```
Current Data Parameters
NAME       Asmaa Alaa_H_PH2
EXPNO                    10
PROCNO                    1

F2 - Acquisition Parameters
Date_              20170605
Time                  12.40
INSTRUM               spect
PROBHD   5 mm PABBO BB/
PULPROG                zg30
TD                    65536
SOLVENT                DMSO
NS                       32
DS                        2
SWH             8012.820 Hz
FIDRES          0.122266 Hz
AQ             4.0894465 sec
RG                   114.95
DW               62.400 usec
DE                 6.50 usec
TE                298.0 K
D1           1.00000000 sec
TD0                       1

======== CHANNEL f1 ========
SFO1        400.1924713 MHz
NUC1                     1H
P1                15.00 usec
PLW1        10.39999962 W

F2 - Processing parameters
SI                    65536
SF          400.1900000 MHz
WDW                      EM
SSB       0
LB                  0.30 Hz
GB        0
PC                     1.00
```

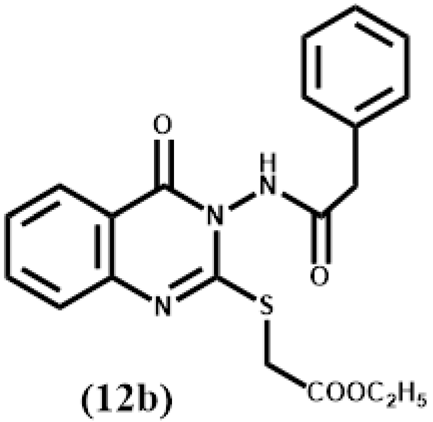

(12b)

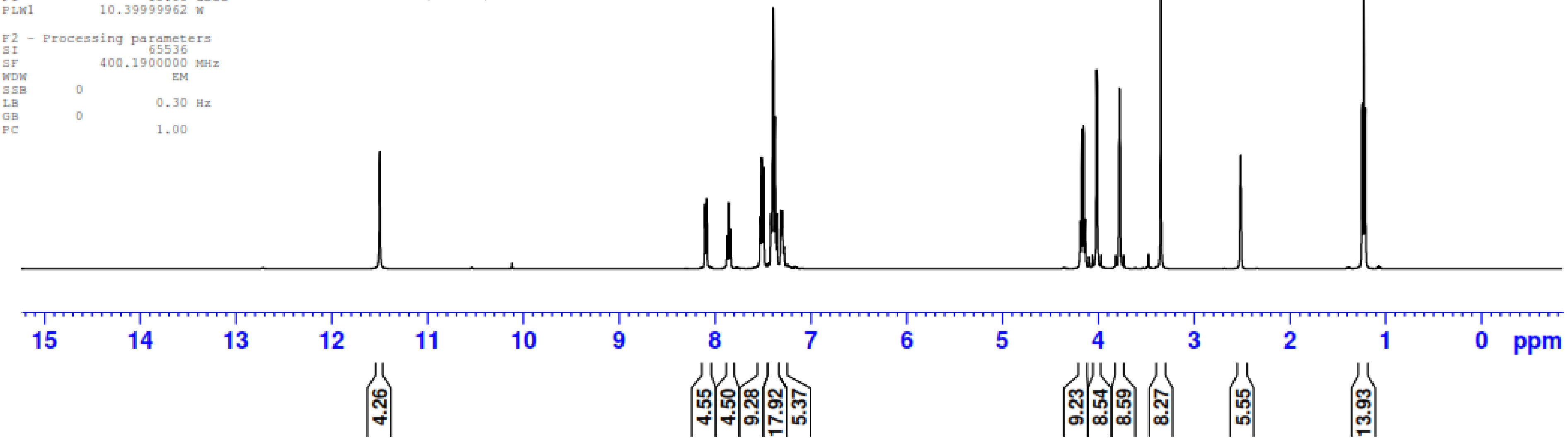

Asmaa Alaa_H_PH2_D2O

Microanalytical Unit - FOPCU - NMR laboratory
www.pharma.cu.edu.eg dir-mau.fopcu@pharma.cu.edu.eg

BRUKER

```
Current Data Parameters
NAME        Asmaa Alaa_H_PH2_D2O
EXPNO                     10
PROCNO                     1

F2 - Acquisition Parameters
Date_             20170605
Time                 14.25
INSTRUM              spect
PROBHD   5 mm PABBO BB/
PULPROG               zg30
TD                   65536
SOLVENT               DMSO
NS                      32
DS                       2
SWH               8012.820 Hz
FIDRES            0.122266 Hz
AQ               4.0894465 sec
RG                  114.95
DW                  62.400 usec
DE                    6.50 usec
TE                   298.0 K
D1             1.00000000 sec
TD0                      1

======== CHANNEL f1 ========
SFO1          400.1924713 MHz
NUC1                    1H
P1                   15.00 usec
PLW1          10.39999962 W

F2 - Processing parameters
SI                   65536
SF           400.1900000 MHz
WDW                     EM
SSB      0
LB                    0.30 Hz
GB       0
PC                    1.00
```

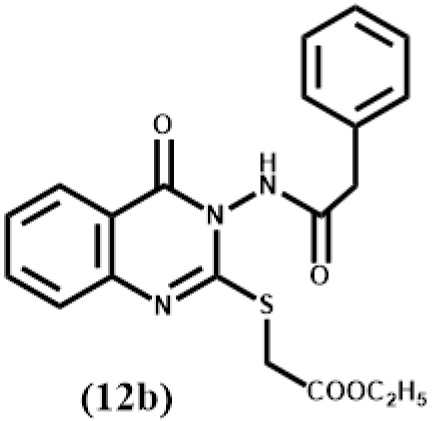

(12b)

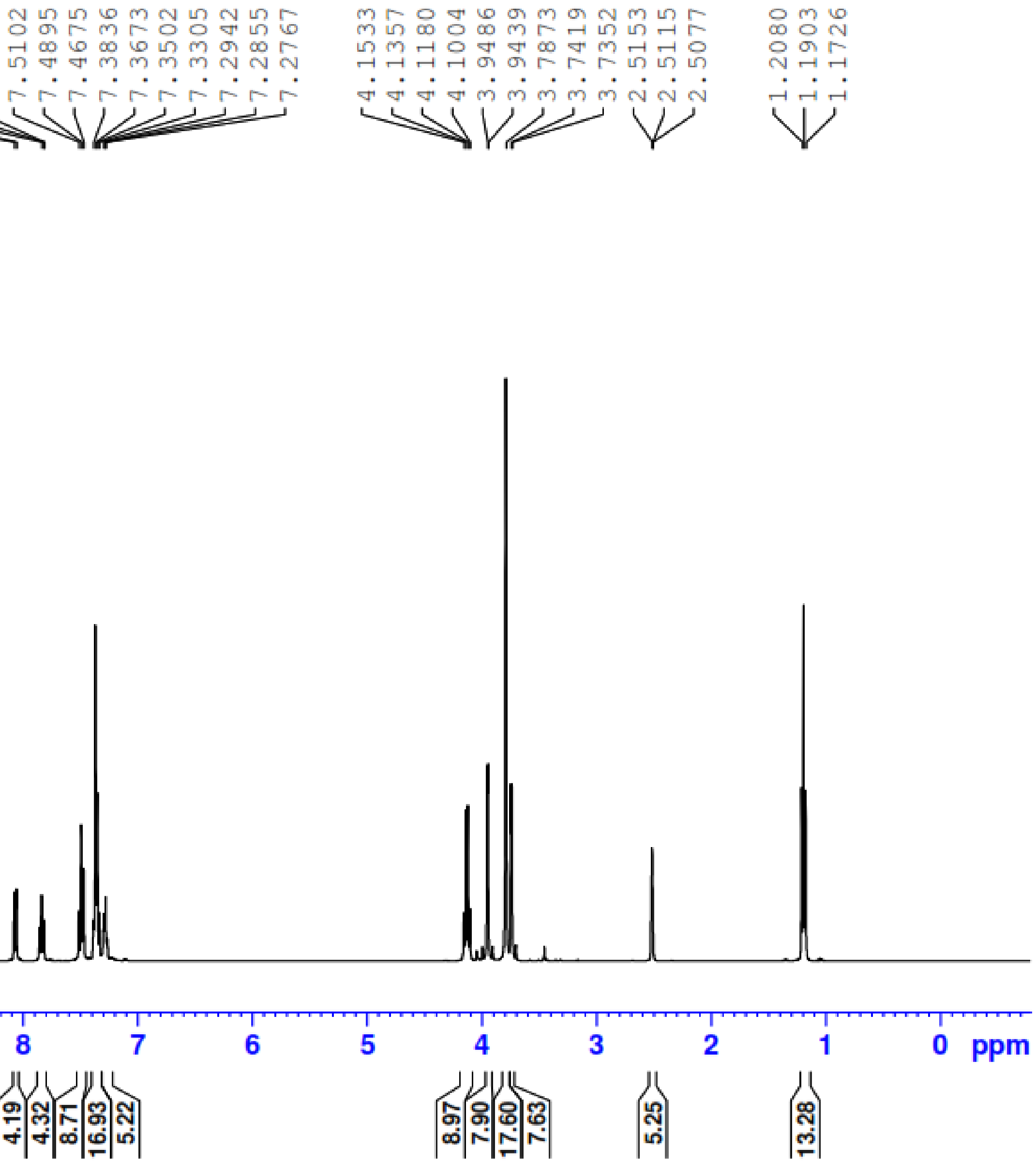

Asmaa Alaa_H_PO3#

Microanalytical Unit - FOPCU - NMR laboratory
www.pharma.cu.edu.eg dir-mau.fopcu@pharma.cu.edu.eg

BRUKER

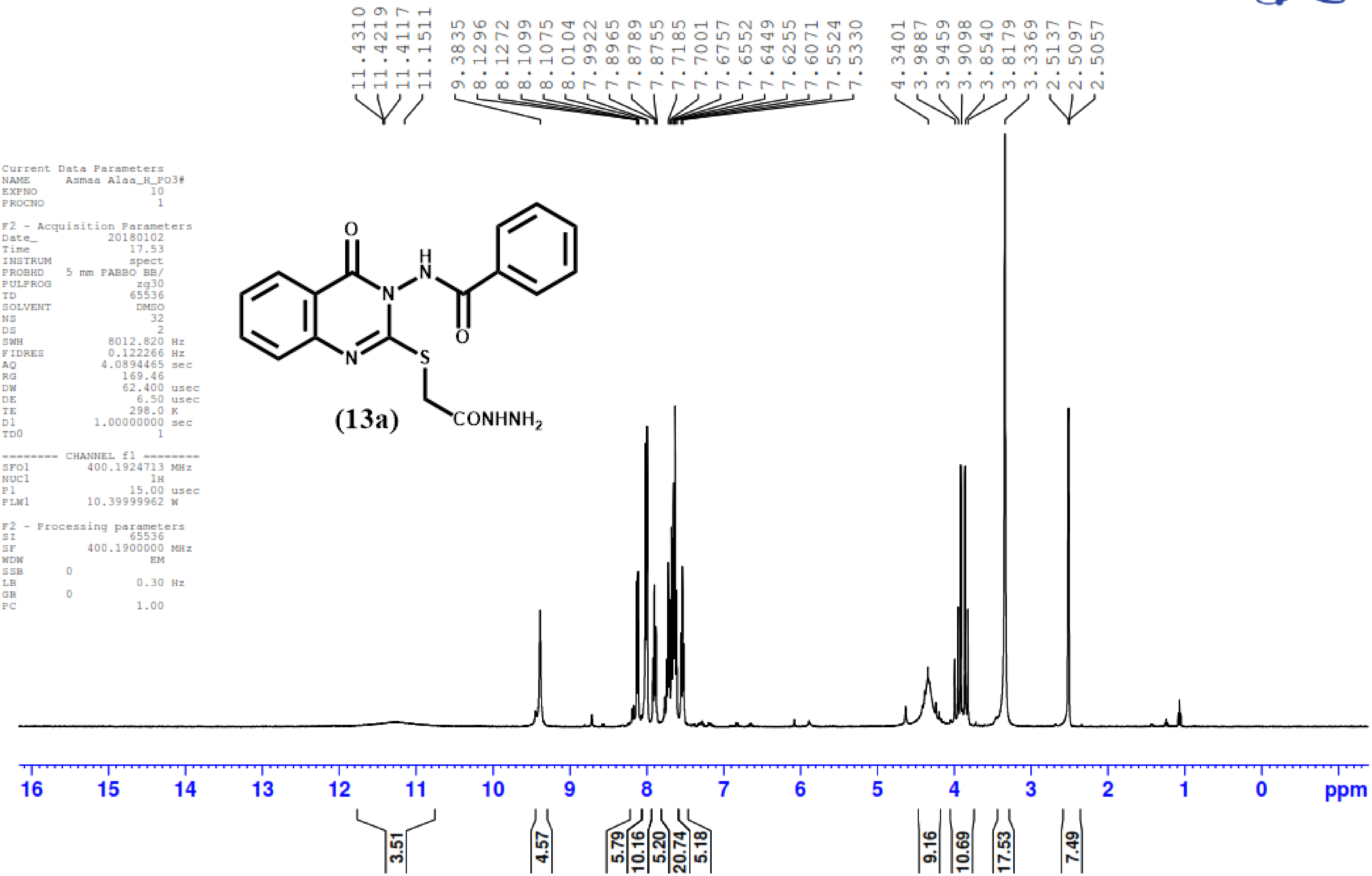

(13a)

Current Data Parameters
NAME Asmaa Alaa_H_PO3#
EXPNO 10
PROCNO 1

F2 - Acquisition Parameters
Date_ 20180102
Time 17.53
INSTRUM spect
PROBHD 5 mm PABBO BB/
PULPROG zg30
TD 65536
SOLVENT DMSO
NS 32
DS 2
SWH 8012.820 Hz
FIDRES 0.122266 Hz
AQ 4.0894465 sec
RG 169.46
DW 62.400 usec
DE 6.50 usec
TE 298.0 K
D1 1.00000000 sec
TD0 1

======== CHANNEL f1 ========
SFO1 400.1924713 MHz
NUC1 1H
P1 15.00 usec
PLW1 10.39999962 W

F2 - Processing parameters
SI 65536
SF 400.1900000 MHz
WDW EM
SSB 0
LB 0.30 Hz
GB 0
PC 1.00

Asmaa Alaa_H_PO3#_D2O

Microanalytical Unit - FOPCU - NMR laboratory
www.pharma.cu.edu.eg dir-mau.fopcu@pharma.cu.edu.eg

BRUKER

Current Data Parameters
NAME Asmaa Alaa_H_PO3#_D2O
EXPNO 10
PROCNO 1

F2 - Acquisition Parameters
Date_ 20180103
Time 13.55
INSTRUM spect
PROBHD 5 mm PABBO BB/
PULPROG zg30
TD 65536
SOLVENT DMSO
NS 32
DS 2
SWH 8012.820 Hz
FIDRES 0.122266 Hz
AQ 4.0894465 sec
RG 169.46
DW 62.400 usec
DE 6.50 usec
TE 298.1 K
D1 1.00000000 sec
TD0 1

======== CHANNEL f1 ========
SFO1 400.1924713 MHz
NUC1 1H
P1 15.00 usec
PLW1 10.39999962 W

F2 - Processing parameters
SI 65536
SF 400.1900000 MHz
WDW EM
SSB 0
LB 0.30 Hz
GB 0
PC 1.00

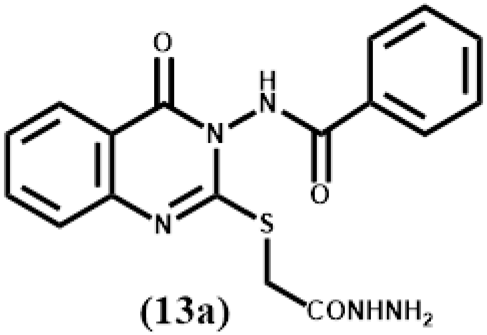

(13a)

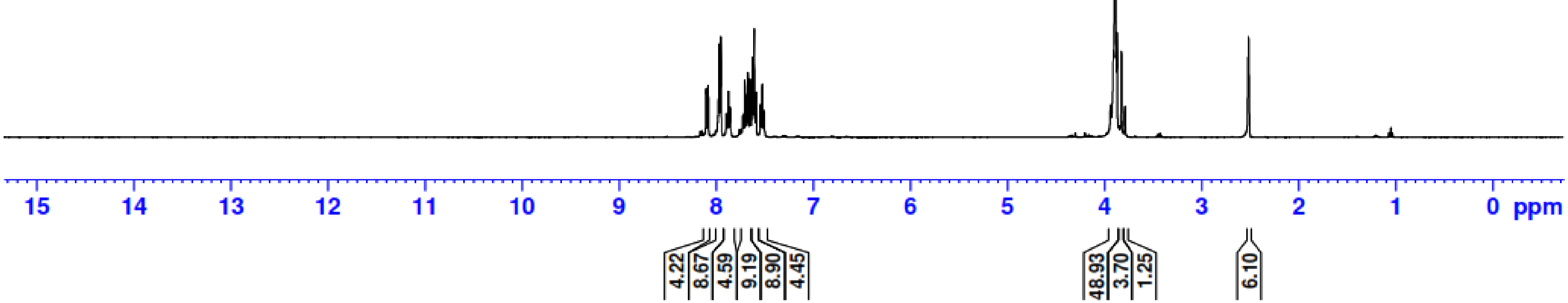

Asmaa Alaa_C_Po3

Microanalytical Unit - FOPCU - NMR laboratory
www.pharma.cu.edu.eg dir-mau.fopcu@pharma.cu.edu.eg

```
Current Data Parameters
NAME     Asmaa Alaa_C_Po3
EXPNO                  10
PROCNO                  1

F2 - Acquisition Parameters
Date_          20180111
Time              18.20
INSTRUM           spect
PROBHD   5 mm PABBO BB/
PULPROG         zgpg30
TD               65536
SOLVENT           DMSO
NS                1200
DS                   4
SWH          24038.461 Hz
FIDRES        0.366798 Hz
AQ           1.3631488 sec
RG              202.37
DW              20.800 usec
DE                6.50 usec
TE               298.0 K
D1          2.00000000 sec
D11         0.03000000 sec
TD0                  1

======== CHANNEL f1 ========
SFO1       100.6379178 MHz
NUC1               13C
P1               10.00 usec
PLW1       45.00000000 W

======== CHANNEL f2 ========
SFO2       400.1916008 MHz
NUC2                1H
CPDPRG[2        waltz16
```

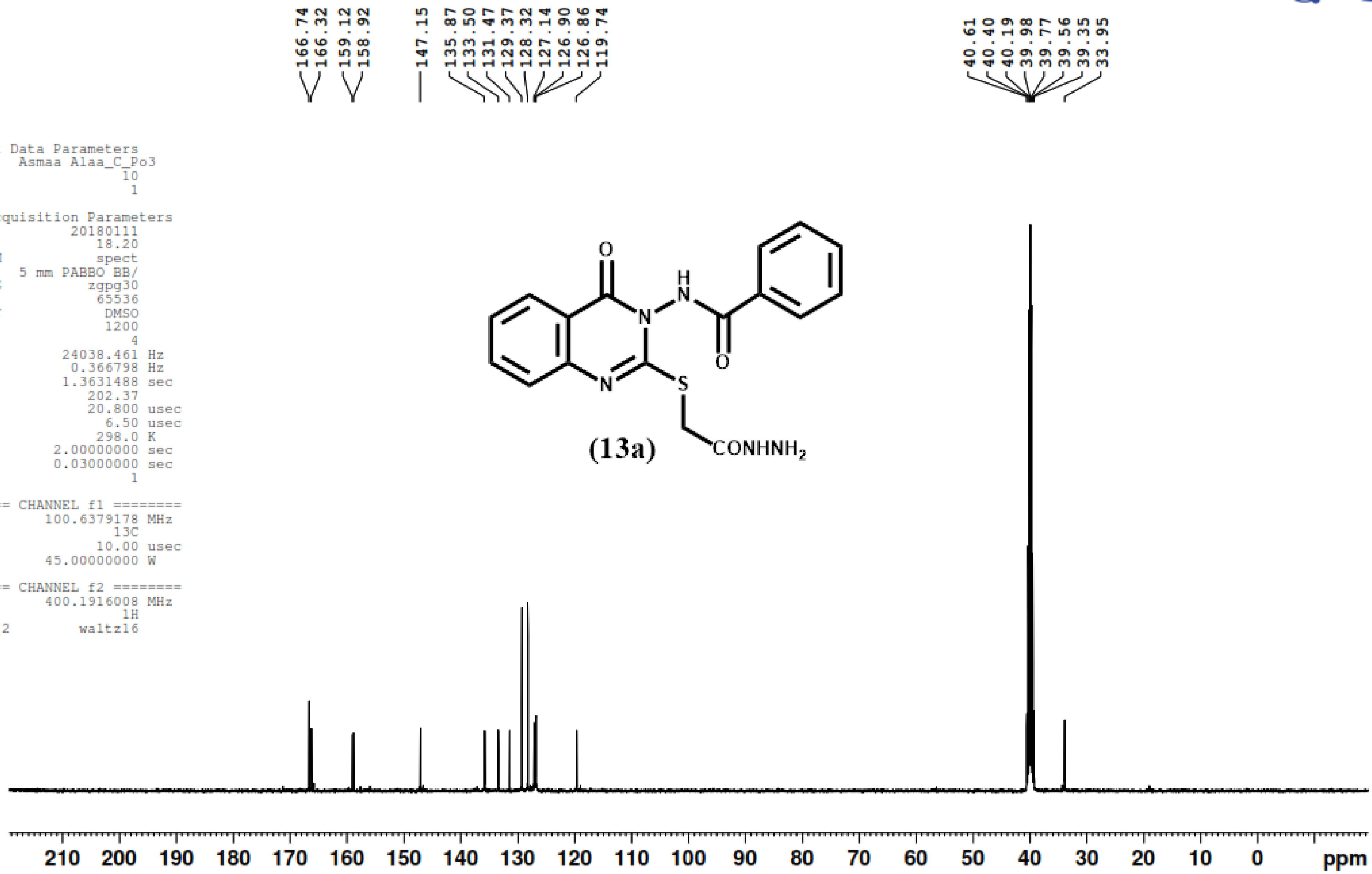

Asmaa Alaa_H_PH1

Microanalytical Unit - FOPCU - NMR laboratory
www.pharma.cu.edu.eg dir-mau.fopcu@pharma.cu.edu.eg

Current Data Parameters
NAME Asmaa Alaa_H_PH1
EXPNO 21
PROCNO 1

F2 - Acquisition Parameters
Date_ 20170605
Time 12.30
INSTRUM spect
PROBHD 5 mm PABBO BB/
PULPROG zg30
TD 65536
SOLVENT DMSO
NS 32
DS 2
SWH 8012.820 Hz
FIDRES 0.122266 Hz
AQ 4.0894465 sec
RG 129.43
DW 62.400 usec
DE 6.50 usec
TE 298.0 K
D1 1.00000000 sec
TD0 1

======== CHANNEL f1 ========
SFO1 400.1924713 MHz
NUC1 1H
P1 15.00 usec
PLW1 10.39999962 W

F2 - Processing parameters
SI 65536
SF 400.1900000 MHz
WDW EM
SSB 0
LB 0.30 Hz
GB 0
PC 1.00

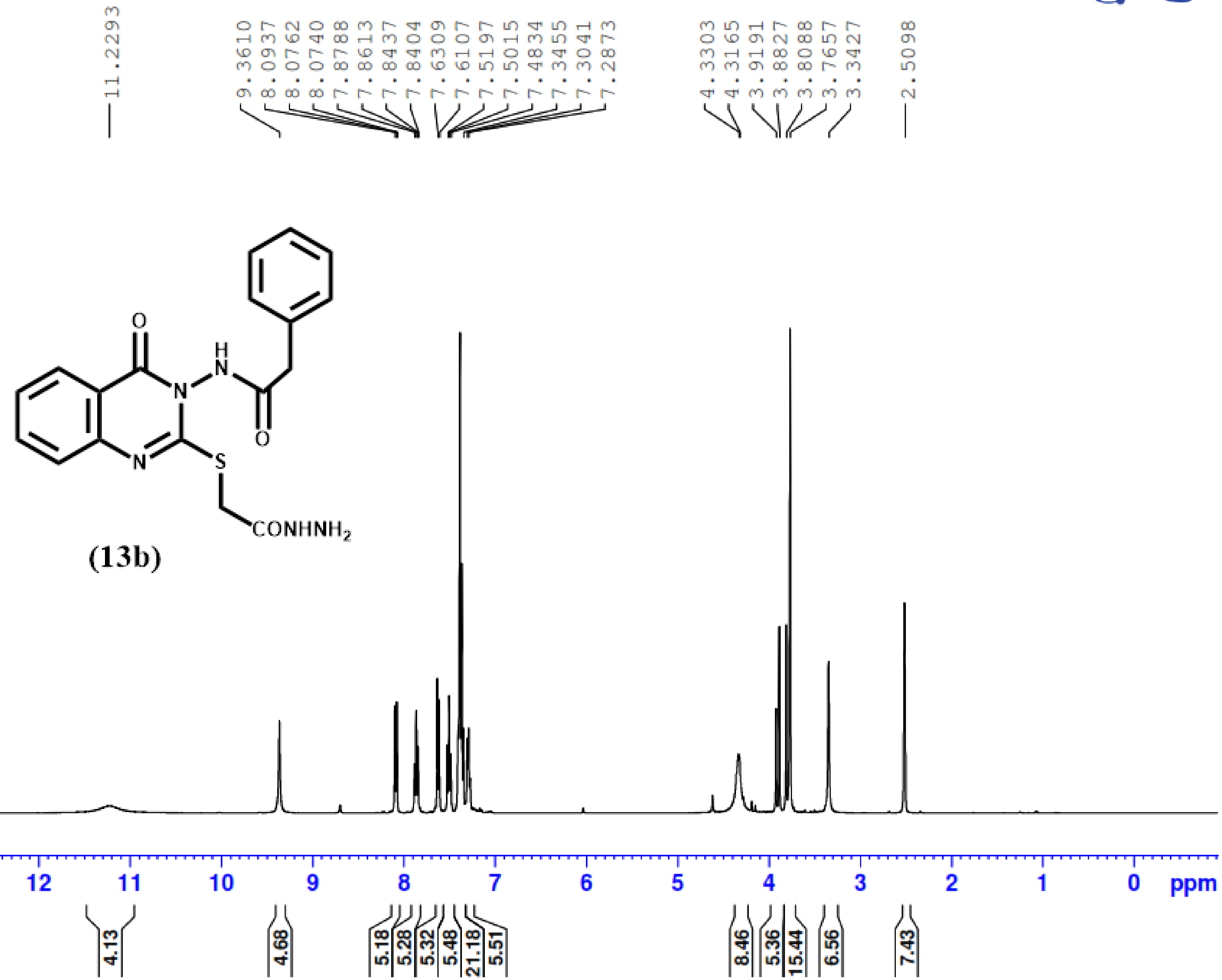

Asmaa Alaa_H_PH1_D2O

Microanalytical Unit - FOPCU - NMR laboratory
www.pharma.cu.edu.eg dir-mau.fopcu@pharma.cu.edu.eg

```
Current Data Parameters
NAME     Asmaa Alaa_H_PH1_D2O
EXPNO                  21
PROCNO                  1

F2 - Acquisition Parameters
Date_            20170618
Time                15.53
INSTRUM             spect
PROBHD   5 mm PABBO BB/
PULPROG              zg30
TD                  65536
SOLVENT              DMSO
NS                     32
DS                      2
SWH            8012.820 Hz
FIDRES         0.122266 Hz
AQ            4.0894465 sec
RG                  64.21
DW               62.400 usec
DE                 6.50 usec
TE                298.1 K
D1           1.00000000 sec
TD0                     1

======== CHANNEL f1 ========
SFO1        400.1924713 MHz
NUC1                   1H
P1                15.00 usec
PLW1        10.39999962 W

F2 - Processing parameters
SI                  65536
SF          400.1900000 MHz
WDW                    EM
SSB       0
LB                0.30 Hz
GB        0
PC                   1.00
```

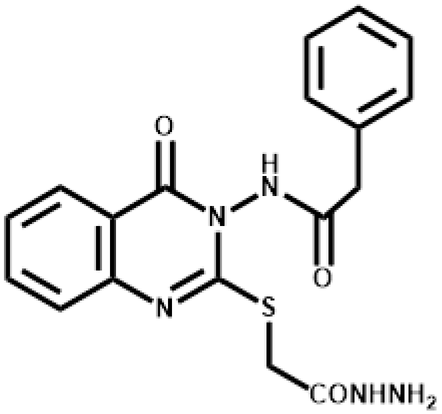

(13b)

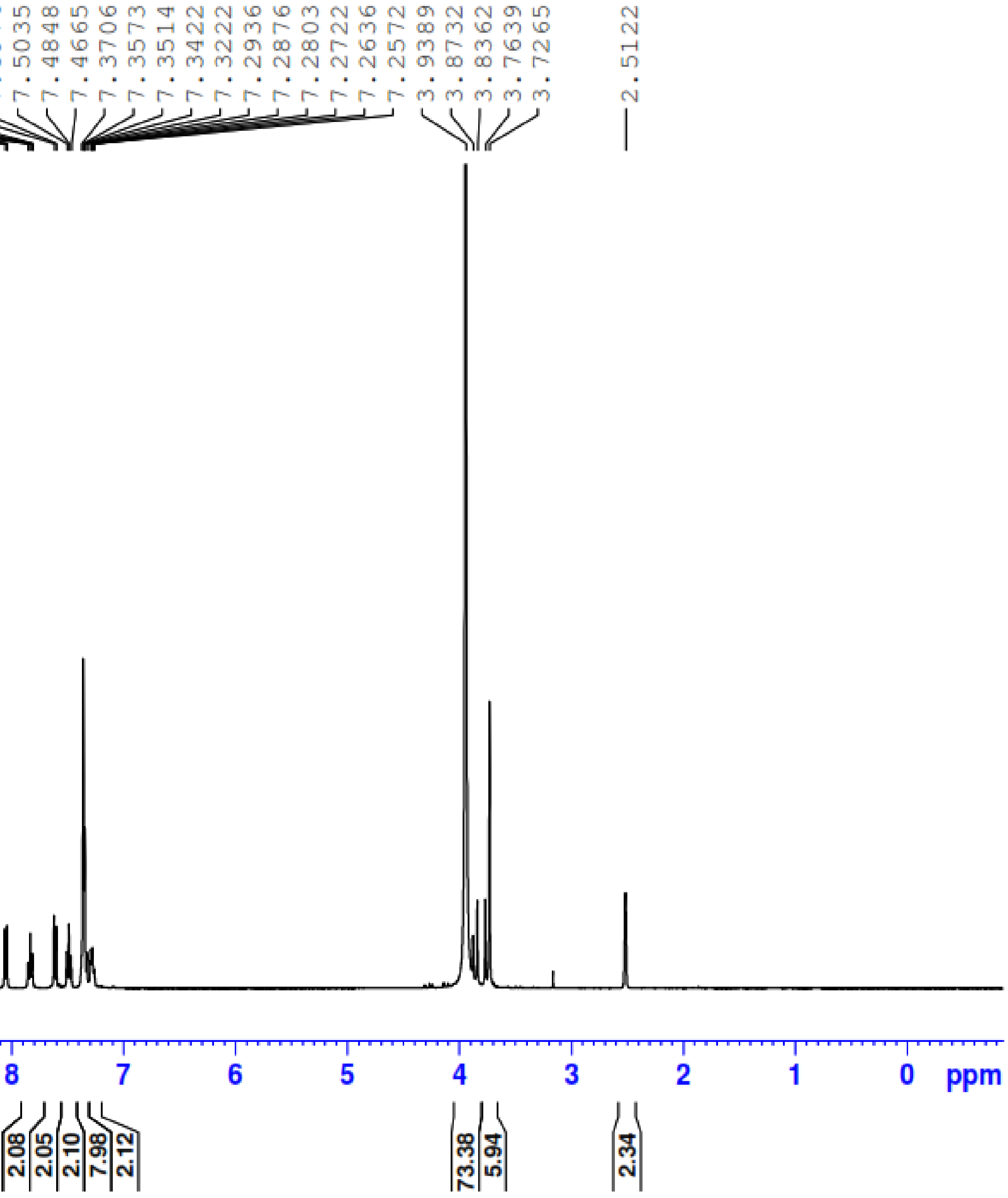

Asmaa Alaa_C_PH1

Microanalytical Unit - FOPCU - NMR laboratory
www.pharma.cu.edu.eg dir-mau.fopcu@pharma.cu.edu.eg

Current Data Parameters
NAME Asmaa Alaa_C_PH1
EXPNO 21
PROCNO 1

F2 - Acquisition Parameters
Date_ 20170616
Time 8.01
INSTRUM spect
PROBHD 5 mm PABBO BB/
PULPROG zgpg30
TD 65536
SOLVENT DMSO
NS 1200
DS 4
SWH 24038.461 Hz
FIDRES 0.366798 Hz
AQ 1.3631488 sec
RG 202.37
DW 20.800 usec
DE 6.50 usec
TE 298.1 K
D1 2.00000000 sec
D11 0.03000000 sec
TD0 1

======== CHANNEL f1 ========
SFO1 100.6379178 MHz
NUC1 13C
P1 10.00 usec
PLW1 45.00000000 W

======== CHANNEL f2 ========
SFO2 400.1916008 MHz
NUC2 1H
CPDPRG[2 waltz16

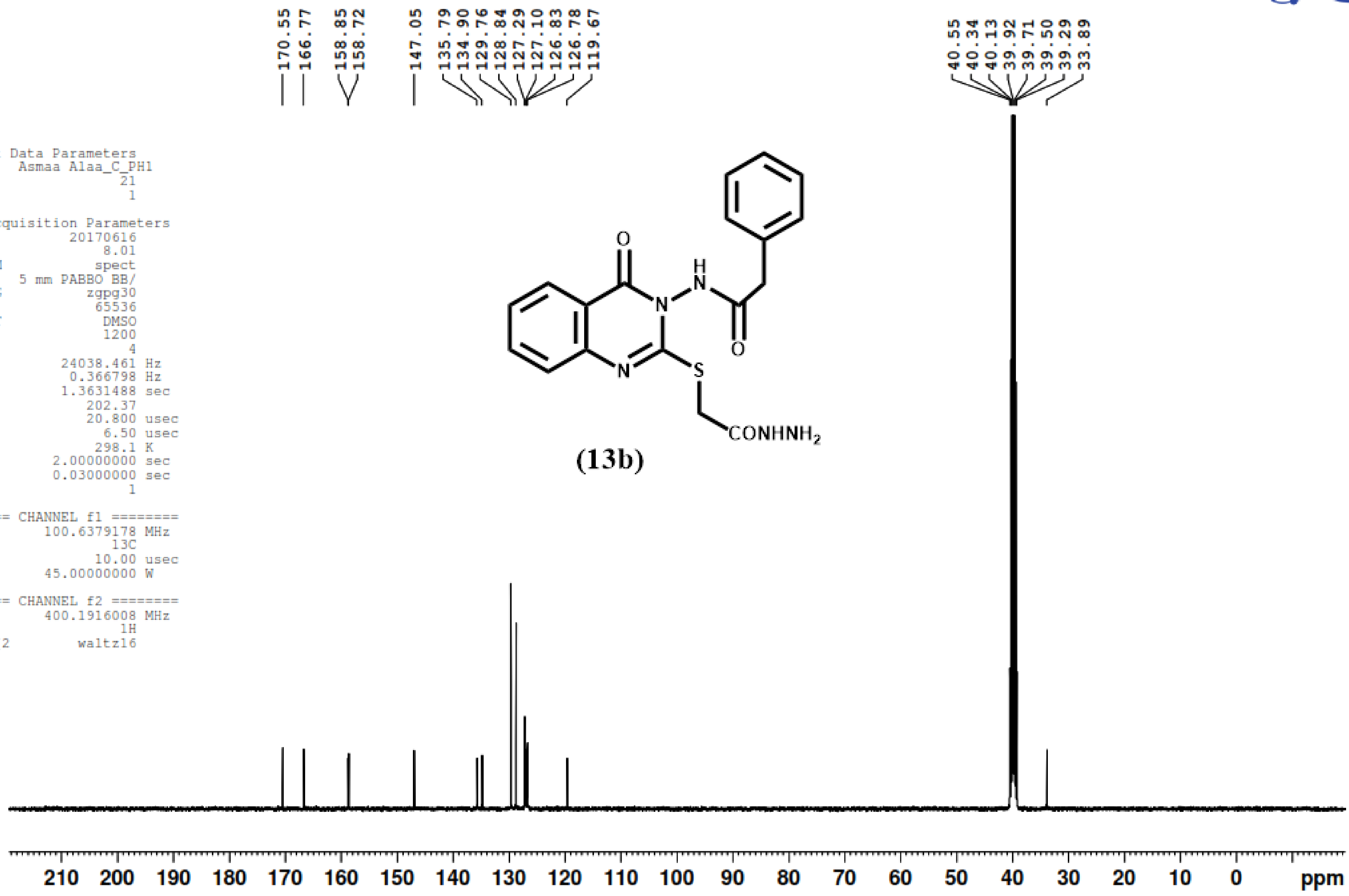

(13b)

asmaa alaa Ph1-M-carbon

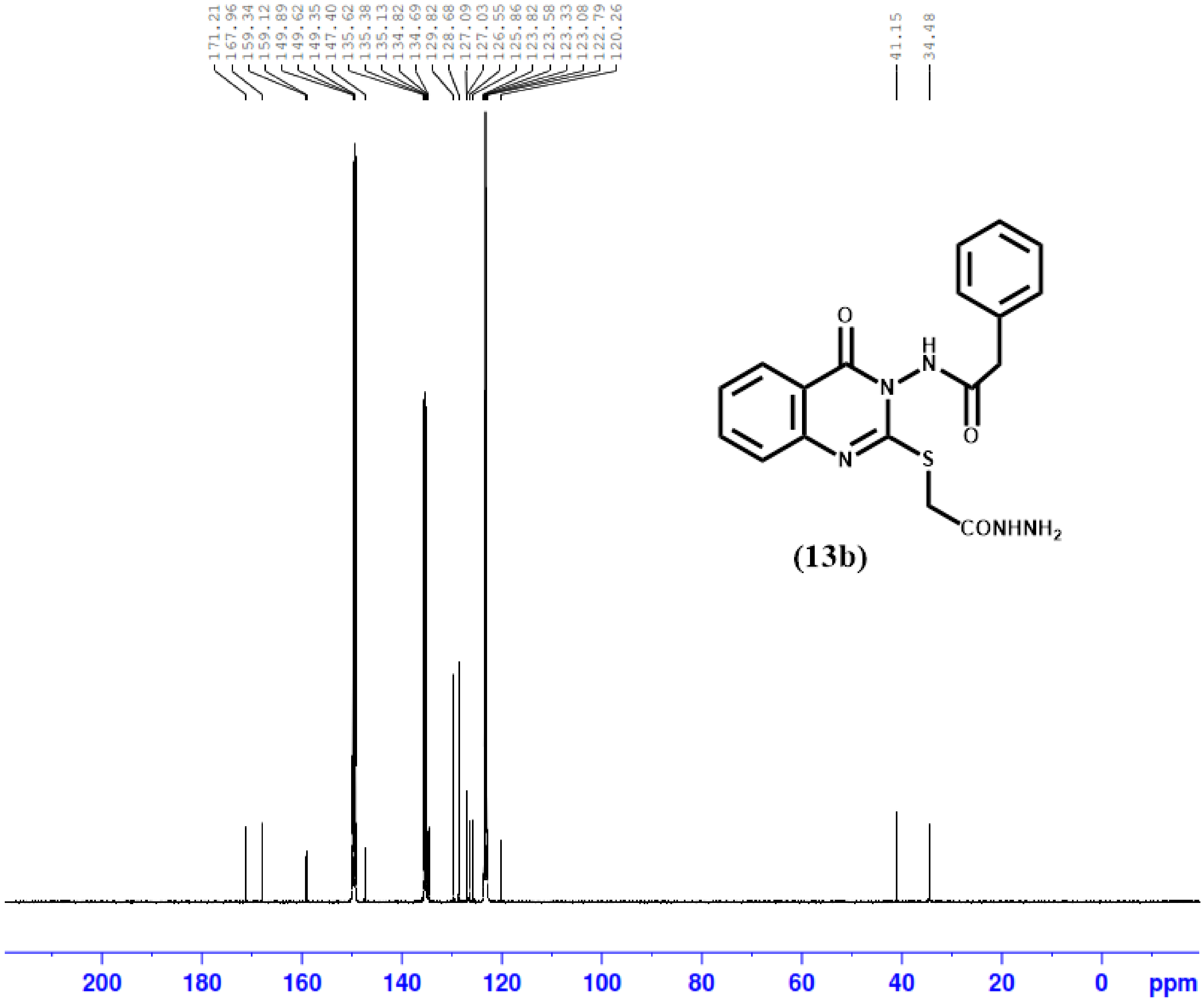

BRUKER

Faculty of Pharmacy

Current Data Parameters
NAME asmaa alaa Ph1-M
EXPNO 10
PROCNO 1

F2 - Acquisition Parameters
Date_ 20180507
Time 21.32 h
INSTRUM spect
PROBHD Z108618_0945 (
PULPROG zgpg30
TD 65536
SOLVENT Pyr
NS 2100
DS 4
SWH 24038.461 Hz
FIDRES 0.733596 Hz
AQ 1.3631488 sec
RG 197.77
DW 20.800 usec
DE 6.50 usec
TE 293.9 K
D1 2.00000000 sec
D11 0.03000000 sec
TD0 1
SFO1 100.6404331 MHz
NUC1 13C
P1 10.00 usec
PLW1 47.00000000 W
SFO2 400.2016008 MHz
NUC2 1H
CPDPRG[2 waltz16
PCPD2 90.00 usec
PLW2 13.00000000 W
PLW12 0.29249999 W
PLW13 0.14713000 W

F2 - Processing parameters
SI 32768
SF 100.6303700 MHz
WDW EM
SSB 0
LB 1.00 Hz
GB 0
PC 1.40

Asmaa Alaa_H_PO4

Microanalytical Unit - FOPCU - NMR laboratory
www.pharma.cu.edu.eg dir-mau.fopcu@pharma.cu.edu.eg

BRUKER

```
Current Data Parameters
NAME     Asmaa Alaa_H_PO4
EXPNO                   10
PROCNO                   1

F2 - Acquisition Parameters
Date_           20180102
Time               17.58
INSTRUM            spect
PROBHD   5 mm PABBO BB/
PULPROG             zg30
TD                 65536
SOLVENT             DMSO
NS                    32
DS                     2
SWH            8012.820 Hz
FIDRES         0.122266 Hz
AQ            4.0894465 sec
RG                169.46
DW               62.400 usec
DE                 6.50 usec
TE                298.0 K
D1           1.00000000 sec
TD0                    1

======== CHANNEL f1 ========
SFO1        400.1924713 MHz
NUC1                 1H
P1                15.00 usec
PLW1        10.39999962 W

F2 - Processing parameters
SI                 65536
SF          400.1900000 MHz
WDW                   EM
SSB       0
LB                 0.30 Hz
GB        0
PC                 1.00
```

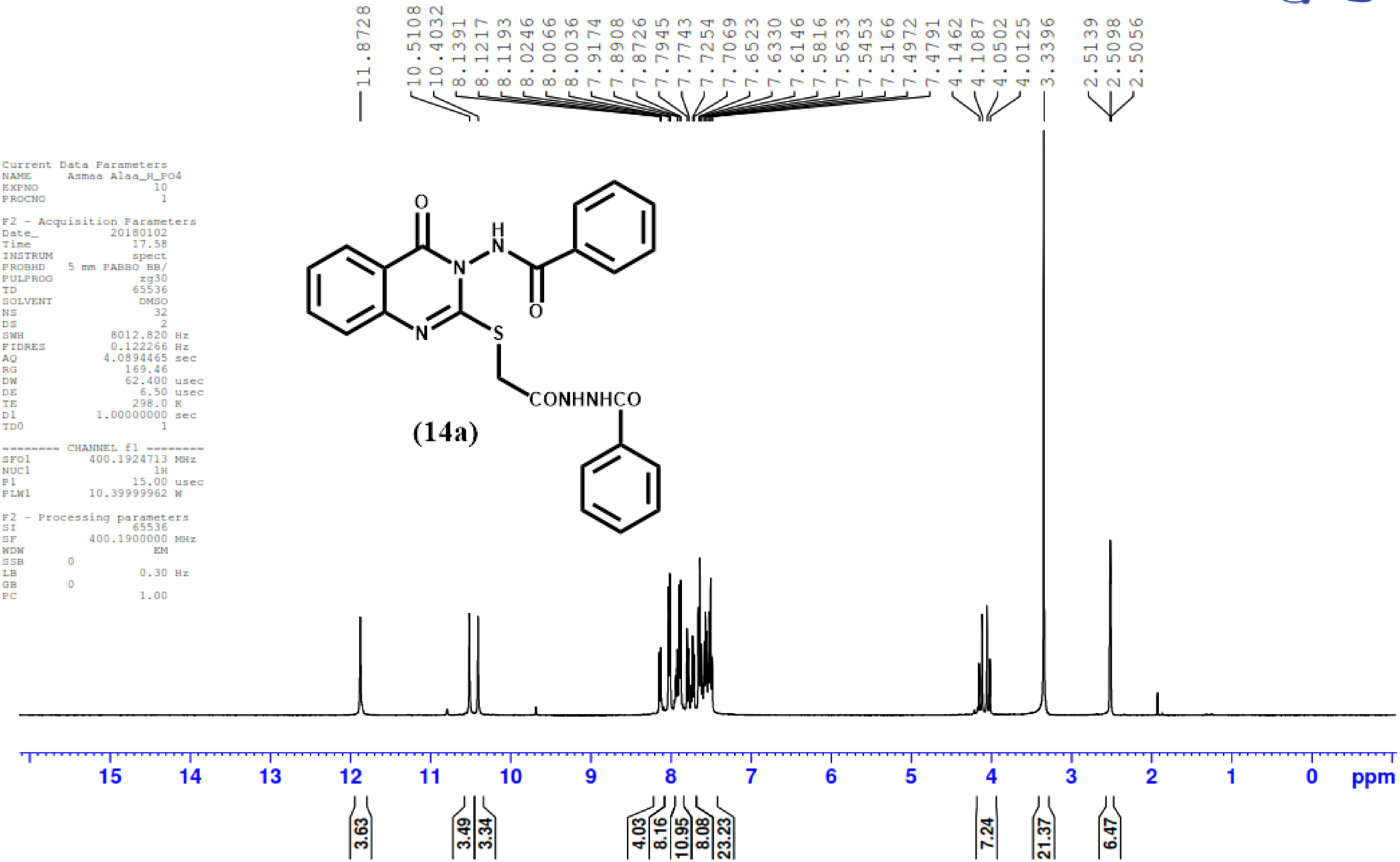

(14a)

Asmaa Alaa_H_PO4_D2O

Microanalytical Unit - FOPCU - NMR laboratory
www.pharma.cu.edu.eg dir-mau.fopcu@pharma.cu.edu.eg

```
Current Data Parameters
NAME      Asmaa Alaa_H_PO4_D2O
EXPNO                   10
PROCNO                   1

F2 - Acquisition Parameters
Date_            20180114
Time                13.12
INSTRUM             spect
PROBHD   5 mm PABBO BB/
PULPROG              zg30
TD                  65536
SOLVENT              DMSO
NS                     32
DS                      2
SWH              8012.820 Hz
FIDRES           0.122266 Hz
AQ              4.0894465 sec
RG                 114.95
DW                 62.400 usec
DE                   6.50 usec
TE                  298.0 K
D1             1.00000000 sec
TD0                     1

======== CHANNEL f1 ========
SFO1         400.1924713 MHz
NUC1                  1H
P1                 15.00 usec
PLW1         10.39999962 W

F2 - Processing parameters
SI                  65536
SF           400.1900000 MHz
WDW                    EM
SSB       0
LB                   0.30 Hz
GB        0
PC                   1.00
```

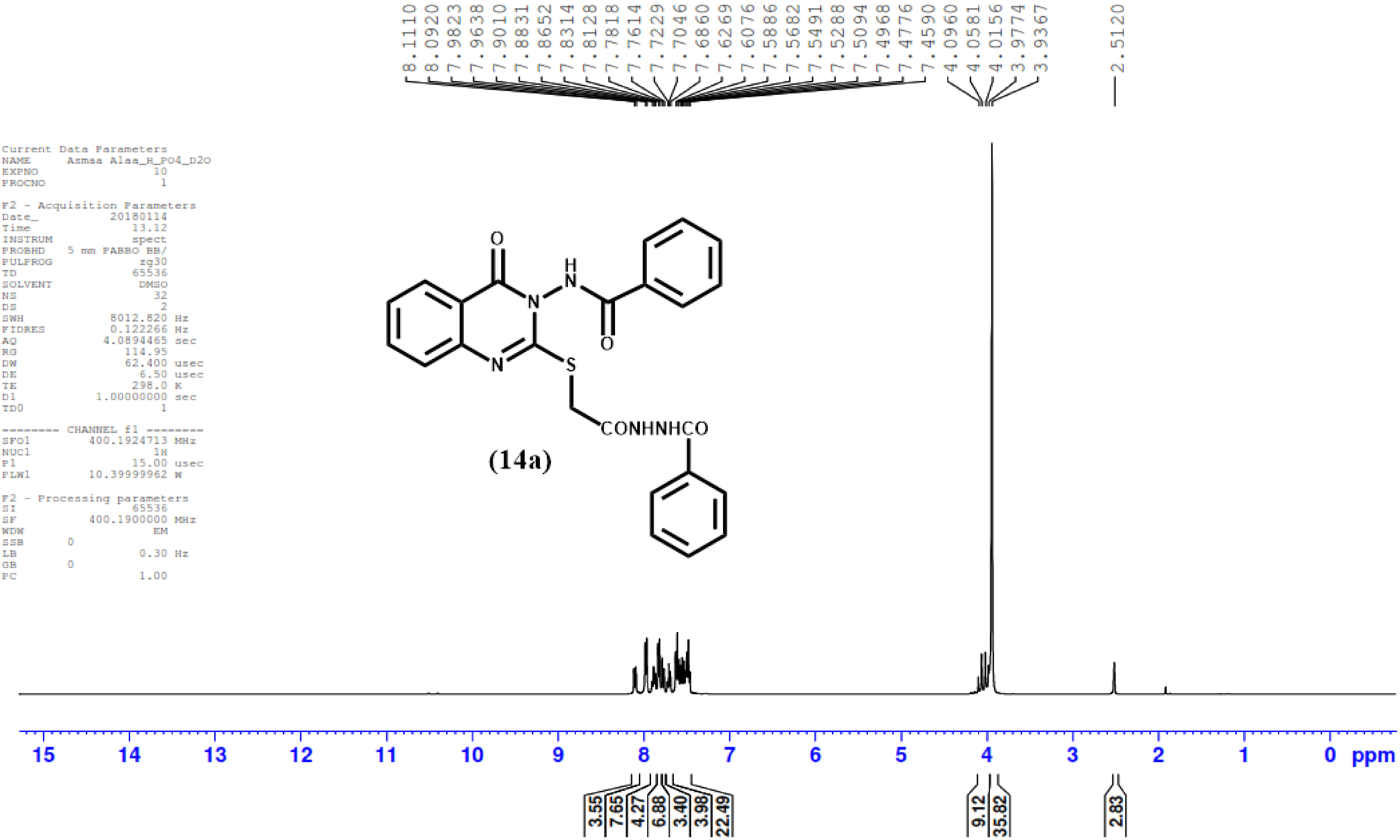

(14a)

Asmaa Alaa_C_Po4

Microanalytical Unit - FOPCU - NMR laboratory
www.pharma.cu.edu.eg dir-mau.fopcu@pharma.cu.edu.eg

BRUKER

```
Current Data Parameters
NAME     Asmaa Alaa_C_Po4
EXPNO                  10
PROCNO                  1

F2 - Acquisition Parameters
Date_            20180111
Time                20.42
INSTRUM             spect
PROBHD    5 mm PABBO BB/
PULPROG           zgpg30
TD                 65536
SOLVENT             DMSO
NS                  1200
DS                     4
SWH            24038.461 Hz
FIDRES          0.366798 Hz
AQ             1.3631488 sec
RG                202.37
DW                20.800 usec
DE                  6.50 usec
TE                 298.0 K
D1            2.00000000 sec
D11           0.03000000 sec
TD0                    1

======== CHANNEL f1 ========
SFO1         100.6379178 MHz
NUC1                 13C
P1                 10.00 usec
PLW1         45.00000000 W

======== CHANNEL f2 ========
SFO2         400.1916008 MHz
NUC2                  1H
CPDPRG[2          waltz16
```

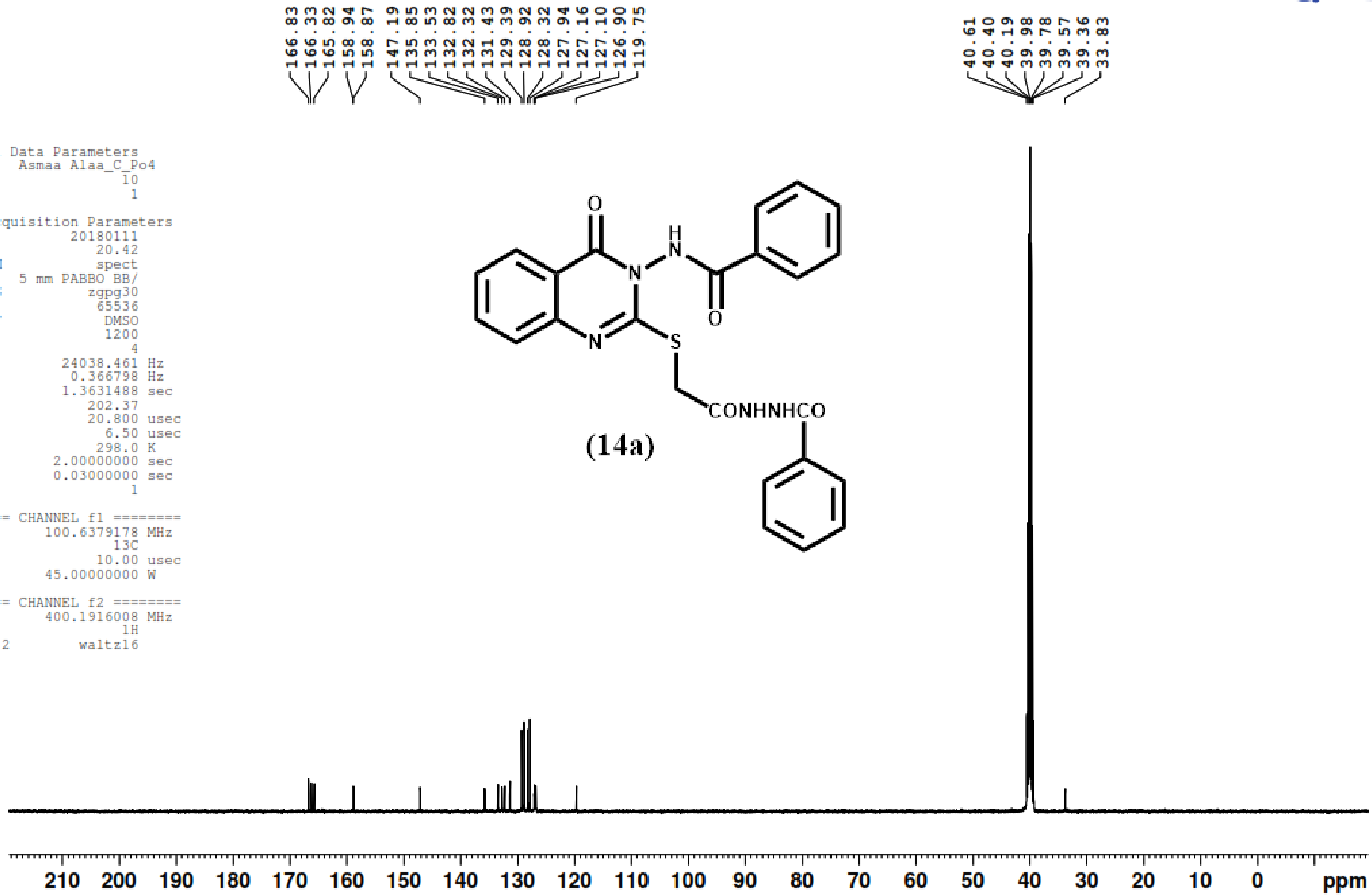

Asmaa Alaa_H_PO5

Microanalytical Unit - FOPCU - NMR laboratory
www.pharma.cu.edu.eg dir-mau.fopcu@pharma.cu.edu.eg

```
Current Data Parameters
NAME       Asmaa Alaa_H_PO5
EXPNO                    10
PROCNO                    1

F2 - Acquisition Parameters
Date_              20180527
Time                  22.31
INSTRUM               spect
PROBHD   5 mm PABBO BB/
PULPROG                zg30
TD                    65536
SOLVENT                DMSO
NS                       32
DS                        2
SWH            8012.820 Hz
FIDRES         0.122266 Hz
AQ            4.0894465 sec
RG                    180.8
DW               62.400 usec
DE                 6.50 usec
TE                298.1 K
D1           1.00000000 sec
TD0                       1

======== CHANNEL f1 ========
SFO1        400.1924713 MHz
NUC1                     1H
P1                15.00 usec
PLW1        10.39999962 W

F2 - Processing parameters
SI                    65536
SF          400.1900000 MHz
WDW                      EM
SSB       0
LB                 0.30 Hz
GB        0
PC                     1.00
```

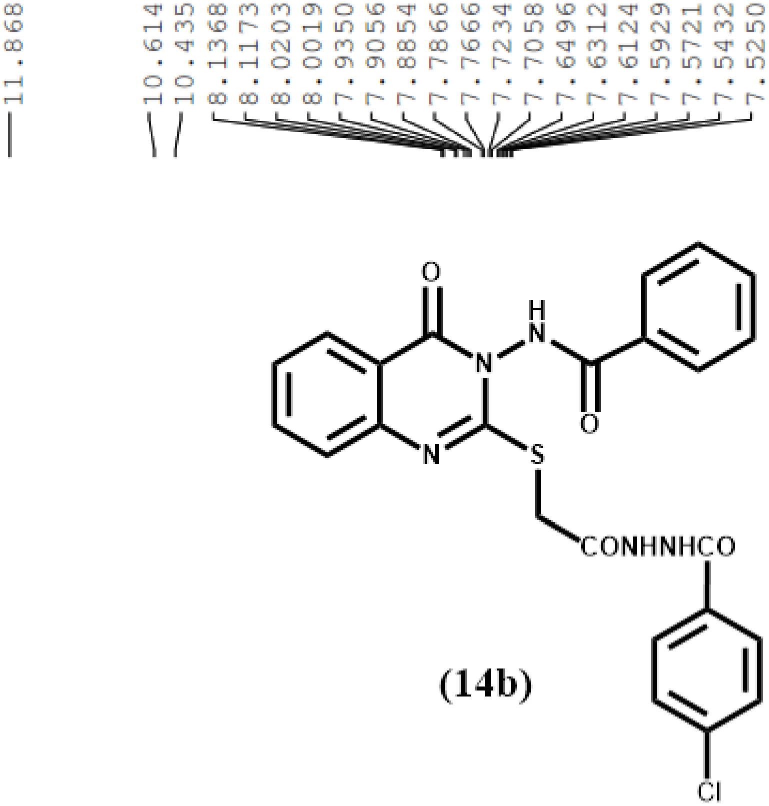

(14b)

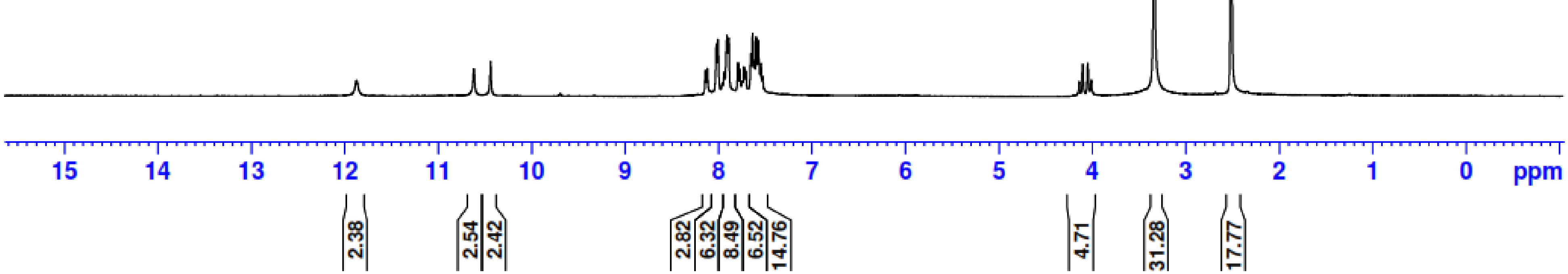

asmaa sakr-po5- carbon

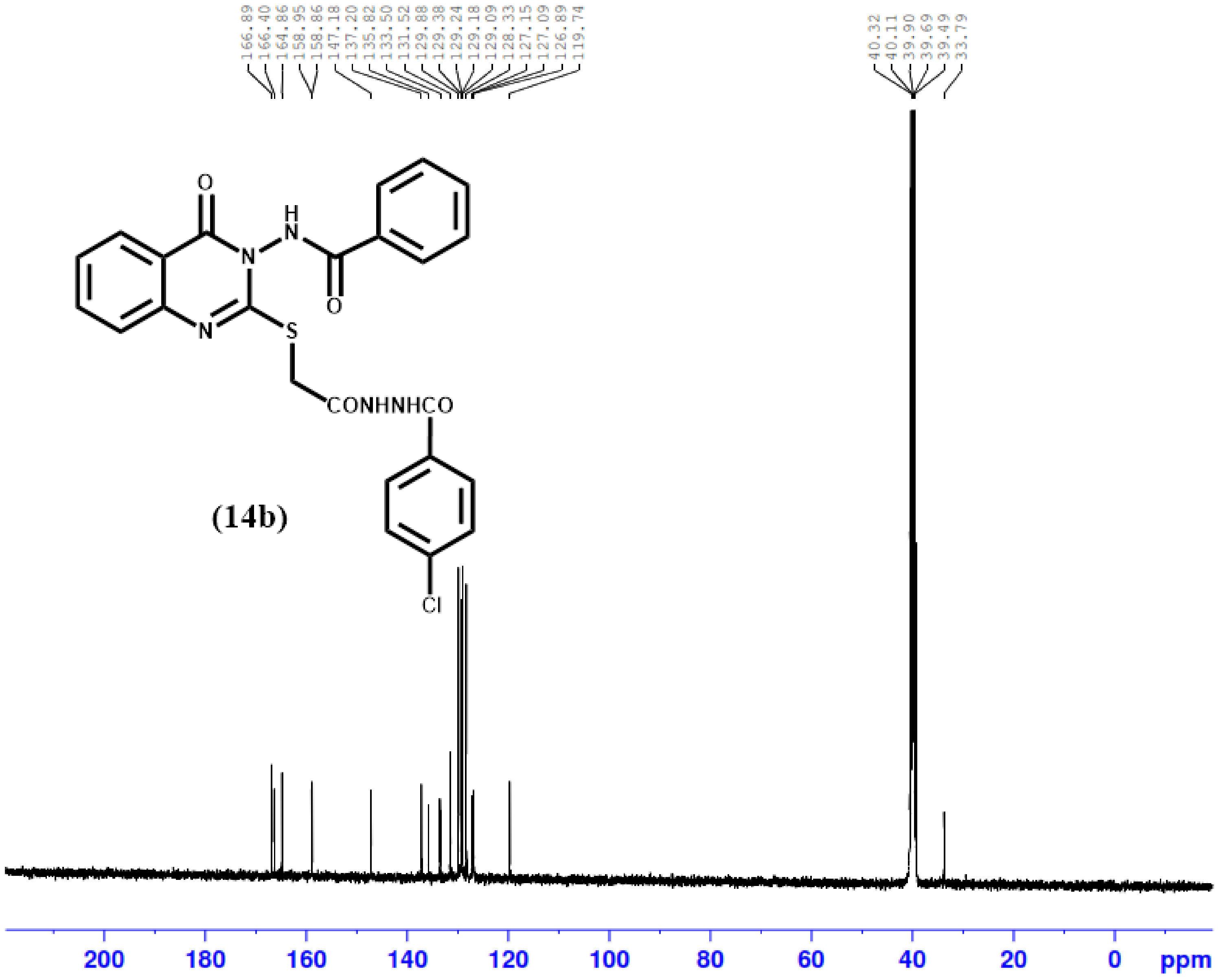

Current Data Parameters

NAME	asmaa sakr-PO5-SS
EXPNO	20
PROCNO	1

F2 - Acquisition Parameters

Date_	20180505
Time	12.57 h
INSTRUM	spect
PROBHD	Z108618_0945 (
PULPROG	zgpg30
TD	65536
SOLVENT	DMSO
NS	1024
DS	4
SWH	24038.461 Hz
FIDRES	0.733596 Hz
AQ	1.3631488 sec
RG	197.77
DW	20.800 usec
DE	6.50 usec
TE	294.3 K
D1	2.00000000 sec
D11	0.03000000 sec
TD0	1
SFO1	100.6404331 MHz
NUC1	13C
P1	10.00 usec
PLW1	47.00000000 W
SFO2	400.2016008 MHz
NUC2	1H
CPDPRG[2	waltz16
PCPD2	90.00 usec
PLW2	13.00000000 W
PLW12	0.29249999 W
PLW13	0.14713000 W

F2 - Processing parameters

SI	32768
SF	100.6303700 MHz
WDW	EM
SSB	0
LB	1.00 Hz
GB	0
PC	1.40

Asmaa Alaa_H_PF2a

BRUKER

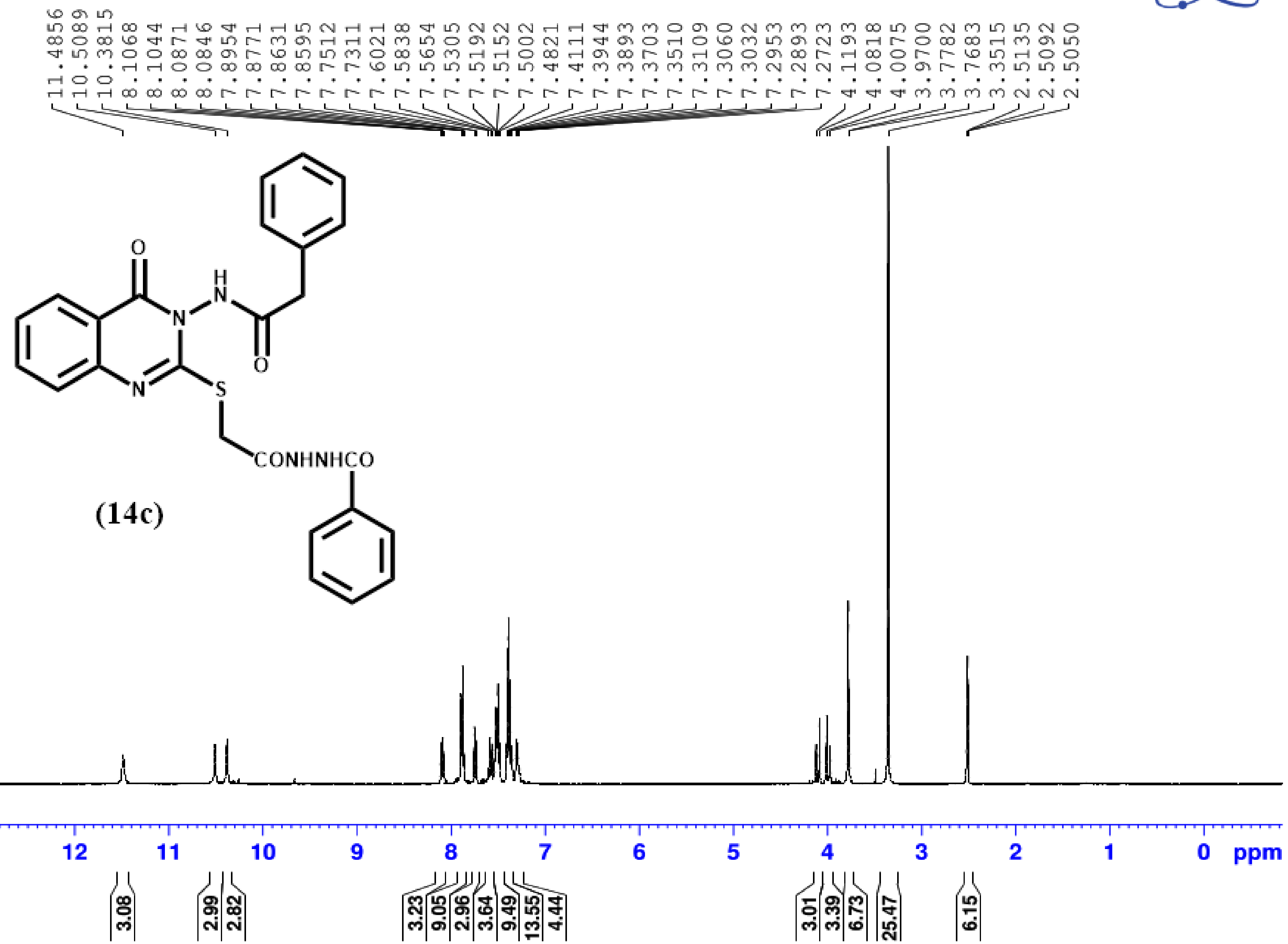

(14c)

```
Current Data Parameters
NAME      Asmaa Alaa_H_PF2a
EXPNO                  10
PROCNO                  1

F2 - Acquisition Parameters
Date_          20170215
Time              15.01
INSTRUM           spect
PROBHD   5 mm PABBO BB/
PULPROG            zg30
TD                65536
SOLVENT            DMSO
NS                   32
DS                    2
SWH            8012.820 Hz
FIDRES         0.122266 Hz
AQ            4.0894465 sec
RG               129.43
DW               62.400 usec
DE                 6.50 usec
TE                298.1 K
D1           1.00000000 sec
TD0                   1

======== CHANNEL f1 ========
SFO1        400.1924713 MHz
NUC1                 1H
P1                15.00 usec
PLW1        10.39999962 W

F2 - Processing parameters
SI                65536
SF          400.1900000 MHz
WDW                  EM
SSB       0
LB                 0.30 Hz
GB        0
PC                 1.00
```

Asmaa Alaa_H_PF2a_D2O

Microanalytical Unit - FOPCU - NMR laboratory
www.pharma.cu.edu.eg dir-mau.fopcu@pharma.cu.edu.eg

BRUKER

```
Current Data Parameters
NAME      Asmaa Alaa_H_PF2a_D2O
EXPNO                 10
PROCNO                 1

F2 - Acquisition Parameters
Date_           20170216
Time               14.31
INSTRUM            spect
PROBHD   5 mm PABBO BB/
PULPROG             zg30
TD                 65536
SOLVENT             DMSO
NS                    32
DS                     2
SWH             8012.820 Hz
FIDRES          0.122266 Hz
AQ             4.0894465 sec
RG                129.43
DW                62.400 usec
DE                  6.50 usec
TE                 298.0 K
D1            1.00000000 sec
TD0                    1

======== CHANNEL f1 ========
SFO1         400.1924713 MHz
NUC1                  1H
P1                 15.00 usec
PLW1         10.39999962 W

F2 - Processing parameters
SI                 65536
SF           400.1900000 MHz
WDW                   EM
SSB       0
LB                  0.30 Hz
GB        0
PC                  1.00
```

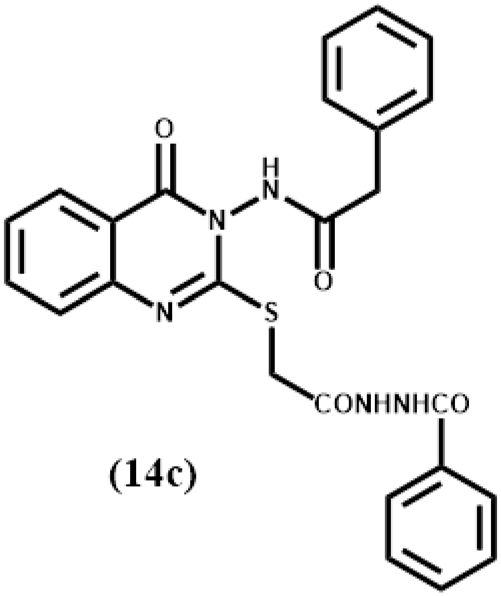

(14c)

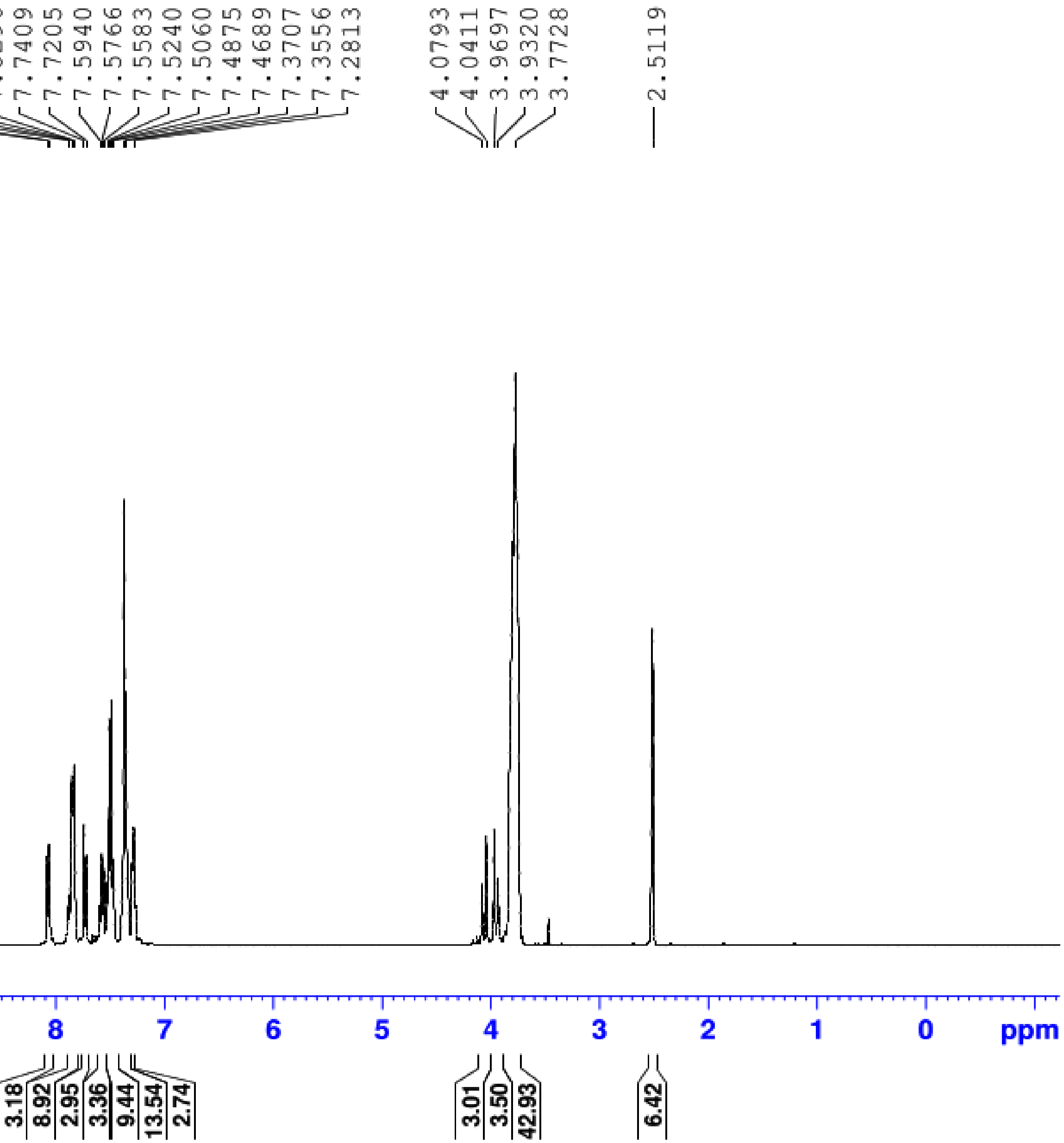

asmaa sakr- pf2a- carbon

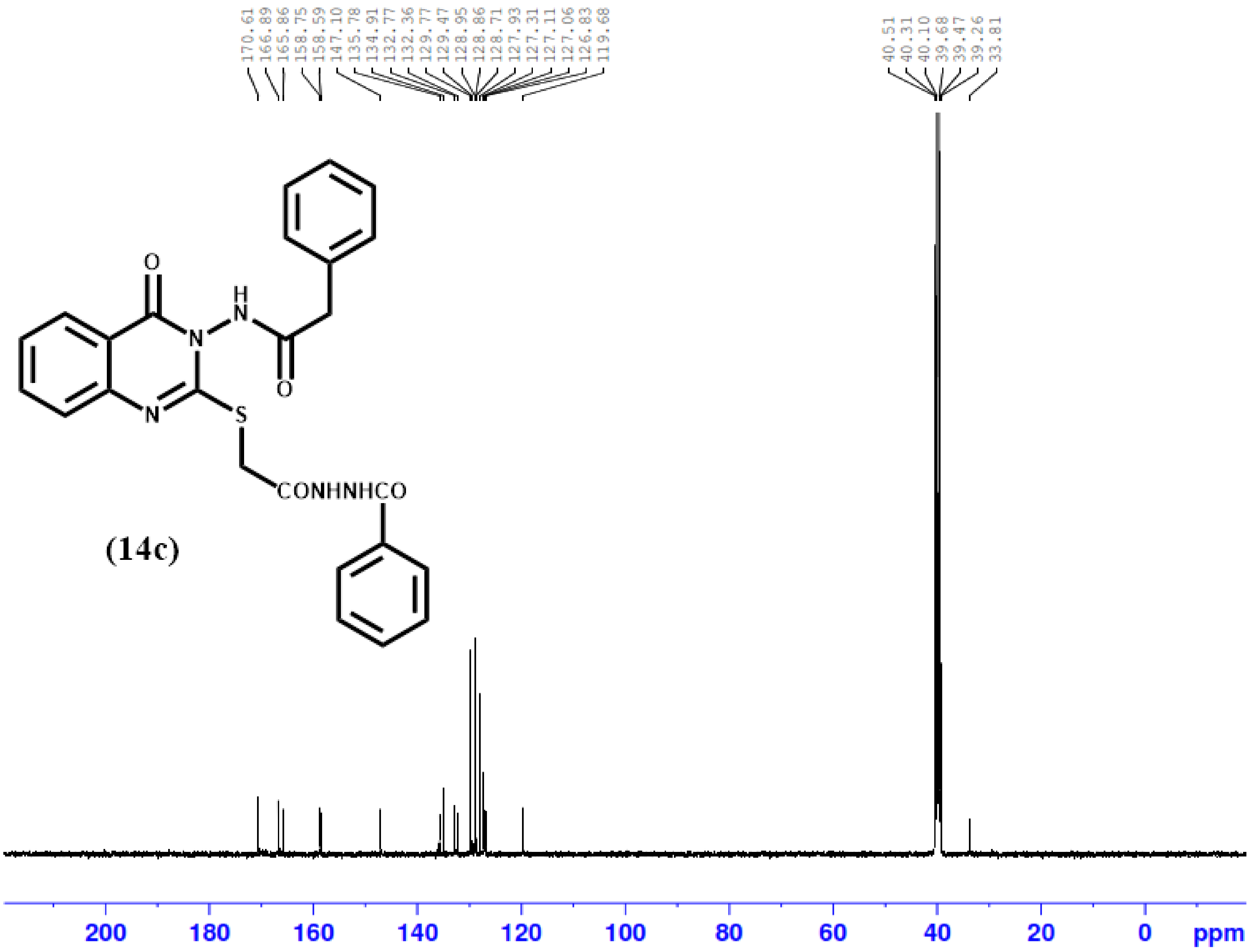

(14c)

Current Data Parameters
NAME asmaa sakr-pf2a-SS
EXPNO 10
PROCNO 1

F2 - Acquisition Parameters
Date_ 20180428
Time 12.47 h
INSTRUM spect
PROBHD Z108618_0945 (
PULPROG zgpg30
TD 65536
SOLVENT DMSO
NS 1024
DS 4
SWH 24038.461 Hz
FIDRES 0.733596 Hz
AQ 1.3631488 sec
RG 197.77
DW 20.800 usec
DE 6.50 usec
TE 294.0 K
D1 2.00000000 sec
D11 0.03000000 sec
TD0 1
SFO1 100.6404331 MHz
NUC1 13C
P1 10.00 usec
PLW1 47.00000000 W
SFO2 400.2016008 MHz
NUC2 1H
CPDPRG[2 waltz16
PCPD2 90.00 usec
PLW2 13.00000000 W
PLW12 0.29249999 W
PLW13 0.14713000 W

F2 - Processing parameters
SI 32768
SF 100.6303700 MHz
WDW EM
SSB 0
LB 1.00 Hz
GB 0
PC 1.40

Asmaa Alaa_H_PO6

Microanalytical Unit - FOPCU - NMR laboratory
www.pharma.cu.edu.eg dir-mau.fopcu@pharma.cu.edu.eg

```
Current Data Parameters
NAME      Asmaa Alaa_H_PO6
EXPNO              10
PROCNO              1

F2 - Acquisition Parameters
Date_          20180527
Time              22.37
INSTRUM           spect
PROBHD   5 mm PABBO BB/
PULPROG            zg30
TD                65536
SOLVENT            DMSO
NS                   32
DS                    2
SWH            8012.820 Hz
FIDRES         0.122266 Hz
AQ            4.0894465 sec
RG                180.8
DW               62.400 usec
DE                 6.50 usec
TE                298.0 K
D1           1.00000000 sec
TD0                   1

======== CHANNEL f1 ========
SFO1        400.1924713 MHz
NUC1                 1H
P1                15.00 usec
PLW1        10.39999962 W

F2 - Processing parameters
SI                65536
SF          400.1900000 MHz
WDW                  EM
SSB      0
LB                 0.30 Hz
GB       0
PC                 1.00
```

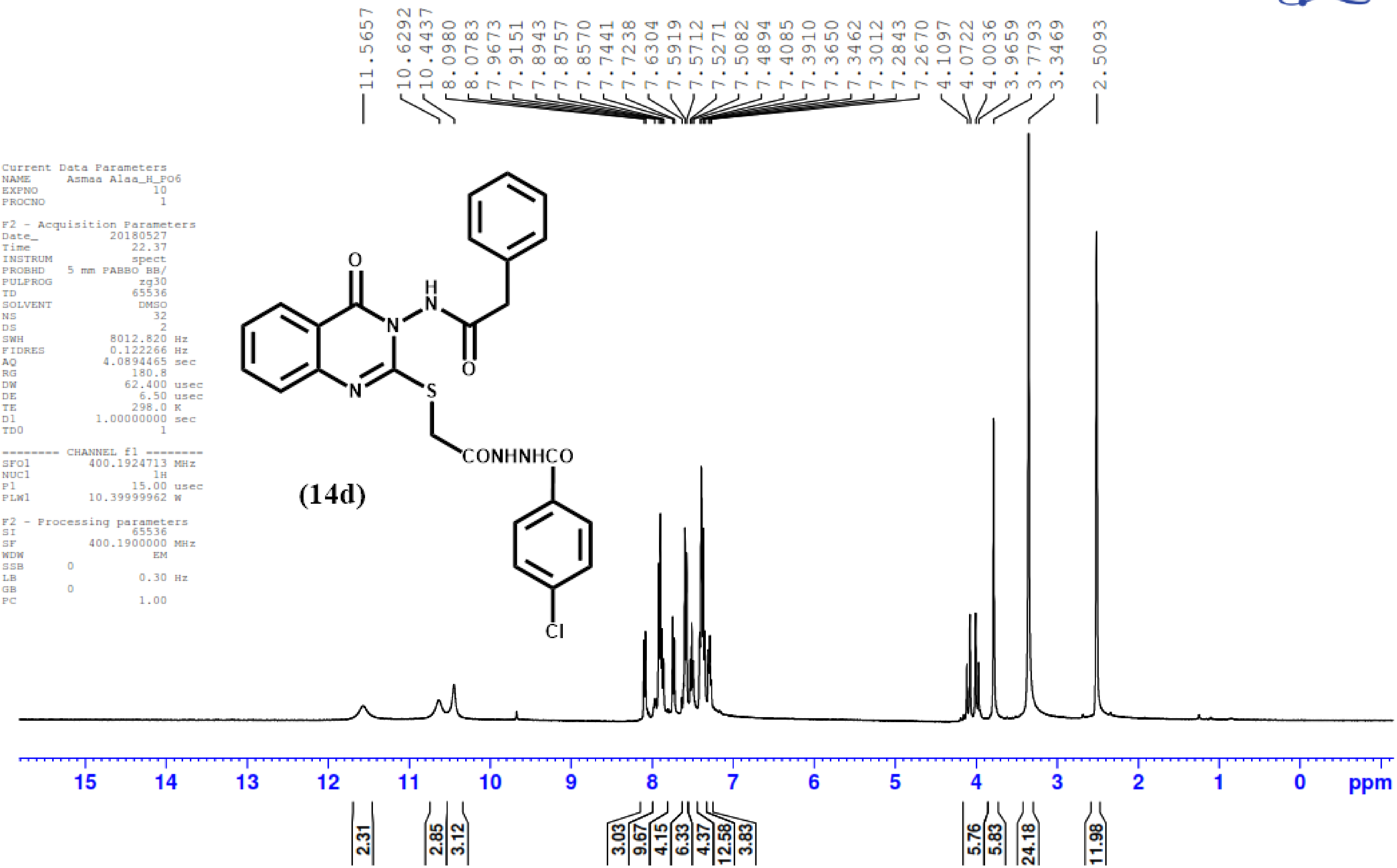

(14d)

Asmaa Alaa_H_PO6_D2O

Microanalytical Unit - FOPCU - NMR laboratory
www.pharma.cu.edu.eg dir-mau.fopcu@pharma.cu.edu.eg

Current Data Parameters
NAME Asmaa Alaa_H_PO6_D2O
EXPNO 10
PROCNO 1

F2 - Acquisition Parameters
Date_ 20180529
Time 12.39
INSTRUM spect
PROBHD 5 mm PABBO BB/
PULPROG zg30
TD 65536
SOLVENT DMSO
NS 32
DS 2
SWH 8012.820 Hz
FIDRES 0.122266 Hz
AQ 4.0894465 sec
RG 180.8
DW 62.400 usec
DE 6.50 usec
TE 298.1 K
D1 1.00000000 sec
TD0 1

======== CHANNEL f1 ========
SFO1 400.1924713 MHz
NUC1 1H
P1 15.00 usec
PLW1 10.39999962 W

F2 - Processing parameters
SI 65536
SF 400.1900000 MHz
WDW EM
SSB 0
LB 0.30 Hz
GB 0
PC 1.00

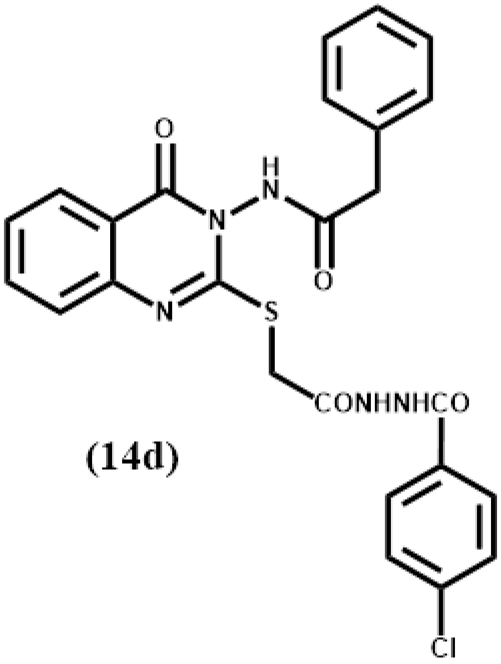

(14d)

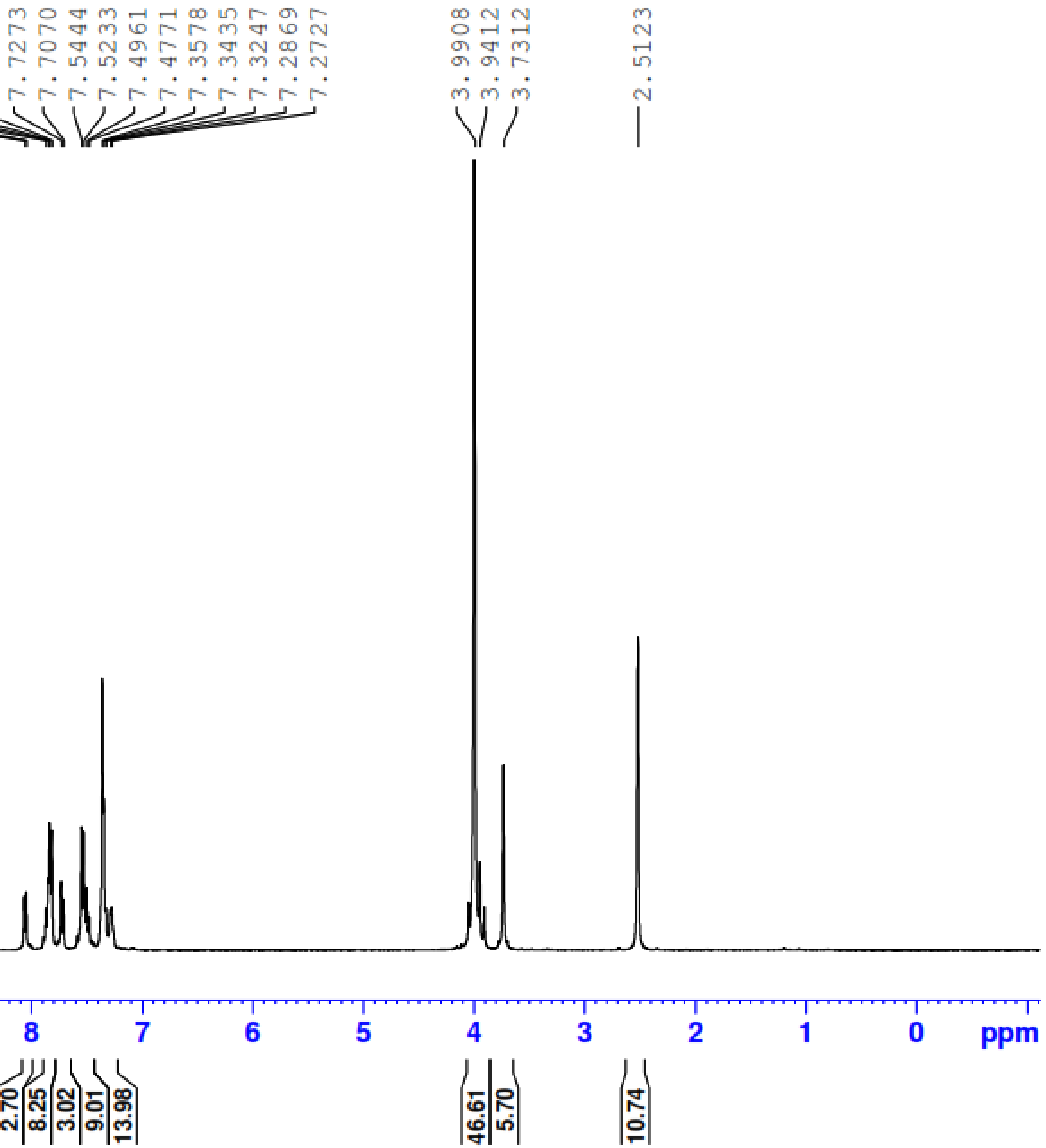

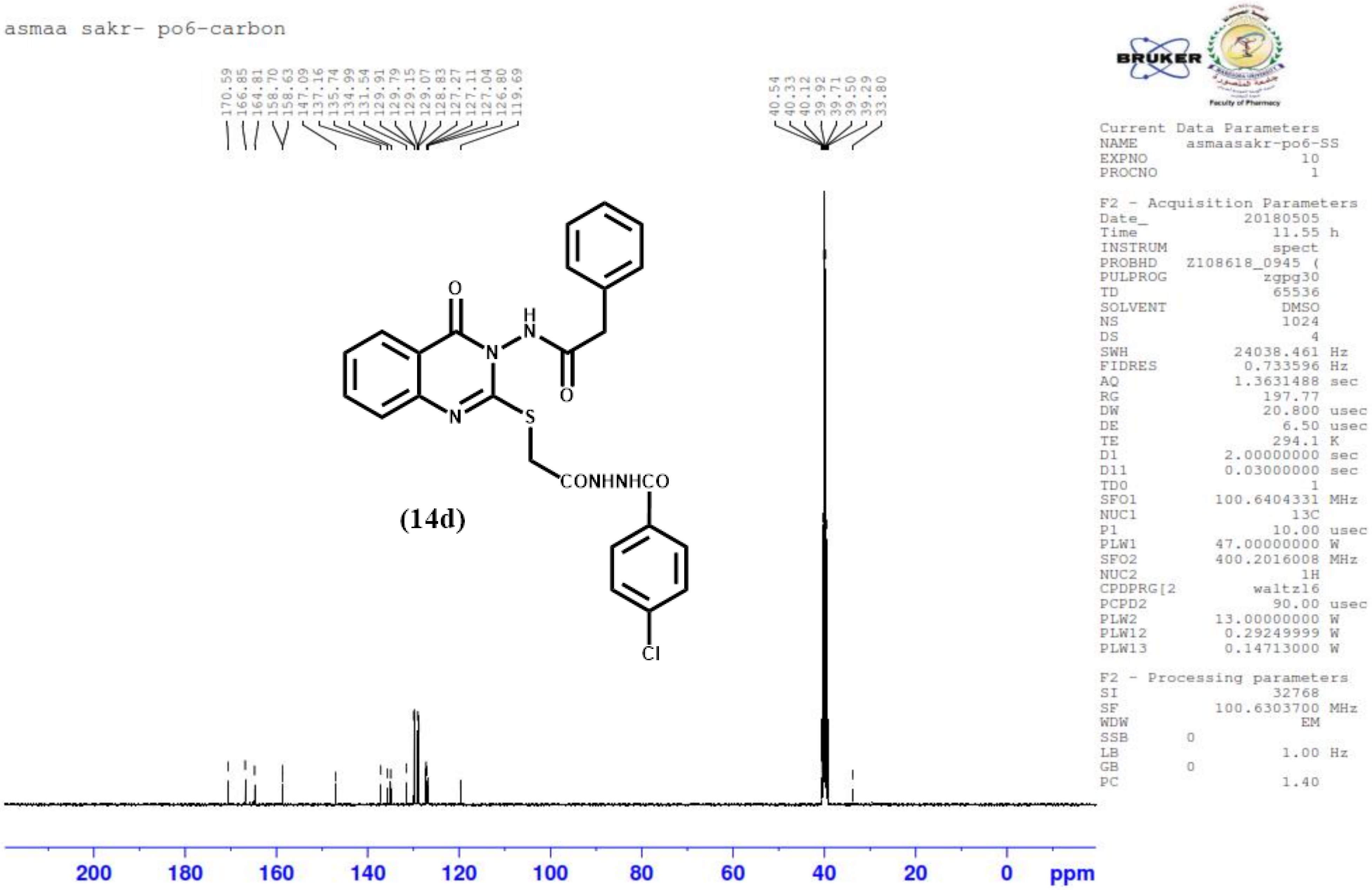
asmaa sakr- po6-carbon
170.59
166.85
164.81
158.70
158.63
147.09
137.16
135.74
134.99
131.54
129.91
129.79
129.15
129.07
128.83
127.27
127.11
127.04
126.80
119.69
40.54
40.33
40.12
39.92
39.71
39.50
39.29
33.80
O
H
N
N
O
N
S
CONHNHCO
Cl
(14d)
200
180
160
140
120
100
80
60
40
20
0
ppm
BRUKER
Faculty of Pharmacy
Current Data Parameters
NAME asmaasakr-po6-SS
EXPNO 10
PROCNO 1
F2 - Acquisition Parameters
Date_ 20180505
Time 11.55 h
INSTRUM spect
PROBHD Z108618_0945 (
PULPROG zgpg30
TD 65536
SOLVENT DMSO
NS 1024
DS 4
SWH 24038.461 Hz
FIDRES 0.733596 Hz
AQ 1.3631488 sec
RG 197.77
DW 20.800 usec
DE 6.50 usec
TE 294.1 K
D1 2.00000000 sec
D11 0.03000000 sec
TD0 1
SFO1 100.6404331 MHz
NUC1 13C
P1 10.00 usec
PLW1 47.00000000 W
SFO2 400.2016008 MHz
NUC2 1H
CPDPRG[2 waltz16
PCPD2 90.00 usec
PLW2 13.00000000 W
PLW12 0.29249999 W
PLW13 0.14713000 W
F2 - Processing parameters
SI 32768
SF 100.6303700 MHz
WDW EM
SSB 0
LB 1.00 Hz
GB 0
PC 1.40

Asmaa Alaa_H_PF2

11.2257
8.9501
8.9289
8.1274
8.1241
8.1079
8.1046
7.8831
7.8793
7.8614
7.8434
7.8396
7.5393
7.5200
7.5016
7.4029
7.3824
7.3644
7.3451
7.3016
7.2847
7.2677
5.8476
3.7470
3.5727
3.3448
2.5132
2.5092

```
Current Data Parameters
NAME       Asmaa Alaa_H_PF2
EXPNO                    10
PROCNO                    1

F2 - Acquisition Parameters
Date_              20170215
Time                  14.56
INSTRUM               spect
PROBHD    5 mm PABBO BB/
PULPROG                zg30
TD                    65536
SOLVENT                DMSO
NS                       32
DS                        2
SWH                8012.820 Hz
FIDRES             0.122266 Hz
AQ                4.0894465 sec
RG                   146.06
DW                   62.400 usec
DE                     6.50 usec
TE                    298.1 K
D1               1.00000000 sec
TD0                       1

======== CHANNEL f1 ========
SFO1            400.1924713 MHz
NUC1                     1H
P1                    15.00 usec
PLW1            10.39999962 W

F2 - Processing parameters
SI                    65536
SF              400.1900000 MHz
WDW                      EM
SSB       0
LB                     0.30 Hz
GB        0
PC                     1.00
```

Unexpected compound

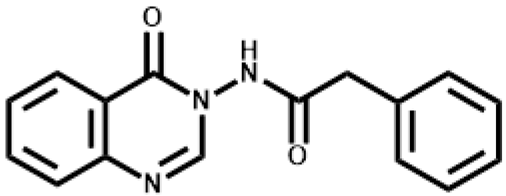

IV

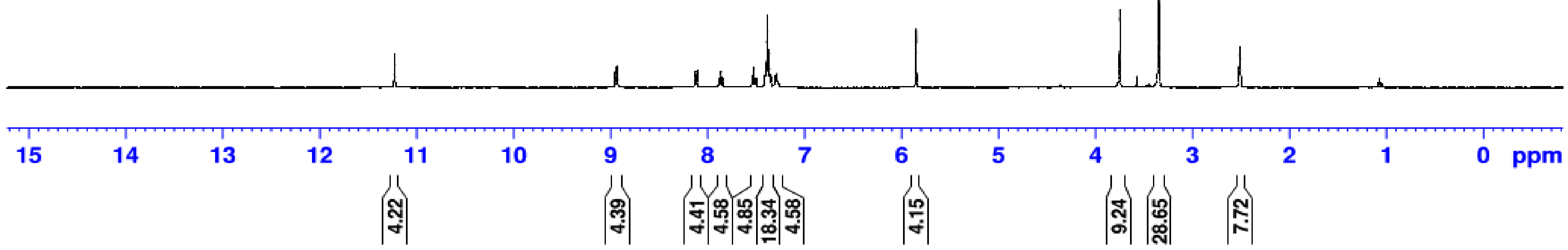

Asmaa Alaa_H_PF2_D2O

Microanalytical Unit - FOPCU - NMR laboratory
www.pharma.cu.edu.eg dir-mau.fopcu@pharma.cu.edu.eg

BRUKER

```
Current Data Parameters
NAME      Asmaa Alaa_H_PF2_D2O
EXPNO               10
PROCNO               1

F2 - Acquisition Parameters
Date_         20170216
Time             14.26
INSTRUM          spect
PROBHD   5 mm PABBO BB/
PULPROG           zg30
TD               65536
SOLVENT           DMSO
NS                  32
DS                   2
SWH           8012.820 Hz
FIDRES        0.122266 Hz
AQ           4.0894465 sec
RG              146.06
DW              62.400 usec
DE                6.50 usec
TE               298.1 K
D1          1.00000000 sec
TD0                  1

======== CHANNEL f1 ========
SFO1       400.1924713 MHz
NUC1                1H
P1               15.00 usec
PLW1       10.39999962 W

F2 - Processing parameters
SI               65536
SF         400.1900000 MHz
WDW                 EM
SSB       0
LB                0.30 Hz
GB        0
PC                1.00
```

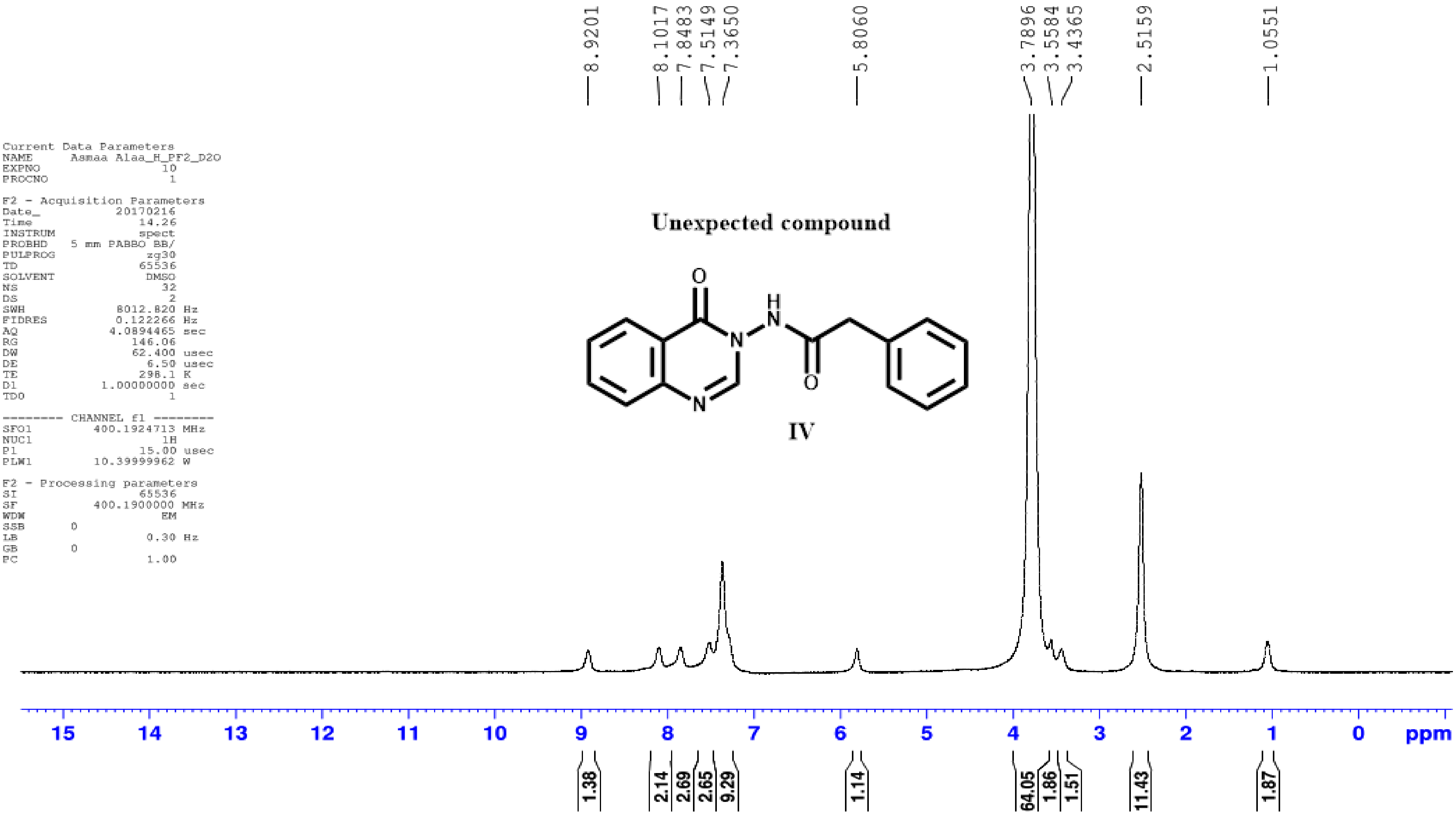

Table.1: Molecular docking details of target compounds with both (1CX2 and 1EQG):

Code	Docking of target compounds and **1CX2**				Docking of target compounds and **1EQG**			
	Number of bonds	Type of bond	Docking scores (Kcal/mol)	Amino acid interactions	Number of bonds	Type of bond	Docking scores (Kcal/mol)	Amino acid interactions
**4a**	1	1 HB [a]	-6.2604	Ala527	1	1 HB [a]	-5.9148	Arg120
**4b**	1	1 arene-H [b]	-7.0540	Ser353	2	2 HB [a]	-4.2701	Tyr355, Val116
**7c**	1	2 HB [a]	-6.9140	Val523, Arg120	-	-	-6.4143	-
**13b**	1	1 HB [a]	-8.4080	Arg120	2	1 HB [a], 1arene-H [b]	-2.0562	Arg120, Tyr355

**14c**	3	3 HB [a]	-8.5355	Arg513, Gly354, Ser353	1	1HB [a]	-6.3893	Arg120
**SC-558**	5	4 HB [a], 1 arene-H [b]	-9.3983	Ty355, Arg120, Arg513, His90, ser353	**-**	**-**	-1.3609	-
**Ibuprofen**					3	HB [a]		Arg120, Tyr355

* [a] (HB) is symbol for hydrogen bond, [b] (arene-H); arene group attached with hydrogen bond.

Table.2: predict bioavailability using online website (MOLINSPIRATION): www.molinspiration.com

Code	Log p	M.WT	nON (HBA)	nOHNH (HBD)	LIPINSK'S VIOLATION	TPSA	%ABS	VOLUMES	NROTB
**4a**	4.57	444.92	6	2	0	68.44	85.39	384.66	4
**4b**	3.85	455.47	9	2	0	114.26	69.58	394.46	5
**7c**	5.80	486.57	8	1	1	98.47	75.03	448.82	7
**13b**	0.51	383.43	8	4	0	119.12	41.09	325.43	6
**14c**	2.75	487.54	9	3	0	122.19	66.84	416.94	8
**Celecoxib**	3.61	381.38	5	2	0	77.99	82.09	298.65	4
**Ibuprofen**	3.46	206.28	2	1	0	37.30	96.13	211.19	4
**indomethacin**	3.99	357.79	5	1	0	68.54	85.35	303.24	4

7c Lipinski violation was Log p which must be <5 or equal and in this compound it >5 log p= 5.80

Reference for equation:

https://www.ncbi.nlm.nih.gov/pubmed/12425461

The values of TPSA are used to calculate the percentage of oral absorption (%ABS) using the following equation:

%ABS= 109 - 0.345 TPSA

Table.3: Results of prediction of drug cell permeability, intestinal, BBB and PPB using Pre-ADMET calculator.

code	Caco2	MDCK	HIA	BBB	PPB
**4a**	34.04	0.42	93.96	4.07	90.25
**4b**	19.75	0.08	92.79	0.31	90.57
**7c**	21.15	0.06	96.97	0.05	90.37
**13b**	12.58	1.79	91.51	0.08	88.69
**14c**	11.24	0.07	93.56	0.03	100.00
**Celecoxib**	0.499	45.05	96.69	0.03	91.07
**Ibuprofen**	21.21	136.48	98.38	1.27	88.25
**indomethacin**	20.14	3.81*	98.44	0.03	78.06

All compounds showed the same results as 3 reference drugs (Celecoxib, Ibuprofen and indomethacin) as **non**-inhibitor activity for (CYP2D6 enzyme),so they will be expected to not included in any Drug-Drug interaction for both inhibitors and/or inducers of that enzyme.
